# Supplementary material for: Neuronal XBP-1 Activates Intestinal Lysosomes to Improve Proteostasis in C. elegans
Source: Curr Biol. 2019 Jul 22;29(14):2322–2338.e7. doi: 10.1016/j.cub.2019.06.031 (PMC6658570; doi:10.1016/j.cub.2019.06.031)
Supplement: Document S1. Figures S1–S7 [file mmc1.pdf]

Current Biology, Volume *29*

Supplemental Information

Neuronal XBP-1 Activates Intestinal Lysosomes to Improve Proteostasis in *C. elegans*

Soudabeh Imanikia, Neşem P. Özbey, Christel Krueger, M. Olivia Casanueva, and Rebecca C. Taylor

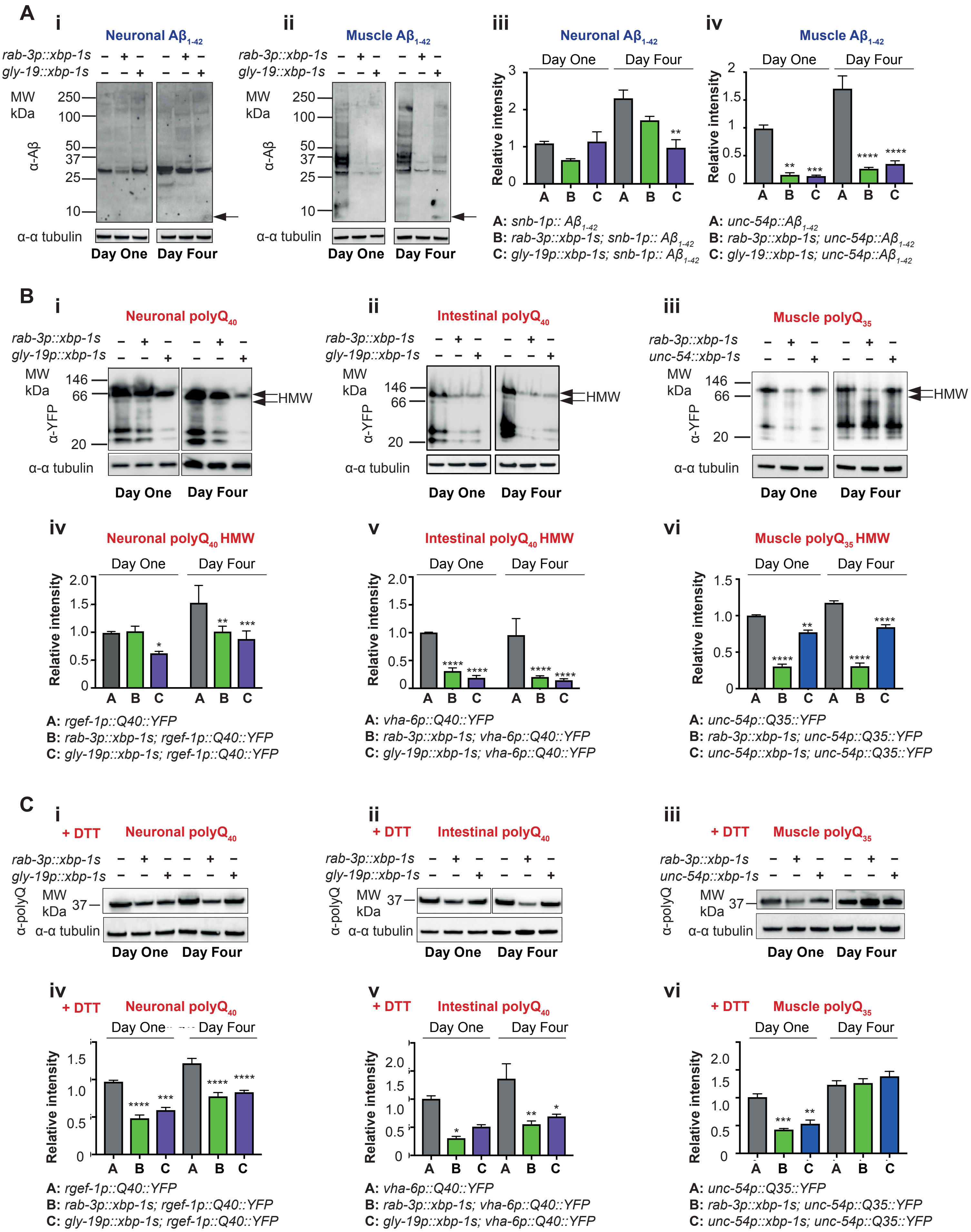
A
i
Neuronal Aβ1-42
rab-3p::xbp-1s
gly-19::xbp-1s
MW kDa
250
100
50
37
25
10
α-Aβ
α-α tubulin
Day One
Day Four
ii
Muscle Aβ1-42
rab-3p::xbp-1s
gly-19::xbp-1s
MW kDa
α-Aβ
α-α tubulin
Day One
Day Four
iii
Neuronal Aβ1-42
Day One
Day Four
Relative intensity
A B C A B C
A: snb-1p:: Aβ1-42
B: rab-3p::xbp-1s; snb-1p:: Aβ1-42
C: gly-19p::xbp-1s; snb-1p:: Aβ1-42
iv
Muscle Aβ1-42
Day One
Day Four
Relative intensity
A B C A B C
A: unc-54p::Aβ1-42
B: rab-3p::xbp-1s; unc-54p::Aβ1-42
C: gly-19::xbp-1s; unc-54p::Aβ1-42
B
i
Neuronal polyQ40
rab-3p::xbp-1s
gly-19p::xbp-1s
MW kDa
146
66
20
α-YFP
HMW
α-α tubulin
Day One
Day Four
ii
Intestinal polyQ40
rab-3p::xbp-1s
gly-19p::xbp-1s
MW kDa
α-YFP
HMW
α-α tubulin
Day One
Day Four
iii
Muscle polyQ35
rab-3p::xbp-1s
unc-54::xbp-1s
MW kDa
α-YFP
HMW
α-α tubulin
Day One
Day Four
iv
Neuronal polyQ40 HMW
Day One
Day Four
Relative intensity
A B C A B C
A: rgef-1p::Q40::YFP
B: rab-3p::xbp-1s; rgef-1p::Q40::YFP
C: gly-19p::xbp-1s; rgef-1p::Q40::YFP
v
Intestinal polyQ40 HMW
Day One
Day Four
Relative intensity
A B C A B C
A: vha-6p::Q40::YFP
B: rab-3p::xbp-1s; vha-6p::Q40::YFP
C: gly-19p::xbp-1s; vha-6p::Q40::YFP
vi
Muscle polyQ35 HMW
Day One
Day Four
Relative intensity
A B C A B C
A: unc-54p::Q35::YFP
B: rab-3p::xbp-1s; unc-54p::Q35::YFP
C: unc-54p::xbp-1s; unc-54p::Q35::YFP
C
i
+ DTT
Neuronal polyQ40
rab-3p::xbp-1s
gly-19p::xbp-1s
α-polyQ
MW kDa
37
α-α tubulin
Day One
Day Four
ii
+ DTT
Intestinal polyQ40
rab-3p::xbp-1s
gly-19p::xbp-1s
α-polyQ
MW kDa
37
α-α tubulin
Day One
Day Four
iii
+ DTT
Muscle polyQ35
rab-3p::xbp-1s
unc-54p::xbp-1s
α-polyQ
MW kDa
37
α-α tubulin
Day One
Day Four
iv
+ DTT
Neuronal polyQ40
Day One
Day Four
Relative intensity
A B C A B C
A: rgef-1p::Q40::YFP
B: rab-3p::xbp-1s; rgef-1p::Q40::YFP
C: gly-19p::xbp-1s; rgef-1p::Q40::YFP
v
+ DTT
Intestinal polyQ40
Day One
Day Four
Relative intensity
A B C A B C
A: vha-6p::Q40::YFP
B: rab-3p::xbp-1s; vha-6p::Q40::YFP
C: gly-19p::xbp-1s; vha-6p::Q40::YFP
vi
+ DTT
Muscle polyQ35
Day One
Day Four
Relative intensity
A B C A B C
A: unc-54p::Q35::YFP
B: rab-3p::xbp-1s; unc-54p::Q35::YFP
C: unc-54p::xbp-1s; unc-54p::Q35::YFP

Figure S1. Neuronal and intestinal *xbp-1s* reduce levels of toxic protein species. Related to Figure 2.

(A) Reducing Western blot analysis of (i) neuronal $A\beta_{1-42}$ and (ii) muscle $A\beta_{1-42}$ with and without neuronal and intestinal *xbp-1s,* at day 1 and day 4 of adulthood. Lysates were resolved under reducing conditions and blotted with an anti-Aβ antibody; the predicted MW of monomeric $A\beta_{1-42}$ is ~4.5 kDa, indicated with an arrow. Tubulin levels were probed with α-α tubulin as a loading control. Blots were quantified using ImageJ (iii-iv). Graphs represent mean lane intensity relative to day 1 $A\beta_{1-42}$ ± SD and statistical significance was calculated between A and B/C at each time point using two-way ANOVA with Tukey's multiple comparisons test, $^{**}p<0.01$, $^{***}p<0.001$, $^{****}p<0.0001$. Data are representative of 3 independent experiments.

(B) Native Western blot analysis of (i) neuronal $polyQ_{40}$::YFP, (ii) intestinal $polyQ_{40}$::YFP and (iii) muscle $polyQ_{35}$::YFP with and without tissue-specific *xbp-1s,* at day 1 and day 4 of adulthood. Lysates were resolved under native conditions and blotted with an anti-YFP/GFP antibody. The predicted MW of monomeric $polyQ_{35\text{-}40}$:YFP is ~32 kDa – HMW indicates the higher molecular weight, polyQ-reactive band, while lower molecular weight, non-polyQ-reactive bands are likely to represent YFP cleaved from polyQ. Tubulin levels were probed with α-α tubulin as a loading control. Data are representative of at least 2 independent experiments. Blots were quantified using ImageJ (iv-vi). Bar graphs represent mean band intensity relative to day 1 polyQ ± standard deviation (SD) and statistical significance was calculated between A and B/C at each time point using two-way ANOVA with Tukey's multiple comparisons test, $^{*}p<0.05$, $^{**}p<0.01$, $^{***}p<0.001$, $^{****}p<0.0001$.

(C) Reducing Western blot analysis of (i) neuronal polyQ$_{40}$::YFP, (ii) intestinal polyQ$_{40}$::YFP and (iii) muscle polyQ$_{35}$::YFP expressed with and without tissue-specific *xbp-1s,* at day 1 and day 4 of adulthood. Lysates were resolved under reducing conditions and blotted with an anti-polyQ antibody; the predicted MW of monomeric polyQ$_{35-40}$:YFP is ~32 kDa. Tubulin levels were probed with α-α tubulin as a loading control. Blots were quantified using ImageJ (iv-vi). Bar graphs represent mean band intensity relative to day 1 polyQ ± SD and statistical significance was calculated between A and B/C at each time point using a two-way ANOVA with Tukey's multiple comparisons test, *p<0.05, **p<0.01, ***p<0.001, ****p<0.0001. Data are representative of at least 3 independent experiments.

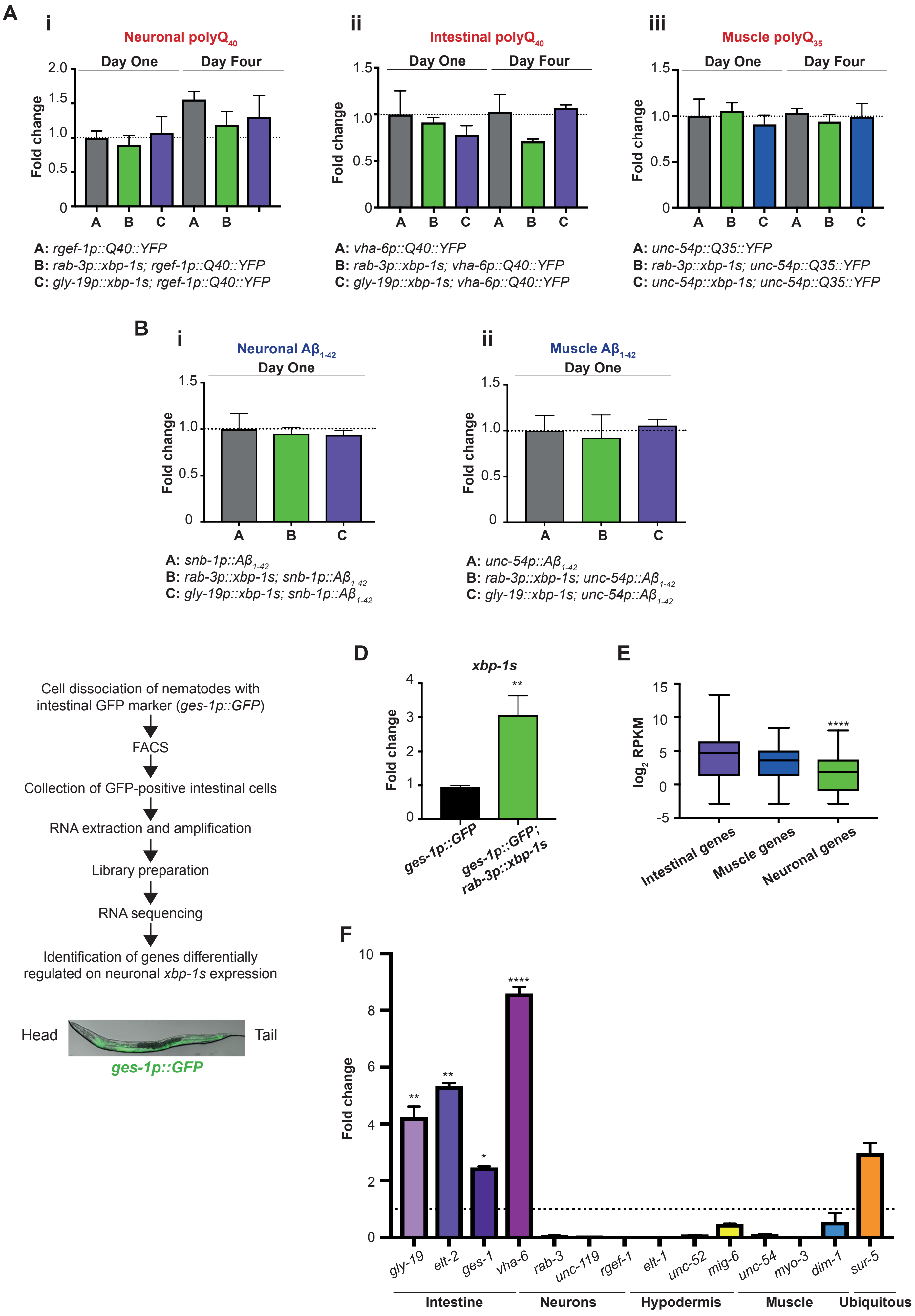
A
i
Neuronal polyQ40
Day One
Day Four
Fold change
A B C A B
A: rgef-1p::Q40::YFP
B: rab-3p::xbp-1s; rgef-1p::Q40::YFP
C: gly-19p::xbp-1s; rgef-1p::Q40::YFP
ii
Intestinal polyQ40
Day One
Day Four
Fold change
A B C A B C
A: vha-6p::Q40::YFP
B: rab-3p::xbp-1s; vha-6p::Q40::YFP
C: gly-19p::xbp-1s; vha-6p::Q40::YFP
iii
Muscle polyQ35
Day One
Day Four
Fold change
A B C A B C
A: unc-54p::Q35::YFP
B: rab-3p::xbp-1s; unc-54p::Q35::YFP
C: unc-54p::xbp-1s; unc-54p::Q35::YFP
B
i
Neuronal Aβ1-42
Day One
Fold change
A B C
A: snb-1p::Aβ1-42
B: rab-3p::xbp-1s; snb-1p::Aβ1-42
C: gly-19p::xbp-1s; snb-1p::Aβ1-42
ii
Muscle Aβ1-42
Day One
Fold change
A B C
A: unc-54p::Aβ1-42
B: rab-3p::xbp-1s; unc-54p::Aβ1-42
C: gly-19::xbp-1s; unc-54p::Aβ1-42
C
Cell dissociation of nematodes with intestinal GFP marker (ges-1p::GFP)
FACS
Collection of GFP-positive intestinal cells
RNA extraction and amplification
Library preparation
RNA sequencing
Identification of genes differentially regulated on neuronal xbp-1s expression
Head
Tail
ges-1p::GFP
D
xbp-1s
Fold change
**
ges-1p::GFP
ges-1p::GFP; rab-3p::xbp-1s
E
log2 RPKM
****
Intestinal genes
Muscle genes
Neuronal genes
F
Fold change
**
**
*
****
gly-19
elt-2
ges-1
vha-6
rab-3
unc-119
rgef-1
elt-1
unc-52
mig-6
unc-54
myo-3
dim-1
sur-5
Intestine
Neurons
Hypodermis
Muscle
Ubiquitous

Figure S2. Measurement of polyQ and A$\beta_{1\text{-}42}$ transcript levels and validation of intestine-specific RNA-seq analysis. Related to Figures 2 and 3.

(A) qPCR of YFP transcript levels in animals expressing (i) neuronal polyQ$_{40}$::YFP, (ii) intestinal polyQ$_{40}$::YFP and (iii) muscle polyQ$_{35}$::YFP expressed with and without tissue-specific *xbp-1s.* YFP expression was measured at day 1 and day 4 of adulthood and normalized to day 1 polyQ alone. Significance was measured by one-way ANOVA with Tukey's multiple comparisons test.

(B) qPCR of A$\beta_{1\text{-}42}$ transcript levels in animals expressing (i) neuronal A$\beta_{1\text{-}42}$ and (ii) muscle A$\beta_{1\text{-}42}$ with and without neuronal and intestinal *xbp-1s.* A$\beta_{1\text{-}42}$ expression was measured at day 1 of adulthood and *xbp-1s*-expressing animals normalized to A$\beta_{1\text{-}42}$ alone. Significance was measured by one-way ANOVA with Tukey's multiple comparisons test.

(C) Workflow of intestine-specific RNA-seq analysis. Cells from nematodes expressing the *ges-1p::GFP* intestinal marker are dissociated and collected using fluorescence-activated cell sorting (FACS). RNA is then extracted and amplified using the Ovation RNA-Seq System V2 before library preparation using the Ovation® Ultralow Library System. This RNA is sequenced and analyzed by intensity difference filtration to identify genes that are differentially regulated between *ges-1p::GFP* worms with and without neuronal *xbp-1s*. A confocal overlay micrograph of *ges-1p::GFP* worms is shown at X10 magnification.

(D) qPCR of *xbp-1s* transcript levels in intestinal cells of wild type and *rab-3p::xbp-1s-* expressing animals. Intestinal cells were isolated at day 1 of

adulthood from animals expressing *ges-1p::GFP* with and without *rab-3p::xbp-1s*, *xbp-1s* transcript levels measured, and *xbp-1*-expressing animals normalized to *ges-1p::GFP* alone. Significance was measured by one-way ANOVA with Tukey's multiple comparisons test, **p<0.01.

(E) Box and whisker plots of intestinal, muscle and neuronal gene expression levels in the RNA-seq dataset. Tissue-specific genes were identified using the Princeton "Tissue-specific expression predictions for *C. elegans*" database (http://worm-tissue.princeton.edu/search/download) and the distribution of expression levels of these genes in RNA-seq samples plotted. Significance between intestine-specific and muscle- or neuron-specific gene categories was measured by student's t-test, using expression of each tissue-specific gene as one sample, ****p<0.0001.

(F) Validation of intestinal enrichment by qRT-PCR analysis of tissue-specific transcripts. Levels of 4 intestinal transcripts - *gly-19, elt-2, ges-1, vha-6*; 3 neuronal transcripts - *rab-3, unc-119, rgef-1*; 3 hypodermal transcripts – *elt-1, unc-52, mig-6*; 3 muscle transcripts - *unc-54, myo-3, dim-1*; and 1 ubiquitous transcript - *sur-5*; were measured by qRT-PCR in RNA extracted from *ges-1p::GFP*-expressing isolated intestinal cells, and in RNA extracted from non-intestinal GFP-negative cells. Graphs represent mean transcript levels in sorted intestinal cells normalized to levels of each transcript in GFP-negative cells, from 3 independent biological replicates. Error bars represent standard error of the mean (SEM). Significance of enrichment was assessed by one-way ANOVA with Tukey's multiple comparisons test, *p<0.05, **p<0.001, ****p<0.0001.

A

Upregulated lysosomal gene	XBP-1s promoter binding sites (5’--3’)	HLH-30 promoter binding sites (5’--3’)
asp-3	ACGT core (-86 to -80)	-
cpr-1	ETS domain (-316 to -311; -88 to -83); ACGT core (-1975 to -1969; -730 to -724); UPRE B element (-1979 to -1969)	CLEAR domain (-152 to -147)
cpr-2	CCACG box (-827 to -822); ACGT core (-702 to -696)	-
cpr-4	ETS domain (-1859 to -1854); CCACG box (-1013 to -1008); ACGT core (-1042 to -1036; -459 to -453)	-
cpr-5	-	-
imp-2	CAAT box (-341 to -336)	CLEAR domain (-311 to -306)
F57F5.1	ACGT core (-373 to -367; -370 to -364)	CLEAR domain (-53 to -48)
aagr-3	CAAT box (+28 to +33); ACGT core (-427 to -421)	-
asah-1	CCACG box (-274 to -269; -82 to -77); ACGT core (-82 to -76)	CLEAR domain (-80 to -75)
gba-2	CAAT box (-802 to -797); ETS domain (-507 to -502)	-
hex-1	CAAT box (-353 to -348; -212 to -207); CCACG box (-1494 to -1489)	-
lipl-1	CCACG box (-985 to -980)	CLEAR domain (-444 to -439)
lipl-5	ACGT core (-1091 to -1085)	CLEAR domain (-51 to -46)
spp-10	-	CLEAR domain (-847 to -842)
nuc-1	ETS domain (-1617 to -1612); ACGT core (-295 to -289)	-
cdr-1	-	-
lys-4	-	CLEAR domain (-838 to -833)
lys-8	-	-
ctns-1	ETS domain (-1559 to -1554; -1827 to -1822)	-
vha-18	-	-
blos-8	CAAT box (-1430 to -1425)	-

Binding sites
CAAT box (5' -CCAATC- 3')
ETS domain (5' -CGGAAG- 3')
CCACG box (5' -GCCACG- 3')
ACGT core (5' -G(C/T)(C/G)ACGT- 3')
UPRE A element (5' -CGACGTGG- 3')
UPRE B element (5' -GTGACGTG- 3')
CLEAR domain (5' -CACGTG- 3')

B

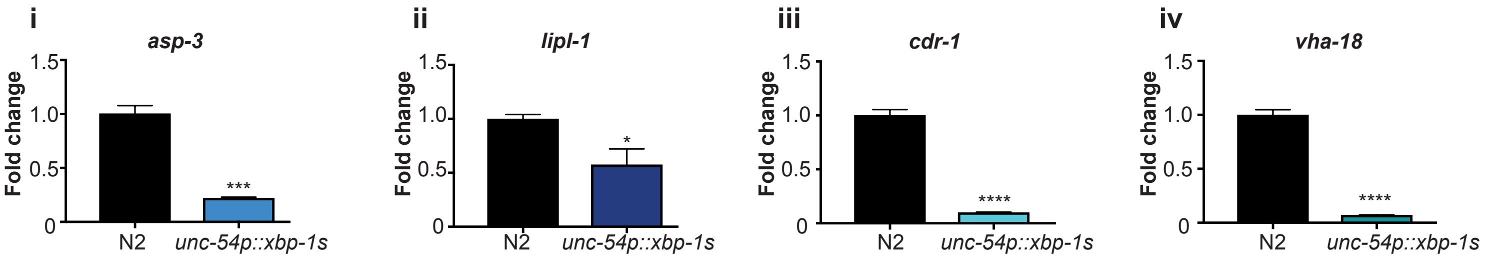

Figure S3. Lysosomal genes may be direct and tissue-specific targets of *xbp-1s*. Related to Figure 3.

(A) *xbp-1s* and *hlh-30* binding sites in the promoters of lysosomal genes upregulated in the intestines of neuronal *xbp-1s*-expressing animals. Sites listed in the legend were searched for within promoter sequences as defined by the Harvard Promoterome Database.

(B) qRT-PCR of lysosomal gene transcript levels in all-tissue lysates from animals expressing *xbp-1s* in muscle cells relative to N2 nematodes: (i) *asp-3*, (ii) *lipl-1*, (iii) *cdr-1*, (iv) *vha-18*. Transcript levels were measured at day 1 of adulthood and *xbp-1*-expressing animals normalized to N2. Significance was measured by one-way ANOVA with Tukey's multiple comparisons test, $^{*}p<0.05$, $^{***}p<0.001$, $^{****}p<0.0001$.

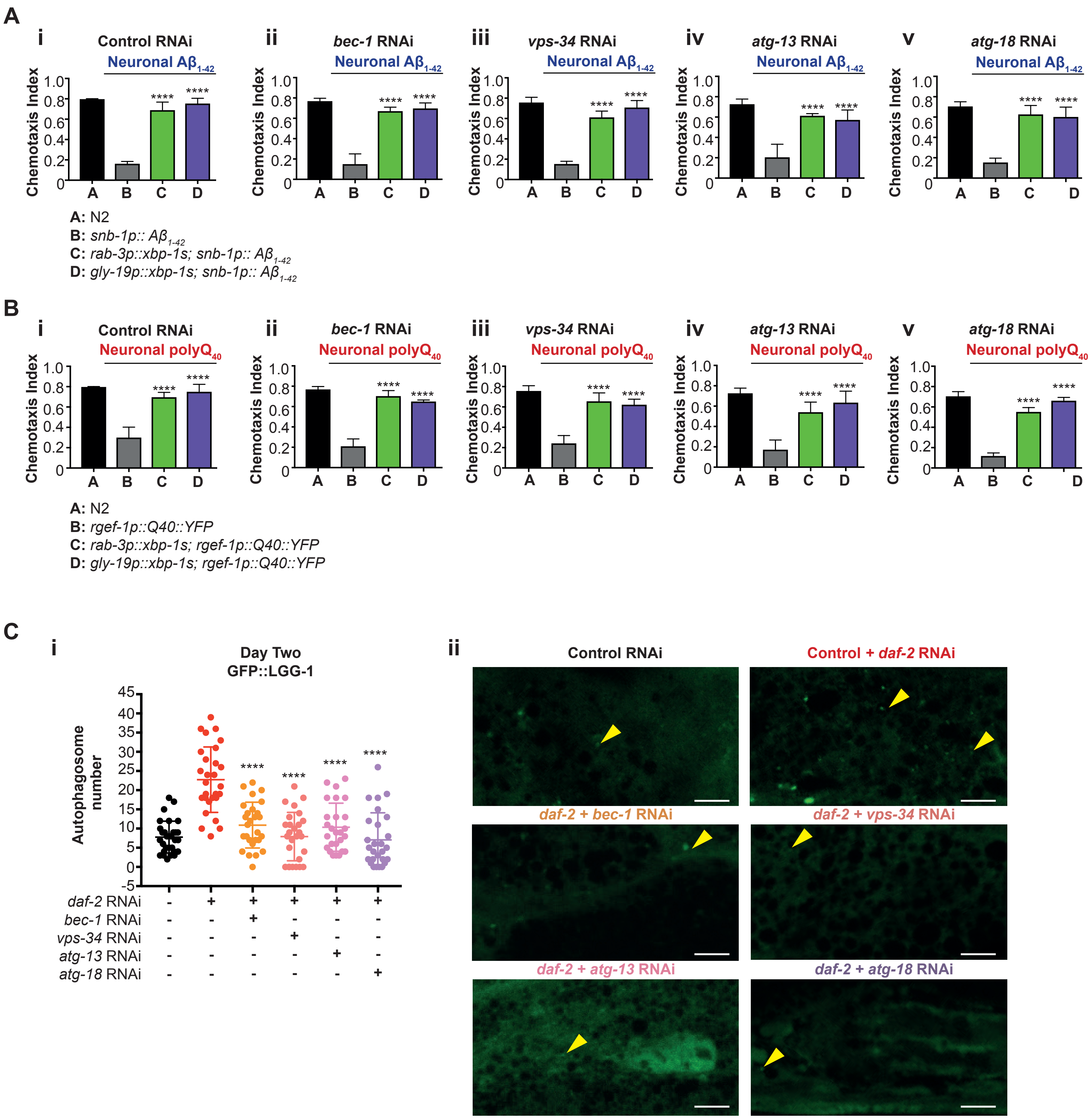
A
i Control RNAi
Neuronal Aβ1-42
ii bec-1 RNAi
iii vps-34 RNAi
iv atg-13 RNAi
v atg-18 RNAi
Chemotaxis Index
A B C D
****
A: N2
B: snb-1p:: Aβ1-42
C: rab-3p::xbp-1s; snb-1p:: Aβ1-42
D: gly-19p::xbp-1s; snb-1p:: Aβ1-42
B
i Control RNAi
Neuronal polyQ40
ii bec-1 RNAi
iii vps-34 RNAi
iv atg-13 RNAi
v atg-18 RNAi
A: N2
B: rgef-1p::Q40::YFP
C: rab-3p::xbp-1s; rgef-1p::Q40::YFP
D: gly-19p::xbp-1s; rgef-1p::Q40::YFP
C
i Day Two
GFP::LGG-1
Autophagosome number
daf-2 RNAi
bec-1 RNAi
vps-34 RNAi
atg-13 RNAi
atg-18 RNAi
ii Control RNAi
Control + daf-2 RNAi
daf-2 + bec-1 RNAi
daf-2 + vps-34 RNAi
daf-2 + atg-13 RNAi
daf-2 + atg-18 RNAi

Figure S4. Protection from proteotoxicity in *xbp-1s*-expressing animals does not depend upon genes involved in autophagy. Related to Figure 4.

(A) Chemotaxis ability in animals expressing $A\beta_{1-42}$ in neurons in combination with neuronal and intestinal *xbp-1s*, grown on (i) control (empty vector), (ii) *bec-1*, (iii) *vps-34*, (iv) *atg-13*, or (v) *atg-18* RNAi. Graphs represent mean chemotaxis index ± SD. N = 80-150 animals per assay, and each assay was independently replicated 3 times. Significance between neuronal $A\beta_{1-42}$ (B) and *xbp-1s*-expressing (C/D) strains was assessed by two-way ANOVA with Dunnett's multiple comparisons test, ****p<0.0001.

(B) Chemotaxis ability in animals expressing $polyQ_{40}$ in neurons in combination with neuronal and intestinal *xbp-1s*, grown on (i) control (empty vector), (ii) *bec-1*, (iii) *vps-34*, (iv) *atg-13*, or (v) *atg-18* RNAi. Graphs represent mean chemotaxis index ± SD. N = 80-170 animals per assay, and each assay was independently replicated 3 times. Significance between neuronal $polyQ_{40}$ (B) and *xbp-1s*-expressing (C/D) strains was assessed by two-way ANOVA with Dunnett's multiple comparisons test, ****p<0.0001.

(C) (i) Quantification of autophagic vesicles. Animals were grown on control, control + *daf-2*, *daf-2* + *bec-1*, *daf-2* + *vps-34*, *daf-2* + *atg-13*, or *daf-2* + *atg-18* RNAi. GFP::LGG-1-positive punctae were imaged at X63 magnification and counted in 25-30 worms per genotype at day 2 of adulthood using ImageJ. Data are derived from 3 independent biological replicates. Statistical analysis was performed relative to control + *daf-2* RNAi, using one-way ANOVA with Tukey's multiple comparisons test, ****p<0.0001.

(ii) Representative confocal images of the intestine of animals expressing GFP::LGG-1, grown on control, control + *daf-2*, *daf-2* + *bec-1*, *daf-2* + *vps-34*,

daf-2 + *atg-13*, or *daf-2* + *atg-18* RNAi. Animals were imaged at day 2 of adulthood, at X63 magnification. Scale bar = 5 μm.

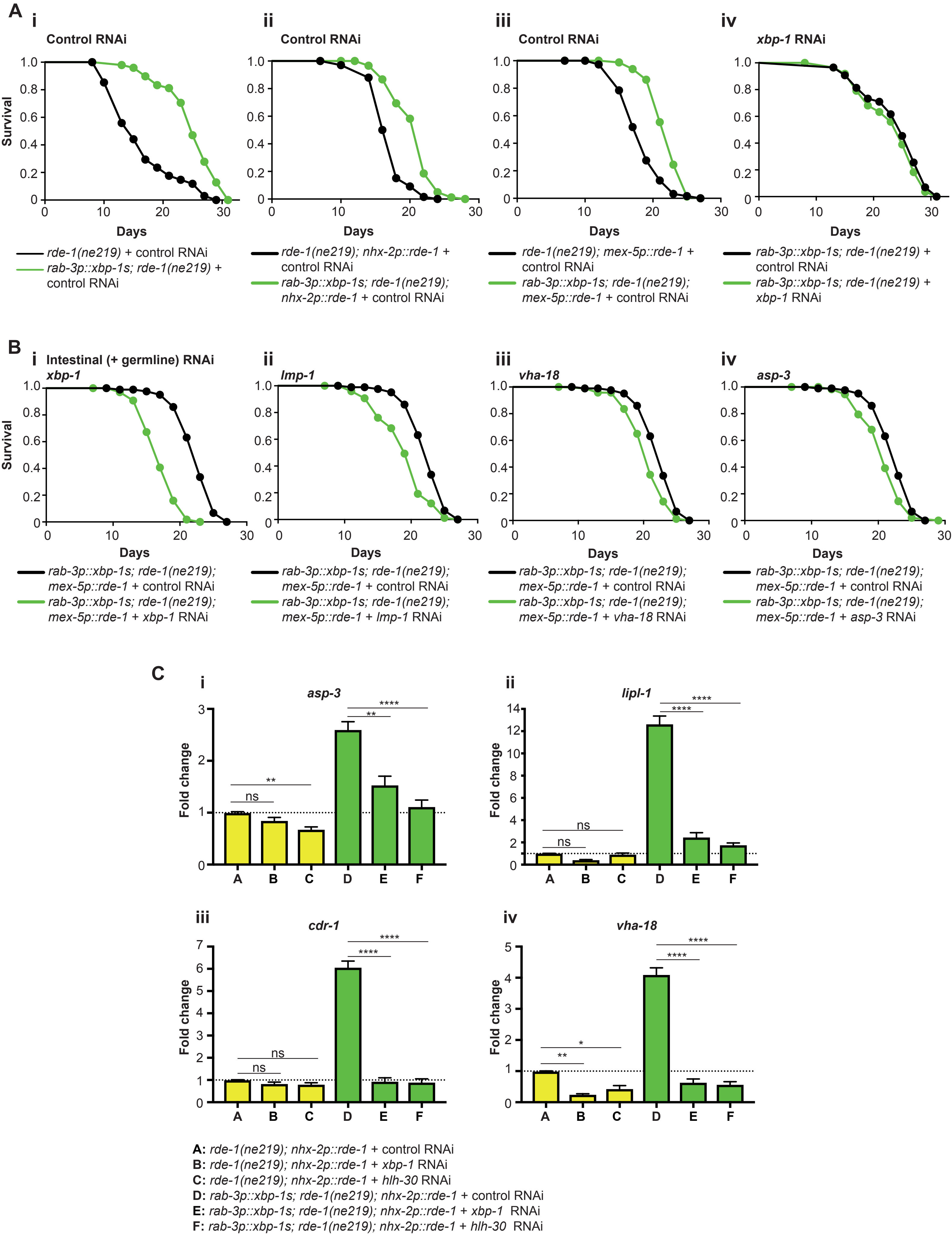
A
i
Control RNAi
Survival
Days
rde-1(ne219) + control RNAi
rab-3p::xbp-1s; rde-1(ne219) + control RNAi
ii
Control RNAi
Days
rde-1(ne219); nhx-2p::rde-1 + control RNAi
rab-3p::xbp-1s; rde-1(ne219); nhx-2p::rde-1 + control RNAi
iii
Control RNAi
Days
rde-1(ne219); mex-5p::rde-1 + control RNAi
rab-3p::xbp-1s; rde-1(ne219); mex-5p::rde-1 + control RNAi
iv
xbp-1 RNAi
Days
rab-3p::xbp-1s; rde-1(ne219) + control RNAi
rab-3p::xbp-1s; rde-1(ne219) + xbp-1 RNAi
B
i
Intestinal (+ germline) RNAi
xbp-1
Survival
Days
rab-3p::xbp-1s; rde-1(ne219); mex-5p::rde-1 + control RNAi
rab-3p::xbp-1s; rde-1(ne219); mex-5p::rde-1 + xbp-1 RNAi
ii
lmp-1
Days
rab-3p::xbp-1s; rde-1(ne219); mex-5p::rde-1 + control RNAi
rab-3p::xbp-1s; rde-1(ne219); mex-5p::rde-1 + lmp-1 RNAi
iii
vha-18
Days
rab-3p::xbp-1s; rde-1(ne219); mex-5p::rde-1 + control RNAi
rab-3p::xbp-1s; rde-1(ne219); mex-5p::rde-1 + vha-18 RNAi
iv
asp-3
Days
rab-3p::xbp-1s; rde-1(ne219); mex-5p::rde-1 + control RNAi
rab-3p::xbp-1s; rde-1(ne219); mex-5p::rde-1 + asp-3 RNAi
C
i
asp-3
Fold change
ii
lipl-1
Fold change
iii
cdr-1
Fold change
iv
vha-18
Fold change
A: rde-1(ne219); nhx-2p::rde-1 + control RNAi
B: rde-1(ne219); nhx-2p::rde-1 + xbp-1 RNAi
C: rde-1(ne219); nhx-2p::rde-1 + hlh-30 RNAi
D: rab-3p::xbp-1s; rde-1(ne219); nhx-2p::rde-1 + control RNAi
E: rab-3p::xbp-1s; rde-1(ne219); nhx-2p::rde-1 + xbp-1 RNAi
F: rab-3p::xbp-1s; rde-1(ne219); nhx-2p::rde-1 + hlh-30 RNAi

Figure S5. Validation of intestine-specific RNAi. Related to Figure 5 and Table S1.

(A) (i) Lifespan analysis of *rde(n219)* and *rab-3p::xbp-1s; rde(n219)* animals grown on control (empty vector) RNAi. *rde(n219)*, control (black), median lifespan 15 days; *rab-3p::xbp-1s; rde(n219)*, control (green), median lifespan 25 days, p<0.0001. Graph plotted as a Kaplan-Meier survival curve, P value calculated by Mantel-Cox log-rank test; N = 80-120 animals per lifespan.

(ii) Lifespan analysis of *rde(n219); nhx-2p::rde-1* and *rab-3p::xbp-1s; rde(n219); nhx-2p::rde-1* animals grown on control (empty vector) RNAi. *rde(n219); nhx-2p::rde-1*, control (black), median lifespan 18 days; *rab-3p::xbp-1s; rde(n219); nhx-2p::rde-1*, control (green), median lifespan 22 days, p<0.0001. Graph plotted as a Kaplan-Meier survival curve, P value calculated by Mantel-Cox log-rank test; N = 80-120 animals per lifespan.

(iii) Lifespan analysis of *rde(n219); mex-5p::rde-1* and *rab-3p::xbp-1s; rde(n219); mex-5p::rde-1* animals grown on control (empty vector) RNAi. *rde(n219); mex-5p::rde-1*, control (black), median lifespan 19 days; *rab-3p::xbp-1s; rde(n219); mex-5p::rde-1*, control (green), median lifespan 23 days, p<0.0001. Graph plotted as a Kaplan-Meier survival curve, P value calculated by Mantel-Cox log-rank test; N = 80-120 animals per lifespan.

(iv) Lifespan analysis of *rab-3p::xbp-1s; rde(n219)* animals grown on control (empty vector) and *xbp-1* RNAi. *rab-3p::xbp-1s; rde(n219)*, control (black), median lifespan 25 days; *rab-3p::xbp-1s; rde(n219)*, *xbp-1* (green), median lifespan 25 days, p=0.2281. Graph plotted as a Kaplan-Meier survival curve, P value calculated by Mantel-Cox log-rank test; N = 80-120 animals per lifespan.

(B) Lifespan analysis of *rab-3p::xbp-1s; rde(n219); mex-5p::rde-1* animals grown on control (empty vector) or (i) *xbp-1*, (ii) *lmp-1*, (iii) *vha-18*, or (iv) *asp-3* RNAi. Graphs were plotted as Kaplan-Meier survival curves, P values were calculated by Mantel-Cox log-rank test.

(i) *rab-3p::xbp-1s; rde(n219); mex-5p::rde-1*, control (black), median lifespan 23 days; *rab-3p::xbp-1s; rde(n219); mex-5p::rde-1*, *xbp-1* (green), median lifespan 17 days, p<0.0001.

(ii) *rab-3p::xbp-1s; rde(n219); mex-5p::rde-1*, control (black), median lifespan 23 days; *rab-3p::xbp-1s; rde(n219); mex-5p::rde-1*, *lmp-1* (green), median lifespan 19 days, p<0.0001.

(iii) *rab-3p::xbp-1s; rde(n219); mex-5p::rde-1*, control (black), median lifespan 23 days; *rab-3p::xbp-1s; rde(n219); mex-5p::rde-1*, *vha-18* (green), median lifespan 21 days, p<0.0001.

(iv) *rab-3p::xbp-1s; rde(n219); mex-5p::rde-1*, control (black), median lifespan 23 days; *rab-3p::xbp-1s; rde(n219); mex-5p::rde-1*, *asp-3* (green), median lifespan 21 days, p<0.01.

(C) qPCR of (i) *asp-3*, (ii) *lipl-1*, (iii) *cdr-1* and (iv) *vha-18* transcript levels at day 1 of adulthood in *rde-1(ne219); nhx-2p::rde-1* animals, with and without *rab-3p::xbp-1s* expression, grown on control, *xbp-1* or *hlh-30* RNAi. Expression was normalized to *rde-1(ne219); nhx-2p::rde-1* on control RNAi and significance measured by one-way ANOVA with Tukey's multiple comparisons test, ns = not significant, *p<0.05, **p<0.01, ****p<0.0001.

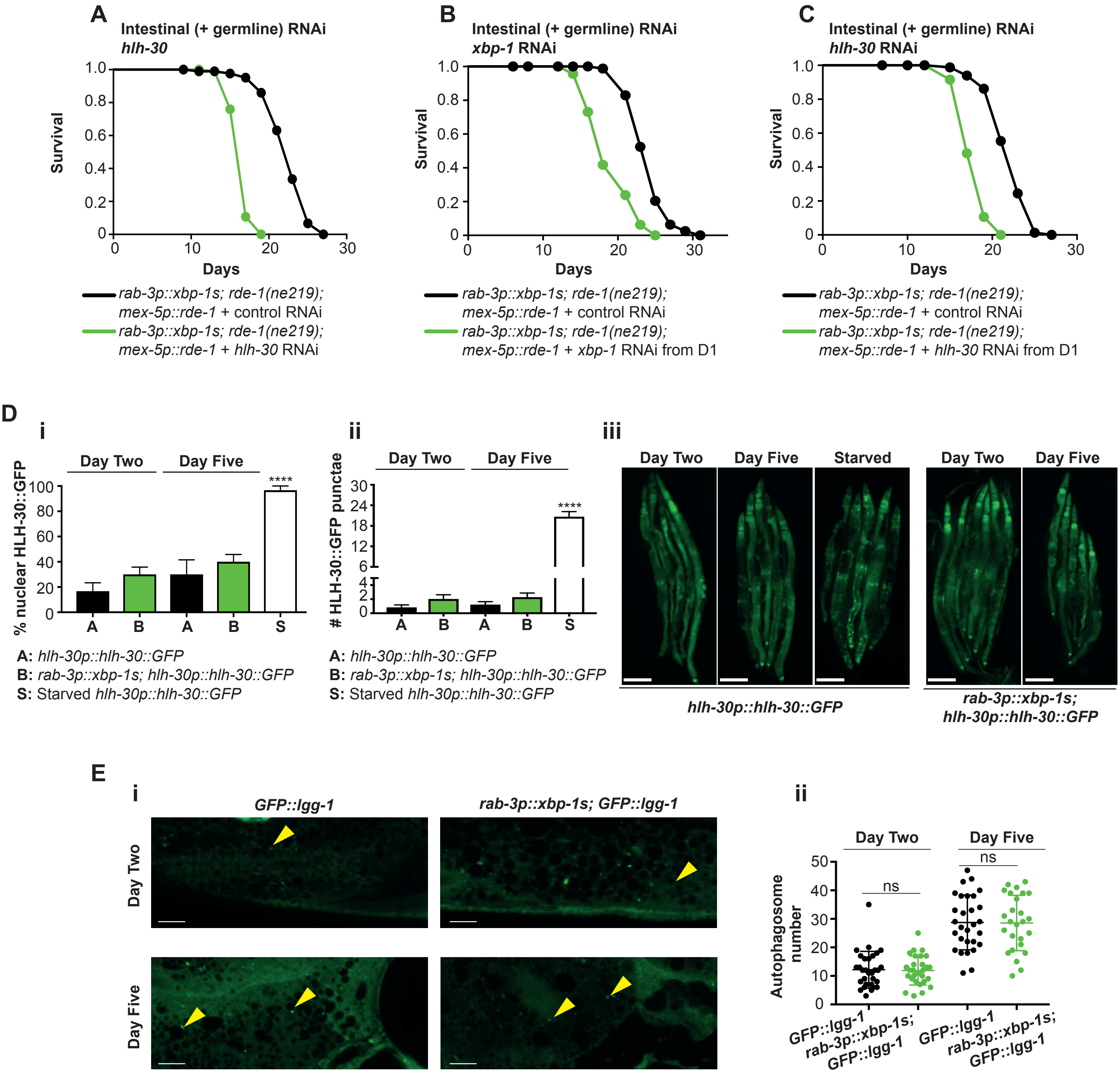
A
Intestinal (+ germline) RNAi
hlh-30
Survival
Days
rab-3p::xbp-1s; rde-1(ne219); mex-5p::rde-1 + control RNAi
rab-3p::xbp-1s; rde-1(ne219); mex-5p::rde-1 + hlh-30 RNAi
B
Intestinal (+ germline) RNAi
xbp-1 RNAi
rab-3p::xbp-1s; rde-1(ne219); mex-5p::rde-1 + control RNAi
rab-3p::xbp-1s; rde-1(ne219); mex-5p::rde-1 + xbp-1 RNAi from D1
C
Intestinal (+ germline) RNAi
hlh-30 RNAi
rab-3p::xbp-1s; rde-1(ne219); mex-5p::rde-1 + control RNAi
rab-3p::xbp-1s; rde-1(ne219); mex-5p::rde-1 + hlh-30 RNAi from D1
D
i
Day Two
Day Five
% nuclear HLH-30::GFP
****
A: hlh-30p::hlh-30::GFP
B: rab-3p::xbp-1s; hlh-30p::hlh-30::GFP
S: Starved hlh-30p::hlh-30::GFP
ii
HLH-30::GFP punctae
iii
Day Two
Day Five
Starved
hlh-30p::hlh-30::GFP
rab-3p::xbp-1s; hlh-30p::hlh-30::GFP
E
i
GFP::lgg-1
rab-3p::xbp-1s; GFP::lgg-1
Day Two
Day Five
ii
Autophagosome number
ns
GFP::lgg-1
rab-3p::xbp-1s; GFP::lgg-1

Figure S6. Analysis of HLH-30 and LGG-1 localization in intestinal cells. Related to Figures 5 and 6 and Table S1.

(A) Lifespan analysis of *rab-3p::xbp-1s; rde(n219); mex-5p::rde-1* animals grown on control (empty vector) or *hlh-30* RNAi. *rab-3p::xbp-1s; rde(n219); mex-5p::rde-1*, control (black), median lifespan 23 days; *rab-3p::xbp-1s; rde(n219); mex-5p::rde-1*, *hlh-30* (green), median lifespan 17 days, $p<0.0001$. Graphs were plotted as Kaplan-Meier survival curves, P values were calculated by Mantel-Cox log-rank test.

(B) Lifespan analysis of *rab-3p::xbp-1s; rde(n219); mex-5p::rde-1* animals grown on control (empty vector) RNAi throughout life, or on control (empty vector) RNAi until day 1 of adulthood and transferred to *xbp-1* RNAi from this age. *rab-3p::xbp-1s; rde(n219); mex-5p::rde-1*, control (black), median lifespan 25 days; *rab-3p::xbp-1s; rde(n219); mex-5p::rde-1*, *xbp-1* from adulthood (green), median lifespan 18 days, $p<0.0001$. Graph plotted as a Kaplan-Meier survival curve, P value calculated by Mantel-Cox log-rank test; N = 80-120 animals per lifespan.

(C) Lifespan analysis of *rab-3p::xbp-1s; rde(n219); mex-5p::rde-1* animals grown on control (empty vector) RNAi throughout life, or on control (empty vector) RNAi until day 1 of adulthood and transferred to *hlh-30* RNAi from this age. *rab-3p::xbp-1s; rde(n219); mex-5p::rde-1*, control (black), median lifespan 23 days; *rab-3p::xbp-1s; rde(n219); mex-5p::rde-1*, *hlh-30* from adulthood (green), median lifespan 17 days, $p<0.0001$. Graph plotted as a Kaplan-Meier survival curve, P value calculated by Mantel-Cox log-rank test; N = 80-120 animals per lifespan.

(D) (i) Quantification of HLH-30::GFP nuclear localization. Numbers of animals with nuclear HLH-30::GFP in the intestine were quantified at day 2 and day 5 of adulthood (except starved animals which were quantified at day 2 only); graphs represent mean percentage of animals with nuclear HLH-30::GFP ± SEM. N = 30 animals per assay, independently replicated 3 times. Significance was assessed by one-way ANOVA with Tukey's multiple comparisons test, ****p<0.0001.

(ii) Quantification of HLH-30::GFP-positive nuclei per animal. Numbers of HLH-30::GFP-positive nuclei in the intestine were quantified per animal at day 2 and day 5 of adulthood (except starved animals which were quantified at day 2 only); graphs represent mean percentage of animals with nuclear HLH-30::GFP ± SEM. N = 30 animals per assay, independently replicated 3 times. Significance was assessed by one way ANOVA with Tukey's multiple comparisons test, ****p<0.0001.

(iii) Confocal images of animals expressing HLH-30::GFP with and without *rab-3p::xbp-1s* at day 2 and day 5 of adulthood, alongside starved animals expressing HLH-30::GFP at day 2. Scale bar = 100 μm.

(E) (i) Representative confocal images of the intestine of animals expressing GFP::LGG-1, with and without *rab-3p::xbp-1s*. Animals were grown on OP50 and imaged at days 2 and 5 of adulthood. Imaging was performed at X63 magnification. Arrowheads indicate representative autophagosomes. Scale bar = 5 μm.

(ii) Quantification of autophagic vesicles. GFP::LGG-1-positive punctae were imaged at X63 magnification and counted in 10-15 worms per genotype at day 2 and day 5 of adulthood using ImageJ. Data are derived from 3

independent biological replicates. Statistical analysis was performed using one-way ANOVA with Tukey's multiple comparisons test. ns = not significant.

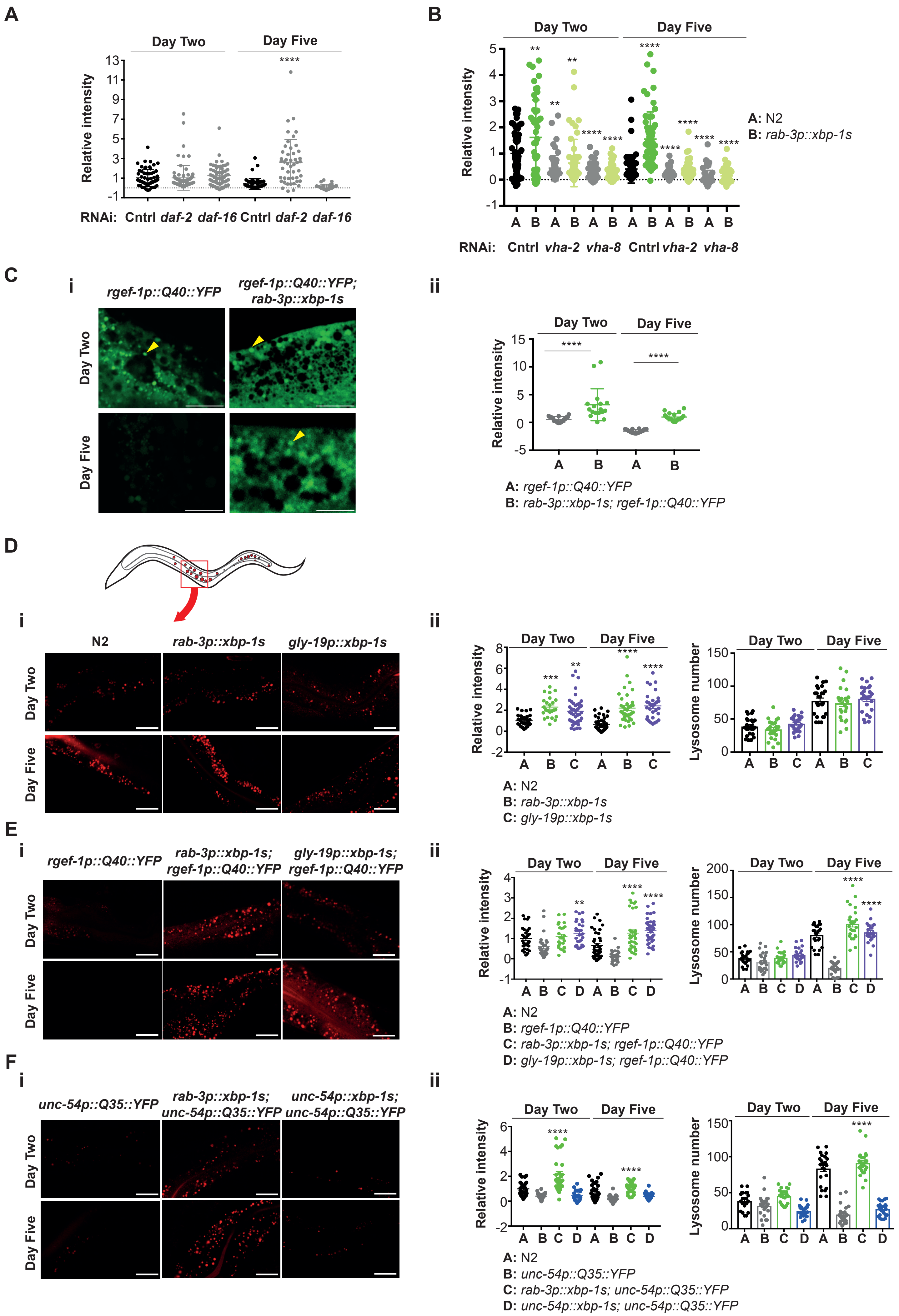
A
Day Two
Day Five
Relative intensity
****
RNAi: Cntrl daf-2 daf-16 Cntrl daf-2 daf-16
B
Day Two
Day Five
Relative intensity
A: N2
B: rab-3p::xbp-1s
RNAi: Cntrl vha-2 vha-8 Cntrl vha-2 vha-8
C
i
rgef-1p::Q40::YFP
rgef-1p::Q40::YFP; rab-3p::xbp-1s
Day Two
Day Five
ii
A: rgef-1p::Q40::YFP
B: rab-3p::xbp-1s; rgef-1p::Q40::YFP
D
i
N2
rab-3p::xbp-1s
gly-19p::xbp-1s
ii
Lysosome number
A: N2
B: rab-3p::xbp-1s
C: gly-19p::xbp-1s
E
i
rgef-1p::Q40::YFP
rab-3p::xbp-1s; rgef-1p::Q40::YFP
gly-19p::xbp-1s; rgef-1p::Q40::YFP
ii
A: N2
B: rgef-1p::Q40::YFP
C: rab-3p::xbp-1s; rgef-1p::Q40::YFP
D: gly-19p::xbp-1s; rgef-1p::Q40::YFP
F
i
unc-54p::Q35::YFP
rab-3p::xbp-1s; unc-54p::Q35::YFP
unc-54p::xbp-1s; unc-54p::Q35::YFP
ii
A: N2
B: unc-54p::Q35::YFP
C: rab-3p::xbp-1s; unc-54p::Q35::YFP
D: unc-54p::xbp-1s; unc-54p::Q35::YFP

Figure S7. Validation of lysosomal acidity staining and analysis of lysosomal acidity in animals expressing polyQ expansions. Related to Figure 7.

(A) Quantification of lysosomal acidity in the intestine of animals grown on control (empty vector), *daf-2* or *daf-16* RNAi. Animals were transferred to plates containing cDCFDA 16 hours prior to imaging. Using X63 magnification, imaging was conducted at day 2 and day 5 of adulthood. Quantification of fluorescence from 3 experiments was carried out using ImageJ. Plots represent 10 animals per replicate. Significance was assessed at each time point by an ordinary one-way ANOVA with Tukey's multiple comparisons, ****$p<0.0001$.

(B) Quantification of lysosomal acidity in the intestine of N2 and *rab-3p::xbp-1s*–expressing animals grown on control, *vha-2* or *vha-8* RNAi. Animals were transferred to plates containing cDCFDA 16 hours prior to imaging. Using X63 magnification, imaging was conducted at day 2 and day 5 of adulthood. Quantification of fluorescence from 3 experiments was carried out using ImageJ. Plots represent 10 animals per replicate. Significance was assessed in each genotype relative to control RNAi at both time points by ordinary one-way ANOVA with Tukey's multiple comparisons, **$p<0.01$, ****$p<0.0001$.

(C) (i) Confocal imaging of acidic lysosomes in the intestine of neuronal polyQ$_{40}$ animals, with and without neuronal *xbp-1s*. Animals were grown on OP50 and transferred to plates containing cDCFDA 16 hours prior to imaging. Using X63 magnification, imaging was conducted at day 2 and day 5 of adulthood. Scale bar = 10 μm.

(ii) Quantification of lysosomal acidity in the intestine of neuronal polyQ$_{40}$ animals, with and without neuronal *xbp-1s*. Animals were grown on OP50 and transferred to plates containing cDCFDA 16 hours prior to imaging and imaged as (D); quantification of fluorescence from 2 experiments was carried out using ImageJ. Plots represent 5-10 animals per replicate. Significance was assessed between N2 and *rab-3p::xbp-1s* at each time point by one-way ANOVA with Tukey's multiple comparisons, ****p<0.0001.

(D) (i) Confocal imaging of N2, *rab-3p::xbp-1s* and *gly-19p::xbp-1s* nematodes stained with LysoTracker Deep Red at days 2 and 5 of adulthood. Scale bar = 20 µm.

(ii) Quantification of intensity and number of Lysotracker-stained punctae in animals imaged as above. ImageJ was used to assess fluorescence intensity, which was normalized to N2. Each plot represents mean ± SEM from 3 independent biological replicates, N = 8-10 animals per replicate. Significance was calculated using one way ANOVA with Tukey's multiple comparisons test, **p<0.01, ***p<0.001, ****p<0.0001.

(E) (i) Confocal imaging of *rgef-1p::Q40::YFP* animals with or without *rab-3p::xbp-1s* and *gly-19p::xbp-1s,* stained with LysoTracker Deep Red at days 2 and 5 of adulthood. Scale bar = 20 µm.

(ii) Quantification of intensity and number of Lysotracker-stained punctae in *rgef-1p::Q40::YFP* animals imaged as above. ImageJ was used to assess fluorescence intensity, which was normalized to N2. Each plot represents mean ± SEM from 3 independent biological replicates, N = 8-10 animals per replicate. Significance was calculated using one way ANOVA with Tukey's multiple comparisons test, **p<0.01, ****p<0.0001.

(F) (i) Confocal imaging of *unc-54p::Q35::YFP* animals with or without *rab-3p::xbp-1s* and *unc-54p::xbp-1s,* stained with LysoTracker Deep Red at days 2 and 5 of adulthood. Scale bar = 20 μm.

(ii) Quantification of intensity and number of Lysotracker-stained punctae in *unc-54p::Q35::YFP* animals imaged as above. ImageJ was used to assess fluorescence intensity, which was normalized to N2. Each plot represents mean ± SEM from 3 independent biological replicates, N = 8-10 animals per replicate. Significance was calculated using one way ANOVA with Tukey's multiple comparisons test, ****p<0.0001.
