# Supplementary material for: Neuronal XBP-1 Activates Intestinal Lysosomes to Improve Proteostasis in C. elegans
Source: Curr Biol. 2019 Jul 22;29(14):2322–2338.e7. doi: 10.1016/j.cub.2019.06.031 (PMC6658570; doi:10.1016/j.cub.2019.06.031)
Supplement: Document S2. Article plus Supplemental Information [file mmc5.pdf]

Current Biology

Article

Neuronal XBP-1 Activates Intestinal Lysosomes to Improve Proteostasis in *C. elegans*

Graphical Abstract

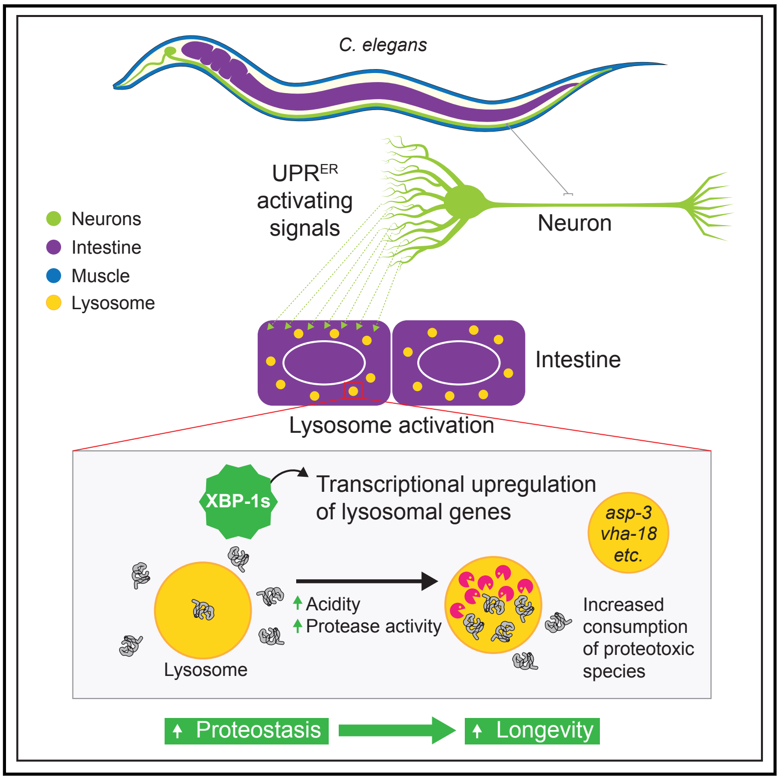

Highlights

- *Xbp-1s* expressed in the neurons or intestine of *C. elegans* improves proteostasis
- Neuronal *xbp-1s* drives expression of lysosomal genes in the intestine
- Intestinal lysosomes show enhanced acidity and activity upon *xbp-1s* expression
- Lysosome function is required for *xbp-1s* to increase proteostasis and longevity

Authors

Soudabeh Imanikia, Neşem P. Özbey, Christel Krueger, M. Olivia Casanueva, Rebecca C. Taylor

Correspondence

rtaylor@mrc-lmb.cam.ac.uk

In Brief

Expressing the UPR^{ER} transcription factor *xbp-1s* in the neurons or intestine of *C. elegans* extends lifespan. Imanikia et al. show that neuronal *xbp-1s* upregulates lysosomal genes and activates lysosomes in the intestine and that intestinal lysosome function is crucial for *xbp-1s*-mediated increases in proteostasis and longevity.

Imanikia et al., 2019, Current Biology *29*, 2322–2338
July 22, 2019 © 2019 MRC Laboratory of Molecular Biology. Published by Elsevier Ltd.
https://doi.org/10.1016/j.cub.2019.06.031

Current Biology
Article

Neuronal XBP-1 Activates Intestinal Lysosomes to Improve Proteostasis in *C. elegans*

Soudabeh Imanikia,[1] Neşem P. Özbey,[1] Christel Krueger,[2] M. Olivia Casanueva,[2] and Rebecca C. Taylor[1,3,*]
[1]Neurobiology Division, MRC Laboratory of Molecular Biology, Cambridge CB2 0QH, UK
[2]Epigenetics Programme, The Babraham Institute, Babraham CB22 3AT, UK
[3]Lead Contact
*Correspondence: rtaylor@mrc-lmb.cam.ac.uk
https://doi.org/10.1016/j.cub.2019.06.031

SUMMARY

The unfolded protein response of the endoplasmic reticulum (UPR^{ER}) is a crucial mediator of secretory pathway homeostasis. Expression of the spliced and active form of the UPR^{ER} transcription factor XBP-1, XBP-1s, in the nervous system triggers activation of the UPR^{ER} in the intestine of *Caenorhabditis elegans* (*C. elegans*) through release of a secreted signal, leading to increased longevity. We find that expression of XBP-1s in the neurons or intestine of the worm strikingly improves proteostasis in multiple tissues, through increased clearance of toxic proteins. To identify the mechanisms behind this enhanced proteostasis, we conducted intestine-specific RNA-seq analysis to identify genes upregulated in the intestine when XBP-1s is expressed in neurons. This revealed that neuronal XBP-1s increases the expression of genes involved in lysosome function. Lysosomes in the intestine of animals expressing neuronal XBP-1s are more acidic, and lysosomal protease activity is higher. Moreover, intestinal lysosome function is necessary for enhanced lifespan and proteostasis. These findings suggest that activation of the UPR^{ER} in the intestine through neuronal signaling can increase the activity of lysosomes, leading to extended longevity and improved proteostasis across tissues.

INTRODUCTION

Aging is a process that occurs in a coordinated fashion throughout the body, leading to a susceptibility to deterioration and disease across tissues. The choreography of the aging process is achieved through inter-tissue signaling, often involving the nervous system. In *C. elegans*, mutations in genes that affect nervous system function can significantly increase longevity [1]. This effect is also observed in other species—in *Drosophila melanogaster*, sensory perception of food can alter lifespan in a similar way to consumption of that food, while, in mice, loss of the neuronal pain receptor TRPV1 creates animals that live substantially longer, with improved metabolic profiles [2, 3]. The ability of neurons to modulate aging is therefore a conserved phenomenon. However, it is not clear whether these neuronal signals converge upon the same core mechanisms in distal tissues. Understanding these mechanisms could allow them to be engaged directly in order to improve aging phenotypes and increase cellular health.

Recently, the transmission of cellular stress response activation has been identified as a mechanism utilized by the nervous system to increase longevity [4]. Stress responses are organelle-specific pathways that connect the sensing of molecular damage and imbalances in homeostasis to mechanisms that restore equilibrium. They can be activated in distal tissues by signals secreted from neurons, typically leading to marked lifespan extension. One of these stress responses, the endoplasmic reticulum unfolded protein response (UPR^{ER}), is an adaptive signaling pathway that restores equilibrium within the secretory pathway. Its three branches are defined by their proximal sensor molecules, IRE1, PERK, and ATF6, which regulate downstream mechanisms that include both transcriptional and translational targets [5]. Activation of IRE1 leads to the regulated splicing and translation of the transcription factor XBP1. In *C. elegans*, expression of the spliced and active form of XBP-1, XBP-1s, in the nervous system triggers the release of an unidentified signal from neurons that activates this branch of the UPR^{ER} in the animal's intestine [6]. This signaling extends lifespan and increases stress resistance, with signal release dependent upon UNC-13, a neuron-specific regulator of synaptic secretion. The effects of UPR^{ER} activation are tissue specific—activation in the intestine, either directly or through neuronal signaling, is beneficial, while expression of XBP-1s in body wall muscle cells is detrimental to longevity. While the downstream targets and mechanisms invoked in distal tissues by XBP-1s to achieve changes in longevity are unknown, it is possible that tissue-specific differences in these XBP-1s targets lead to differing effects on lifespan.

One hallmark of aging is a loss of cellular homeostasis, particularly protein homeostasis (proteostasis) [7–9]. Loss of proteostasis underlies the onset of many diseases of aging, including neurodegenerative diseases, which are often caused by age-associated accumulation of toxic, aggregated proteins. Improving cellular proteostasis extends lifespan, and neurons are able to enhance proteostasis in distal tissues [10–12]. As stress responses can trigger mechanisms that restore proteostasis, one function of the distal activation of these pathways by neurons might be to improve proteostasis in other tissues, counteracting the loss of equilibrium associated with aging and leading to increased longevity.

 © 2019 MRC Laboratory of Molecular Biology. Published by Elsevier Ltd.
This is an open access article under the CC BY license (http://creativecommons.org/licenses/by/4.0/).

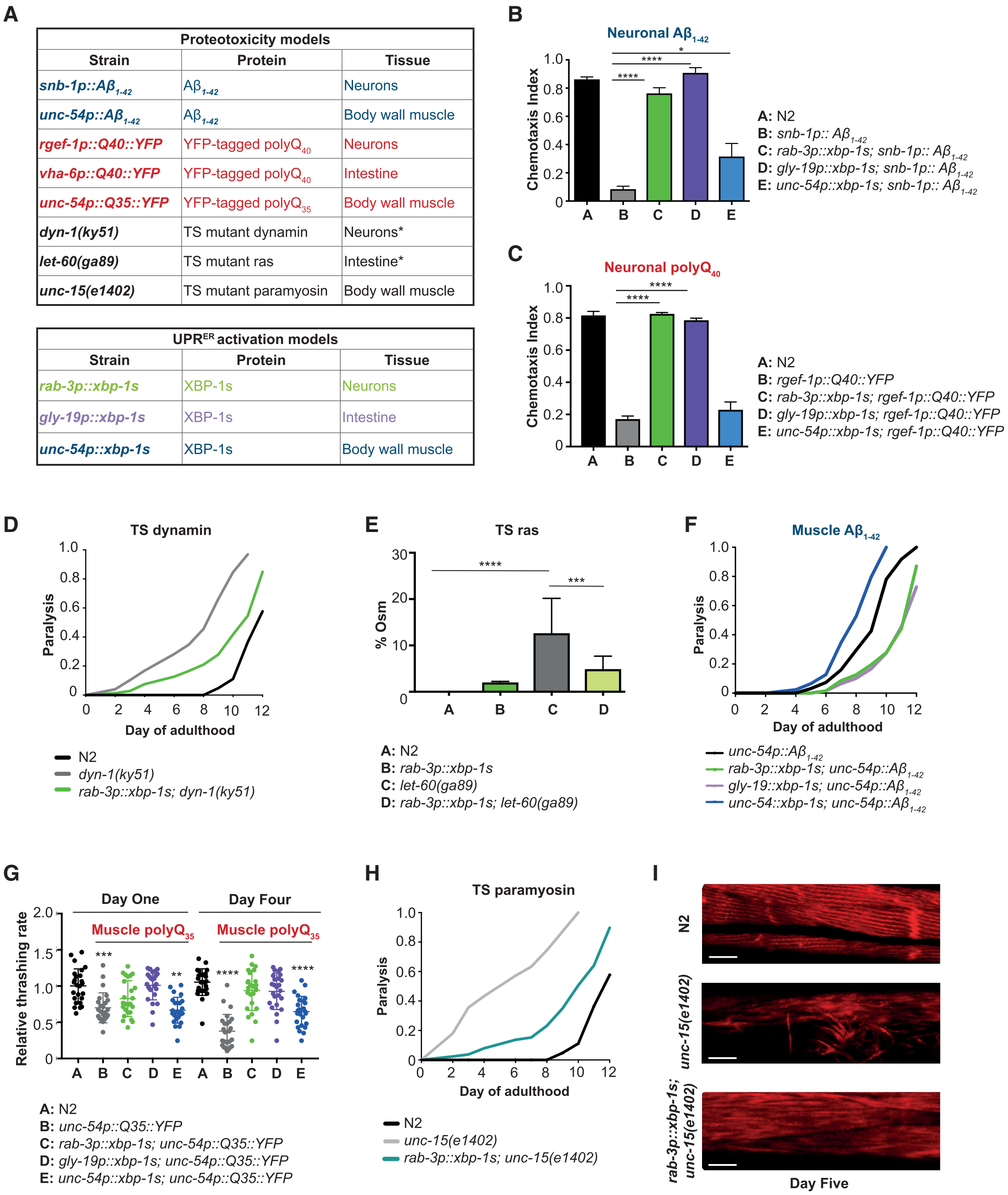

Figure 1. Expression of *xbp-1s* in Neurons or the Intestine Restores Proteostasis in Models of Proteotoxicity

(A) Proteotoxicity and UPR^{ER} models used in this study. TS, temperature sensitive. **dyn-1(ky51)* and *let-60(ga89)* are expressed in multiple tissues, but their functions in motor neurons and intestine, respectively, are assayed here.

(B) Chemotaxis ability in animals expressing Aβ_{1-42} in neurons, with and without neuronal, intestinal, or muscle *xbp-1s*. Graphs represent mean chemotaxis index ± SD. N = 80–150 animals per assay; each assay was independently replicated 3 times. Significance was assessed by one-way ANOVA with Tukey's multiple comparisons test, *$p < 0.05$, ****$p < 0.0001$.

(C) Chemotaxis ability in animals expressing polyQ$_{40}$::YFP in neurons, with and without neuronal, intestinal, or muscle *xbp-1s*. Graphs represent mean chemotaxis index ± SD. N = 80–150 animals per assay; each assay was independently replicated 3 times. Significance was assessed by one-way ANOVA with Tukey's multiple comparisons test, ****$p < 0.0001$.

(D) Paralysis in *dyn-1(ky51)* animals at 20°C, with and without neuronal *xbp-1s*. Animals exhibiting body paralysis were counted daily and the paralyzed fraction of the population plotted against time. N = 100 worms per assay; each assay was repeated 3 times.

(E) Prevalence of osmoregulatory defects in *let-60(ga89)* and wild-type animals, with and without *rab-3p::xbp-1s*. Animals were placed in distilled water at day 2 of adulthood, and a swollen body after 5 min was scored as osmoregulation defective (Osm). Data represent mean ± SD of 20–25 animals per strain in 3 independent replicates. Significance assessed by two-way ANOVA with Tukey's multiple comparison, ***$p < 0.001$, ****$p < 0.0001$.

(legend continued on next page)

Here, we have explored the mechanisms by which neuronal XBP-1s extends lifespan in *C. elegans*. Speculating that improved proteostasis might underlie the beneficial effects of XBP-1s on longevity and stress resistance, we have established that neuronal and intestinal XBP-1s improve proteostasis in multiple tissues of the organism by increasing clearance of toxic proteins. To identify transcriptional targets that underlie these improvements in longevity and proteostasis, we conducted tissue-specific RNA sequencing (RNA-seq) analysis in the intestines of animals expressing neuronal XBP-1s and found a prominent upregulation of lysosomal genes. Intestinal lysosomes play a key role in the extension of longevity and improvements in proteostasis downstream of neuronal UPR^{ER} activation, and in animals expressing neuronal XBP-1s lysosomal acidity is increased, indicating an increase in activity. This suggests that activation of lysosomes in the intestine is a key downstream mechanism by which neuronal XBP-1s improves lifespan and proteostasis.

RESULTS

To investigate the mechanisms by which XBP-1s might extend longevity, we started by asking whether its expression could improve proteostasis, either cell-autonomously or cell non-autonomously. To do this, we drove expression of spliced *xbp-1s* in different tissues of the worm (neurons, intestine, and body wall muscle cells, in animals expressing endogenous *xbp-1*) with models of proteotoxicity, in which aggregation-prone proteins are also expressed in different tissues of the animal (Figure 1A). Proteotoxicity was then assessed using physiological readouts of tissue function.

Neuronal and Intestinal *xbp-1s* Protects against Proteotoxicity in Neuronal and Intestinal Cells

$A\beta_{1-42}$ is a proteotoxic peptide associated with the development of Alzheimer's disease. To determine whether *xbp-1s* affects phenotypes associated with proteotoxicity, pan-neuronal expression of $A\beta_{1-42}$, with a signal peptide directing it to the secretory pathway [13], was combined with tissue-specific expression of *xbp-1s* in neuronal, intestinal, and body wall muscle cells, and the function of neurons measured by chemotaxis—movement of worms toward an attractive volatile odorant. Strikingly, expression of *xbp-1s* in either neurons or the intestine fully rescued the loss of chemotaxis caused by $A\beta_{1-42}$ expression, while the effect of muscle cell *xbp-1s* expression was limited (Figure 1B).

To then ask whether neuronal or intestinal *xbp-1s* could also exert these protective effects against another toxic protein, tissue-specific *xbp-1s* was combined with pan-neuronal expression of polyglutamine (polyQ) expansions, associated with disorders including Huntington's disease and shown to be good sensors for protein folding homeostasis in the cytosol [8, 14]. Again, expression of *xbp-1s* in neuronal or intestinal, but not muscle cells, fully rescued animals from loss of neuronal function, demonstrating that this transcription factor can mitigate the effects of multiple proteotoxic species, including those directed to the secretory pathway as well as those expressed cytosolically (Figure 1C).

To determine whether *xbp-1s* can also improve phenotypes associated with the misfolding of endogenous proteins, we used *C. elegans* strains containing temperature-sensitive missense mutations. These metastable proteins are nonfunctional at 25°C but at lower temperatures show an accelerated age-associated loss of folding and function that correlates with age-related decline in endogenous stress responses [7, 15]. A metastable allele of *dyn-1*, encoding a dynamin GTPase, was examined, as well as a temperature-sensitive allele of *let-60*, encoding a Ras GTPase homolog. The effect of *dyn-1(ky51)* on motor neurons was measured by age-associated motor paralysis; in animals expressing *xbp-1s* in neurons, paralysis of *dyn-1(ky51)* animals was substantially delayed (Figure 1D). To determine the effect of *let-60(ga89)* on intestinal function, osmoregulatory capacity was assessed. *Xbp-1s* expression in neurons significantly decreased the percentage of animals showing defects in osmoregulation (Figure 1E). Neuronal *xbp-1s* therefore protects against dysfunction associated with endogenous metastable, as well as human disease-associated proteins, in neuronal and intestinal cells.

Neuronal and Intestinal *xbp-1s* Protects against Proteotoxicity in Muscle Cells

To determine the effects of *xbp-1s* on proteotoxicity in a tissue other than neurons or the intestine, $A\beta_{1-42}$ (targeted to the secretory pathway) was expressed in body wall muscle cells [16–18] in combination with tissue-specific *xbp-1s*. Muscle function was then assayed by measuring body wall muscle paralysis with age. Surprisingly, although $A\beta_{1-42}$ was expressed within muscle cells, *xbp-1s* delayed toxicity and paralysis when expressed in neurons and intestine, but not when expressed in muscle cells themselves (Figure 1F). Tissue-specific *xbp-1s* was then combined with cytosolic polyQ$_{35}$ expression, and muscle cell function measured by swimming rate at day 1 and day 4 of adulthood. Expression of polyQ$_{35}$ in muscles caused a significant decline in motility, which was rescued by *xbp-1s* expression in neurons or the intestine, but not in muscle cells (Figure 1G).

Finally, we examined the effect of neuronal *xbp-1s* expression on a temperature-sensitive metastable allele of the muscle-specific gene *unc-15*, encoding paramyosin. Upon *xbp-1s*

(F) Paralysis in animals expressing $A\beta_{1-42}$ in body wall muscle cells, with and without tissue-specific *xbp-1s*. Animals exhibiting body paralysis were counted daily and the paralyzed fraction plotted against time. N = 100 worms per assay; each assay was repeated 3 times.

(G) Motility in animals expressing polyQ$_{35}$::YFP in body wall muscle cells, with and without tissue-specific *xbp-1s*. Plots represent swimming rate, normalized to N2 day 1 ± SD. N = 30–40 animals per assay; each assay was independently replicated 3 times. Significance was assessed by one-way ANOVA with Tukey's multiple comparisons test, $^{**}p < 0.01$, $^{***}p < 0.001$, $^{****}p < 0.0001$.

(H) Paralysis in populations of *unc-15(e1402)* animals at 20°C, with and without neuronal *xbp-1s*. Animals exhibiting body paralysis were counted daily and the paralyzed fraction plotted against time. N = 100 worms per assay; each assay was repeated 3 times.

(I) Muscle fiber organization in wild-type and *unc-15(e1402)* animals grown at 20°C, with and without neuronal *xbp-1s*. Muscle cells were visualized using F-actin staining at day 5 of adulthood. Scale bar, 10 μm.

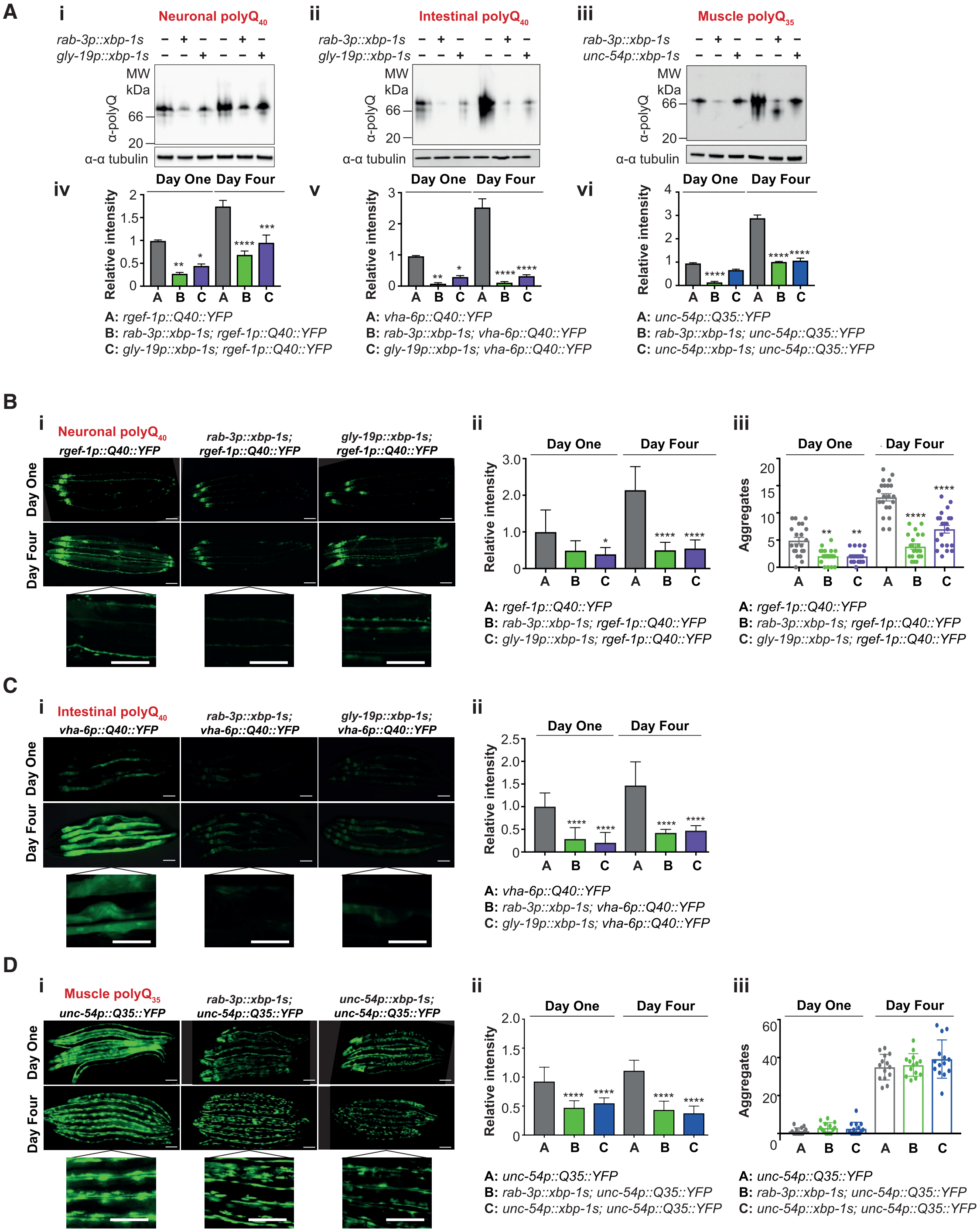

Figure 2. *Xbp-1s* Expression Reduces Levels of PolyQ

(A) Native western blot analysis of (i) neuronal polyQ$_{40}$::YFP, (ii) intestinal polyQ$_{40}$::YFP, and (iii) muscle polyQ$_{35}$::YFP with and without tissue-specific *xbp-1s*, at day 1 and day 4 of adulthood. Lysates were resolved under native conditions and blotted with an anti-polyQ antibody; the predicted MW of monomeric polyQ$_{35–40}$::YFP is ~32 kDa. Tubulin levels were probed with α-α-tubulin as a loading control. Blots were quantified using ImageJ (iv–vi). Bar graphs represent mean band intensity relative to day 1 polyQ ± SD and statistical significance was calculated between A and B/C at each time point using two-way ANOVA with Tukey's multiple comparisons test, *$p < 0.05$, **$p < 0.01$, ***$p < 0.001$, ****$p < 0.0001$. Data are representative of at least 3 independent experiments.

(B) (i) Epifluorescence visualization of neuronal polyQ$_{40}$::YFP, with and without neuronal and intestinal *xbp-1s*, at day 1 and day 4 of adulthood. Enlarged panels are from day 4 animals. Scale bar, 250 μm.

(ii) Fluorescence intensity of neuronal polyQ$_{40}$::YFP quantified from 3 independent experiments using ImageJ and expressed as mean fluorescence intensity relative to day 1 polyQ$_{40}$ ± SEM. N = 25–30 animals. Significance was assessed between A and B/C at each time point using two-way ANOVA with Tukey's multiple comparisons test, *$p < 0.05$, ****$p < 0.0001$.

(legend continued on next page)

expression, age-associated paralysis caused by *unc-15(1402)* was substantially delayed (Figure 1H). In addition, muscle fiber disorganization in *unc-15(1402)* animals at day 5 of adulthood was largely rescued by neuronal *xbp-1s* (Figure 1I). Together, these results suggest that *xbp-1s* in neurons or the intestine exerts a protective effect against proteotoxicity, even when the toxic species is expressed in an entirely different tissue.

Expression of *xbp-1s* Reduces the Accumulation of Misfolded Proteins

To determine whether *xbp-1s* exerts its beneficial effects on proteotoxicity through changes in levels of toxic proteins, we co-expressed neuronal or intestinal *xbp-1s* with $A\beta_{1-42}$ in neurons or muscle cells. When $A\beta_{1-42}$ was expressed in neurons, *xbp-1s* in neuronal or intestinal cells marginally reduced levels of $A\beta_{1-42}$ at day 4 of adulthood (Figures S1Ai and S1Aiii). Upon expression of $A\beta_{1-42}$ in muscle cells, however, neuronal and intestinal *xbp-1s* strikingly reduced $A\beta_{1-42}$ levels at both day 1 and day 4 (Figures S1Aii and S1Aiv). Notably, multiple species likely to correspond to soluble oligomers of $A\beta_{1-42}$, and associated with $A\beta_{1-42}$ toxicity, were substantially reduced by *xbp-1s* expression.

We then used native PAGE of YFP-tagged polyQ expansions co-expressed with tissue-specific *xbp-1s* to assess polyQ::YFP levels. This revealed that polyQ::YFP expressed in neuronal or intestinal [19] cells was substantially reduced by expression of *xbp-1s* in neurons or the intestine (Figures 2Ai, 2Aii, 2Aiv, 2Av, S1Bi, S1Bii, S1Biv, and S1Bv). Muscle-specific $polyQ_{35}$::YFP levels were reduced by expression of neuronal *xbp-1s*, and to a lesser extent by *xbp-1s* in muscle cells (Figures 2Aiii, 2Avi, S1Biii, and S1Bvi). We also analyzed polyQ::YFP under denaturing conditions and found that total polyQ levels were reduced by *xbp-1s*, although less strikingly than when assessed by native PAGE (and in muscle-specific $polyQ_{35}$, not at all at day 4) (Figure S1C). Denaturing conditions can resolve both soluble and other forms of polyQ, including insoluble aggregated species, whereas native analysis only resolves soluble proteins; one possibility is that *xbp-1s* preferentially reduces levels of soluble, lower-molecular-weight polyQ, rather than aggregated forms.

Tissue-specific polyQ::YFP was then visualized by microscopy at days 1 and 4 of adulthood, with and without tissue-specific *xbp-1s*. Neuronal $polyQ_{40}$::YFP fluorescence intensity increased between day 1 and day 4 but was reduced at both ages by neuronal or intestinal *xbp-1s* expression, especially by day 4 (Figures 2Bi and 2Bii). Numbers of neuronal $polyQ_{40}$::YFP aggregates were also reduced by neuronal or intestinal *xbp-1s* (Figure 2Biii). Intestinal $polyQ_{40}$::YFP fluorescence also increased with age, although aggregation was not observed, and was very substantially decreased by neuronal or intestinal *xbp-1s* expression at both day 1 and day 4 (Figures 2Ci and 2Cii). Muscle-specific $polyQ_{35}$::YFP was co-expressed with neuronal and muscle-specific *xbp-1s*, at both day 1 and day 4, expression of *xbp-1s* decreased $polyQ_{35}$::YFP fluorescence (Figures 2Di and 2Dii). However, $polyQ_{35}$::YFP aggregate accumulation in muscles was unaffected by *xbp-1s* (Figure 2Diii). Loss of $polyQ_{35}$::YFP fluorescence, but not aggregation, suggests again that XBP-1s may preferentially reduce levels of more soluble, lower-molecular-weight forms of $polyQ_{35}$. As animals are healthier (Figure 1G) despite having similar numbers of visible aggregates, we suggest that XBP-1s may reduce proteotoxicity primarily due to reduction in soluble proteotoxic species.

Neuronal and Intestinal *xbp-1s* Does Not Change $A\beta_{1-42}$ or PolyQ Transcript Levels

Importantly, no reduction in polyQ::YFP or $A\beta_{1-42}$ transcript levels were observed upon *xbp-1s* expression, confirming that changes in protein abundance are unlikely to arise from changes to transcription of these transgenes (Figures S2A and S2B). Although we cannot rule out changes to rates of translation, we hypothesize that reduced levels of proteotoxic species upon neuronal and intestinal *xbp-1s* expression may potentially be the result of increased misfolded protein clearance driven by *xbp-1s*.

Intestinal Genes Regulated by Neuronal *xbp-1s* Include Genes Involved in Lysosome Function

Given the profound effects of *xbp-1s* expression on proteotoxicity, we decided to explore the mechanisms by which *xbp-1s* might improve proteostasis. Neuronal and intestinal *xbp-1s* both protected against proteotoxic species in multiple tissues, while muscle-specific *xbp-1s* had only minor protective effects. Interestingly, *xbp-1s* expression in neurons and the intestine, but not muscle cells, extends longevity in *C. elegans* [6]. Neuronal *xbp-1s* expression leads to the communication of UPR^{ER} activation to intestinal cells, through the release of a signal secreted by neurons. We therefore hypothesized that the intestine may be a key tissue through which *xbp-1s* activity coordinates organism-wide improvements in lifespan and proteostasis.

(iii) Neuronal $polyQ_{40}$::YFP aggregates were counted in 15–20 animals per strain at day 1 and day 4 of adulthood and expressed as mean aggregates per animal ± SEM. Significance was assessed between A and B/C at each time point using two-way ANOVA with Tukey's multiple comparisons test, $^{**}p < 0.01$, $^{****}p < 0.0001$.
(C) (i) Epifluorescence visualization of intestinal $polyQ_{40}$::YFP, with and without neuronal and intestinal *xbp-1s*, at day 1 and day 4 of adulthood. Enlarged panels are from day 4 animals. Scale bar, 250 μm.
(ii) Fluorescence intensity of intestinal $polyQ_{40}$::YFP quantified from 3 independent experiments using ImageJ and expressed as mean fluorescence intensity relative to day 1 $polyQ_{40}$ ± SEM. N = 25–30 animals. Significance was assessed between A and B/C at each time point using two-way ANOVA with Tukey's multiple comparisons test, $^{****}p < 0.0001$.
(D) (i) Epifluorescence visualization of body wall muscle $polyQ_{35}$::YFP, with and without neuronal and body wall muscle *xbp-1s*, at day 1 and day 4 of adulthood. Enlarged panels are from day 4 animals. Scale bar, 250 μm.
(ii) Fluorescence intensity of body wall muscle $polyQ_{35}$::YFP quantified from three independent experiments using ImageJ and expressed as mean fluorescence intensity relative to day 1 $polyQ_{40}$ ± SEM. N = 25–30 animals. Significance was assessed between A and B/C at each time point using two-way ANOVA with Tukey's multiple comparisons test, $^{****}p < 0.0001$.
(iii) Body wall muscle $polyQ_{35}$::YFP aggregates were counted in 15–20 animals per strain at day 1 and day 4 of adulthood and expressed as mean aggregates per animal ± SEM. Significance was assessed between A and B/C at each time point using two-way ANOVA with Tukey's multiple comparisons test. See also Figures S1 and S2.

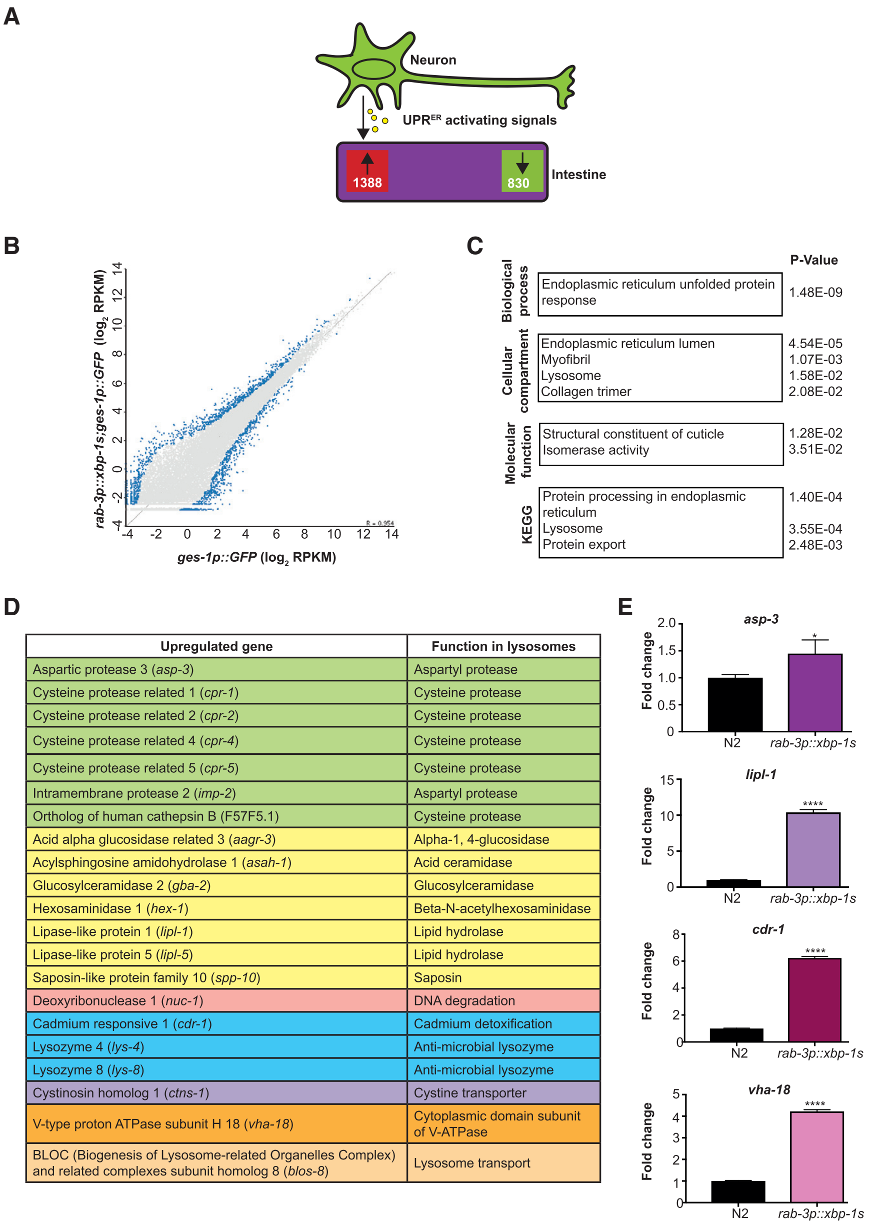

Upregulated gene	Function in lysosomes
Aspartic protease 3 (*asp-3*)	Aspartyl protease
Cysteine protease related 1 (*cpr-1*)	Cysteine protease
Cysteine protease related 2 (*cpr-2*)	Cysteine protease
Cysteine protease related 4 (*cpr-4*)	Cysteine protease
Cysteine protease related 5 (*cpr-5*)	Cysteine protease
Intramembrane protease 2 (*imp-2*)	Aspartyl protease
Ortholog of human cathepsin B (F57F5.1)	Cysteine protease
Acid alpha glucosidase related 3 (*aagr-3*)	Alpha-1, 4-glucosidase
Acylsphingosine amidohydrolase 1 (*asah-1*)	Acid ceramidase
Glucosylceramidase 2 (*gba-2*)	Glucosylceramidase
Hexosaminidase 1 (*hex-1*)	Beta-N-acetylhexosaminidase
Lipase-like protein 1 (*lipl-1*)	Lipid hydrolase
Lipase-like protein 5 (*lipl-5*)	Lipid hydrolase
Saposin-like protein family 10 (*spp-10*)	Saposin
Deoxyribonuclease 1 (*nuc-1*)	DNA degradation
Cadmium responsive 1 (*cdr-1*)	Cadmium detoxification
Lysozyme 4 (*lys-4*)	Anti-microbial lysozyme
Lysozyme 8 (*lys-8*)	Anti-microbial lysozyme
Cystinosin homolog 1 (*ctns-1*)	Cystine transporter
V-type proton ATPase subunit H 18 (*vha-18*)	Cytoplasmic domain subunit of V-ATPase
BLOC (Biogenesis of Lysosome-related Organelles Complex) and related complexes subunit homolog 8 (*blos-8*)	Lysosome transport

Figure 3. Tissue-Specific RNA-Seq Analysis Reveals Intestinal Targets of Neuronal UPR^{ER} Activation

(A) Schematic overview of RNA-seq analysis.

(B) RNA-seq scatterplot showing log_2 expression levels of genes in neuronal *xbp-1s*-expressing animals compared to control worms. Points in blue highlight differentially regulated genes.

(C) Gene ontology of transcripts upregulated in the intestine of animals expressing neuronal *xbp-1s*. The p value for each enrichment is shown.

(D) Upregulated genes classified as lysosomal or lysosome related. Genes were subdivided by their function within the lysosome: proteolysis (green), lipid or carbohydrate metabolism (yellow), DNA degradation (pink), defense responses (blue), transporters (purple), vacuolar ATPase subunits (orange), and transport (peach).

(E) qRT-PCR analysis of lysosomal genes *asp-3*, *lipl-1*, *cdr-1*, and *vha-18* in intestinal cells from control and *rab-3p::xbp-1s* animals. Bar graphs represent mean transcript levels normalized to control, from 3 independent biological replicates. Error bars represent SEM. Significance was assessed by one-way ANOVA with Tukey's multiple comparisons test, $*p < 0.05$, $****p < 0.0001$. See also Figures S2 and S3 and Data S1.

To understand how communication of UPR^{ER} activation to the intestine leads to these downstream effects, we decided to determine the transcriptional changes occurring in intestinal cells when *xbp-1s* is expressed in neurons. To do this, we adapted an existing *C. elegans* cellular dissociation protocol and a tissue-specific RNA-seq method previously used to determine transcriptional changes in neurons [20, 21]. GFP-labeled intestinal cells from wild-type and *rab-3p::xbp-1s* animals expressing *ges-1p::GFP*, an intestine-specific GFP marker [22], were isolated from dissociated worm cells by fluorescence-activated cell sorting (FACS), and RNA-seq was used to identify transcripts altered by neuronal *xbp-1s* (Figure S2C). To validate our approach, we examined the levels of a range of transcripts in our intestinal samples. Confirming previous observations that neuronal *xbp-1s*-expressing animals upregulate *xbp-1s* activity in the intestine, we observed higher levels of *xbp-1s* transcripts in isolated intestinal cells from *rab-3p::xbp-1s* animals compared to wild type (Figure S2D) [6]. Additionally, to confirm enrichment of intestine-specific relative to muscle- and neuron-specific transcripts, expression levels of tissue-specific genes from the Princeton "Tissue-specific expression predictions for *C. elegans*" database were examined; this demonstrated enrichment of intestinal transcripts (Figure S2E). Finally, qPCR was used to examine expression levels of intestinal, neuronal, hypodermal, and muscle-specific genes in our RNA samples, identifying significant enrichment of intestinal, and underrepresentation of other tissue-specific transcripts (Figure S2F).

Comparison of transcripts in neuronal *xbp-1s* and control worms revealed broad transcriptional remodeling in the intestine, with 1,388 genes upregulated and 830 downregulated in neuronal *xbp-1s* animals (Figures 3A and 3B; Data S1). The gene ontology categories most enriched among these differentially regulated transcripts included the endoplasmic reticulum unfolded protein response, endoplasmic reticulum lumen, and protein processing in the endoplasmic reticulum, as expected (Figure 3C; Data S1). Upregulated genes included known UPR^{ER} components such as *xbp-1* and *hsp-4*, confirming that neuronal XBP-1s can trigger activation of the UPR^{ER} in the intestine cell non-autonomously [6].

However, several other gene classes were also differentially regulated, suggesting that the transcriptional landscape of the cell non-autonomous UPR^{ER} extends beyond canonical UPR^{ER} targets. Of these, a prominent upregulation of lysosomal genes

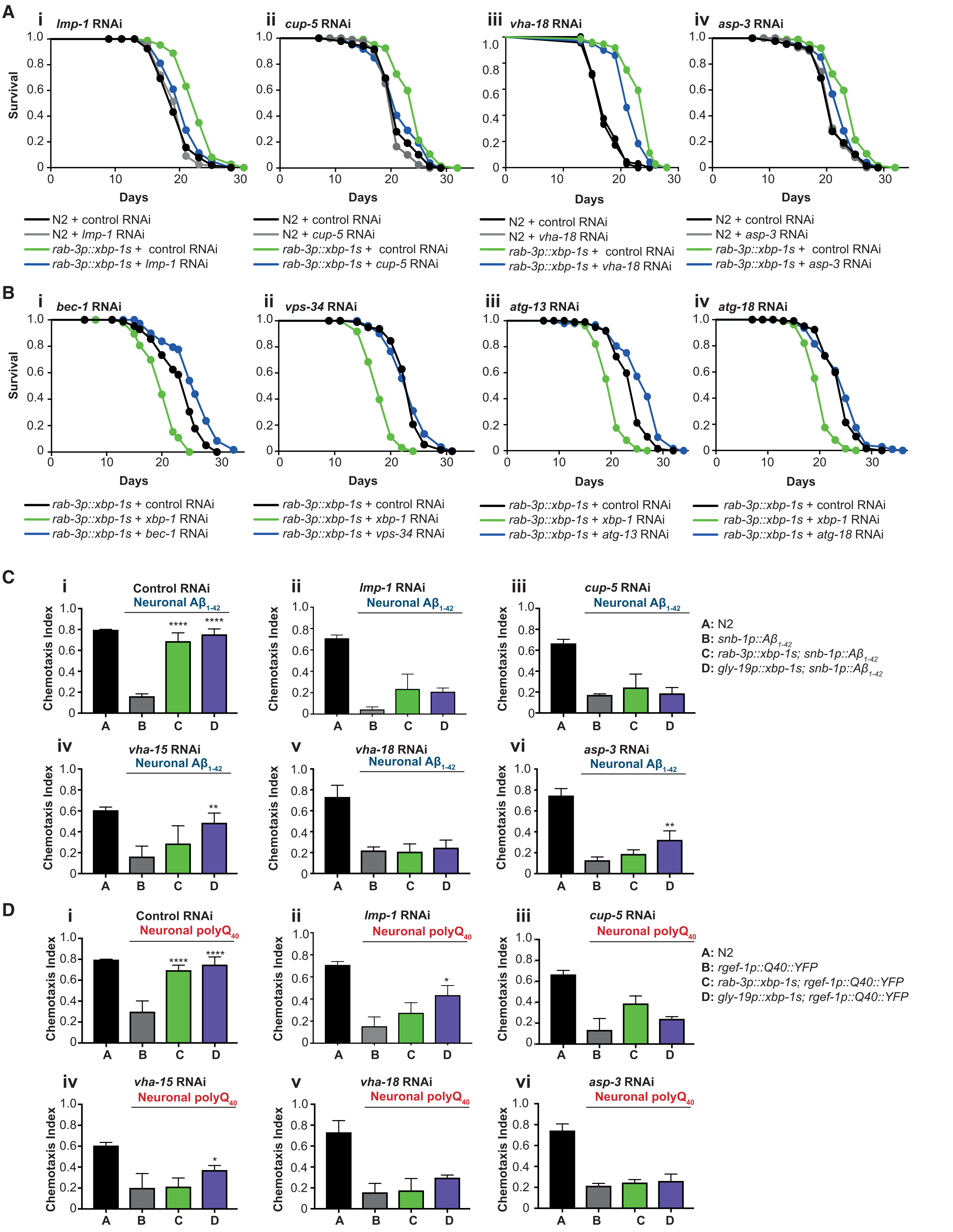

(legend on next page)

stood out as a candidate mechanism for XBP-1s-mediated reductions in toxic protein levels, as lysosomes and autophagy have been implicated in clearance of protein aggregates (Figures 3C and 3D) [23]. qPCR analysis of a selection of these genes confirmed their upregulation in neuronal *xbp-1s*-expressing animals (Figure 3E). We also found that a majority of these lysosomal genes had putative XBP-1s binding sites in their promoters, suggesting that they may represent direct transcriptional targets (Figure S3A). Lysosomal genes upregulated by XBP-1s are involved in a range of lysosomal functions and include proteases, suggesting that increased lysosomal proteolysis might underlie the effects of XBP-1s on proteotoxicity (Figure 3D). Interestingly, when we examined transcript levels of several of these lysosomal genes in short-lived animals expressing *xbp-1s* in muscle cells, we found that they were not increased—in fact, transcription of these lysosomal genes was reduced (Figure S3B), suggesting a correlation between lysosomal gene transcription and longevity. We therefore decided to explore the role of lysosomes in XBP-1s-mediated improvements to lifespan and proteostasis.

Lysosome Function Is Required for Extended Longevity and Protection against Proteotoxicity by Neuronal *xbp-1s*

To determine whether lysosome function is required for the long lifespan of *rab-3p::xbp-1s* animals, we used RNAi to knock down LMP-1, a homolog of mammalian LAMP1, which localizes to the membranes of lysosomes (and some endosomal vesicles), thus inhibiting lysosome function. *Lmp-1* knockdown suppressed the extended lifespan of *rab-3p::xbp-1s* worms, while longevity of N2 animals was unaffected (Figure 4Ai; Table S1). We also knocked down another lysosomal gene, encoding the lysosomal membrane-localized mucolipin CUP-5. Again, RNAi against *cup-5* significantly suppressed *rab-3p::xbp-1s*-associated lifespan extension, while N2 lifespan was unchanged (Figure 4Aii; Table S1). RNAi against two lysosomal genes identified by RNA-seq analysis, the vacuolar ATPase subunit *vha-18* and the aspartyl protease *asp-3*, caused small but significant reductions to the longevity of *rab-3p::xbp-1s* animals, suggesting that *xbp-1s*-mediated induction of these genes may contribute to extended lifespan (Figures 4Aiii–4Aiv; Table S1). In contrast, knockdown of four autophagy mediators, *bec-1*, *vps-34*, *atg-13*, and *atg-18*, did not reduce longevity in animals expressing neuronal *xbp-1s*; in fact, lifespan was sometimes slightly extended in these animals (Figure 4B). This suggests, surprisingly, a role for lysosomes downstream of *xbp-1s* that may be independent of canonical macroautophagy.

We also knocked down lysosomal genes in animals expressing neuronal $A\beta_{1-42}$ and $polyQ_{40}$, in combination with neuronal and intestinal *xbp-1s*. Rescue of chemotaxis upon *xbp-1s* expression (Figures 4Ci and 4Di) was substantially abrogated by RNAi against *lmp-1*, *cup-5*, *vha-18*, *asp-3*, or the vacuolar ATPase subunit *vha-15* (Figures 4Cii–4Cvi and 4Dii–4Dvi). Lysosomes therefore play an important role in protection against neuronal proteotoxicity provided by neuronal and intestinal *xbp-1s*. Knockdown of the autophagy genes *bec-1*, *vps-34*, *atg-13*, and *atg-18*, however, had little to no effect on chemotaxis in these animals, suggesting that macroautophagy is not required for this protection (Figures S4A and S4B). To rule out the possibility that our autophagy gene RNAi clones were ineffective at inhibiting autophagy, we quantified autophagosome formation in animals expressing the *C. elegans* Atg8 ortholog LGG-1 tagged with GFP, upon RNAi against genes involved in autophagy. Using knockdown of the insulin-like receptor *daf-2*

Figure 4. Lysosome Function Is Required for the Effects of *xbp-1s* on Lifespan and Proteotoxicity

(A) Lifespan analysis of N2 and *rab-3p::xbp-1s* animals grown on control (empty vector) or lysosomal gene RNAi: (i) *lmp-1*, (ii) *cup-5*, (iii) *vha-18*, (iv) *asp-3*. Graphs were plotted as Kaplan-Meier survival curves, and p values were calculated by Mantel-Cox log-rank test; N = 80–120 animals per lifespan.

(i) N2, control (black), median lifespan 19 days; N2, *lmp-1* (gray), median lifespan 21 days, p = 0.8860; *rab-3p::xbp-1s*, control (green), median lifespan 23 days; *rab-3p::xbp-1s*, *lmp-1* (blue), median lifespan 21 days, $p < 0.0001$.

(ii) N2, control (black), median lifespan 21 days; N2, *cup-5* (gray), median lifespan 21 days, p = 0.0950; *rab-3p::xbp-1s*, control (green), median lifespan 25 days; *rab-3p::xbp-1s*, *cup-5* (blue), median lifespan 21 days, $p < 0.01$.

(iii) N2, control (black), median lifespan 17 days; N2, *vha-18* (gray), median lifespan 17 days, p = 0.8118; *rab-3p::xbp-1s*, control (green), median lifespan 25 days; *rab-3p::xbp-1s*, *vha-18* (blue), median lifespan 23 days, $p < 0.0001$.

(iv) N2, control (black), median lifespan 21 days; N2, *asp-3* (gray), median lifespan 21 days, p = 0.9913; *rab-3p::xbp-1s*, control R (green), median lifespan 25 days; *rab-3p::xbp-1s*, *asp-3* (blue), median lifespan 23 days, $p < 0.001$.

(B) Lifespan analysis of *rab-3p::xbp-1s* animals grown on control (empty vector) and *xbp-1* RNAi, as well as RNAi against autophagy genes: (i) *bec-1*, (ii) *vps-34*, (iii) *atg-13*, (iv) *atg-18*. Graphs were plotted as Kaplan-Meier survival curves, and p values were calculated by Mantel-Cox log-rank test.

(i) *rab-3p::xbp-1s*, control (black), median lifespan 25 days; *rab-3p::xbp-1s*, *xbp-1* (green), median lifespan 20 days, $p < 0.0001$; *rab-3p::xbp-1s*, *bec-1* (blue), median lifespan 26 days, $p < 0.0001$.

(ii) *rab-3p::xbp-1s*, control (black), median lifespan 25 days; *rab-3p::xbp-1s*, *xbp-1* (green), median lifespan 20 days, $p < 0.0001$; *rab-3p::xbp-1s*, *vps-34* (blue), median lifespan 25 days, p = 0.0976.

(iii) *rab-3p::xbp-1s*, control (black), median lifespan 25 days; *rab-3p::xbp-1s*, *xbp-1* (green), median lifespan 21 days, $p < 0.0001$; *rab-3p::xbp-1s*, *atg-13* (blue), median lifespan 27 days, $p < 0.0001$.

(iv) *rab-3p::xbp-1s*, control (black), median lifespan 25 days; *rab-3p::xbp-1s*, *xbp-1* (green), median lifespan 21 days, $p < 0.0001$; *rab-3p::xbp-1s*, *atg-18* (blue), median lifespan 25 days, p = 0.1527.

(C) Chemotaxis ability in animals expressing $A\beta_{1-42}$ in neurons in combination with neuronal and intestinal *xbp-1s*, grown on (i) control (empty vector), (ii) *lmp-1*, (iii) *cup-5*, (iv) *vha-15* (from L4), (v) *vha-18*, or (vi) *asp-3* RNAi. Graphs represent mean chemotaxis index ± SD. N = 65–140 animals per assay; each assay was independently replicated 3 times. Significance between neuronal $A\beta_{1-42}$ (B) and *xbp-1s*-expressing (C/D) strains was assessed by two-way ANOVA with Dunnett's multiple comparisons test, ****$p < 0.0001$.

(D) Chemotaxis ability in animals expressing $polyQ_{40}$ in neurons in combination with neuronal and intestinal *xbp-1s*, grown on (i) control (empty vector), (ii) *lmp-1*, (iii) *cup-5*, (iv) *vha-15* (from L4), (v) *vha-18*, or (vi) *asp-3* RNAi. Graphs represent mean chemotaxis index ± SD. N = 80–170 animals per assay; each assay was independently replicated 3 times. Significance between neuronal $polyQ_{40}$ (B) and *xbp-1s*-expressing (C/D) strains was assessed by two-way ANOVA with Dunnett's multiple comparisons test, **$p < 0.01$, ***$p < 0.001$, ****$p < 0.0001$. See also Figure S4 and Table S1.

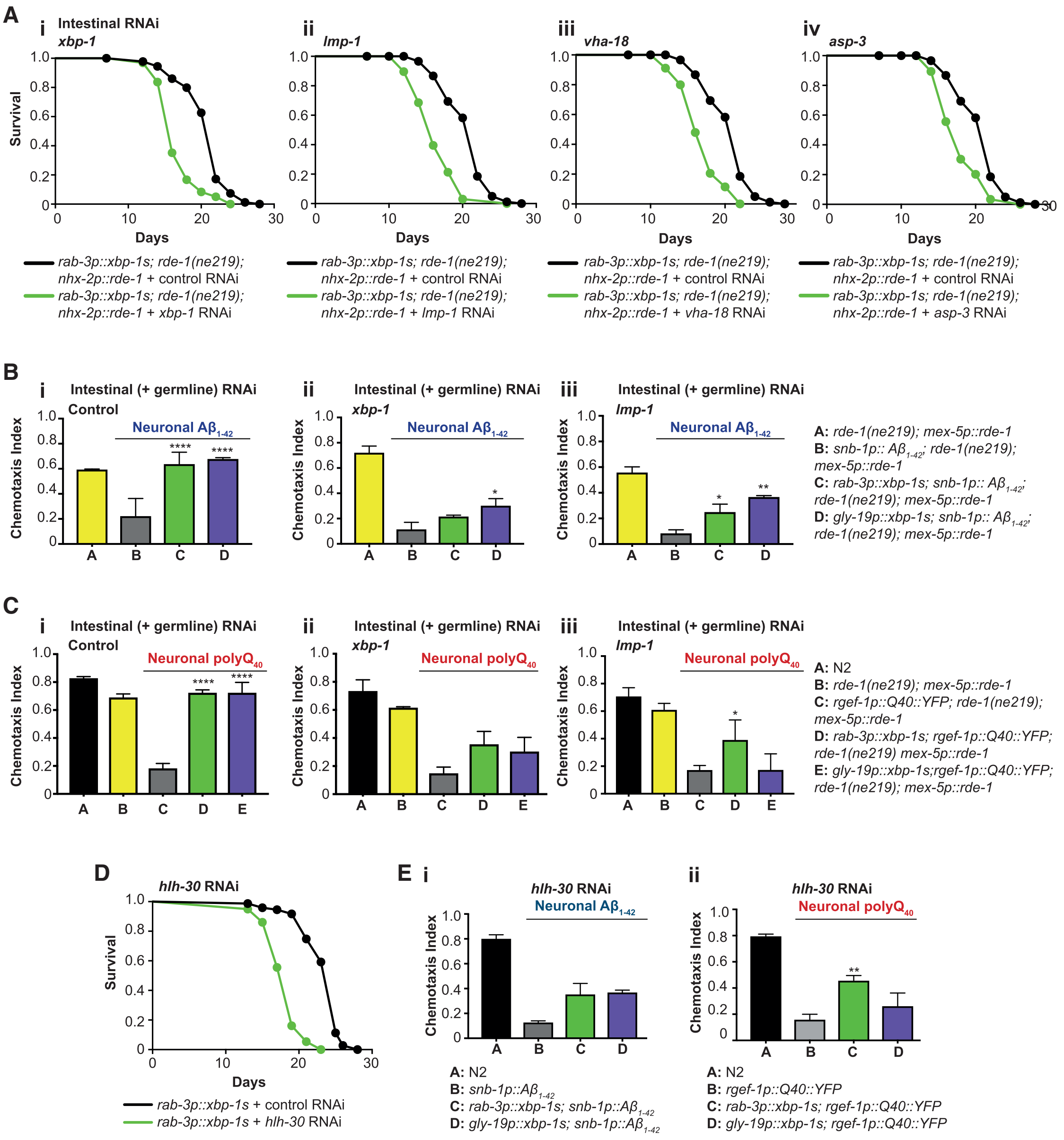

Figure 5. Intestinal Knockdown of Lysosomal Genes Abolishes Lifespan Extension and Improved Proteostasis in *xbp-1s*-Expressing Animals

(A) Lifespan analysis of *rab-3p::xbp-1s; rde(n219); nhx-2p::rde-1* animals grown on control (empty vector) or (i) *xbp-1*, (ii) *lmp-1*, (iii) *vha-18*, or (iv) *asp-3* RNAi. Graphs were plotted as Kaplan-Meier survival curves, and p values were calculated by Mantel-Cox log-rank test; N = 80–120 animals per lifespan.

(i) *rab-3p::xbp-1s; rde(n219); nhx-2p::rde-1*, control (black), median lifespan 22 days; *rab-3p::xbp-1s; rde(n219); nhx-2p::rde-1*, *xbp-1* (green), median lifespan 16 days, $p < 0.0001$.

(ii) *rab-3p::xbp-1s; rde(n219); nhx-2p::rde-1*, control (black), median lifespan 22 days; *rab-3p::xbp-1s; rde(n219); nhx-2p::rde-1*, *lmp-1* (green), median lifespan 16 days, $p < 0.0001$.

(iii) *rab-3p::xbp-1s; rde(n219); nhx-2p::rde-1*, control (black), median lifespan 22 days; *rab-3p::xbp-1s; rde(n219); nhx-2p::rde-1*, *vha-18* (green), median lifespan 16 days, $p < 0.0001$.

(iv) *rab-3p::xbp-1s; rde(n219); nhx-2p::rde-1*, control (black), median lifespan 22 days; *rab-3p::xbp-1s; rde(n219); nhx-2p::rde-1*, *asp-3* R (green), median lifespan 18 days, $p < 0.0001$.

(B) Chemotaxis ability in *rde(n219); mex-5p::rde-1* animals expressing $A\beta_{1-42}$ in neurons in combination with neuronal or intestinal *xbp-1s*, grown on (i) control (empty vector), (ii) *xbp-1*, or (iii) *lmp-1* RNAi. Graphs represent mean chemotaxis index ± SD. N = 80–130 animals per assay; each assay was independently replicated 3 times. Significance between neuronal $A\beta_{1-42}$ (B) and *xbp-1s*-expressing (C and D) strains was assessed by two-way ANOVA with Dunnett's multiple comparisons test, $^*p < 0.05$, $^{**}p < 0.01$, $^{****}p < 0.0001$.

(C) Chemotaxis ability in *rde(n219); mex-5p::rde-1* animals expressing $polyQ_{40}$ in neurons in combination with neuronal or intestinal *xbp-1s*, grown on (i) control (empty vector), (ii) *xbp-1*, or (iii) *lmp-1* RNAi. Graphs represent mean chemotaxis index ± SD. N = 80–130 animals per assay; each assay was independently

(legend continued on next page)

to induce autophagosome formation, combined with RNAi against autophagy genes, we found that autophagosome formation was significantly suppressed in animals grown on RNAi against *bec-1*, *vps-34*, *atg-13*, or *atg-18*, confirming that these treatments effectively inhibit autophagy (Figure S4C).

Intestinal Knockdown of Lysosomal Genes Suppresses Lifespan Extension and Protection against Proteotoxicity by *xbp-1s*

Neuronal *xbp-1s* expression activates the UPR^{ER} in the intestine, leading to upregulation of lysosomal genes in this tissue (Figure 3) [6]. In addition, expression of *xbp-1s* only in intestinal cells is sufficient to protect against proteotoxicity in multiple tissues. We therefore wondered whether intestine-specific knockdown of lysosomal genes would affect the ability of neuronal *xbp-1s* to extend lifespan and protect against proteotoxicity. To address this, we used an *rde-1(n219)* mutation that prevents effective RNAi, coupled with replacement of *rde-1* only in intestinal cells [24, 25]. Intestine-specific *rde-1* expression utilized the *nhx-2p* promoter; however, because these animals were somewhat short lived, we also used animals in which *rde-1* was replaced in both the intestine and germline (but no additional somatic tissues) using the *mex-5p* promoter [26]. First, we established that *rab-3p::xbp-1s* animals were still long lived in an *rde-1(n219)* mutant background and when *rde-1* was expressed under either intestinal promoter (Figures S5Ai–S5Aiii; Table S1). We then confirmed that *xbp-1* RNAi did not shorten the lifespan of *rab-3p::xbp-1s; rde-1(n219)* animals (Figure S5Aiv; Table S1). However, when *rde-1* expression was rescued only in the intestine, *xbp-1* RNAi was able to significantly reduce longevity (Figure 5Ai). Likewise, intestinal knockdown of the lysosomal genes *lmp-1*, *vha-18*, and *asp-3* in *rab-3p::xbp-1s; rde-1(n219); nhx-2p::rde-1* animals also significantly reduced lifespan, demonstrating that intestinal lysosome function is required for extended longevity upon neuronal *xbp-1s* expression (Figures 5Aii–5Aiv; Table S1). The same effects were seen in animals expressing *rde-1* under the *mex-5* promoter (Figure S5B; Table S1).

Next, we asked whether intestinal knockdown was sufficient to abrogate neuronal *xbp-1s*-mediated protection against proteotoxicity in animals expressing neuronal $A\beta_{1-42}$ or $polyQ_{40}$. We found that intestinal *xbp-1* or *lmp-1* knockdown prevented rescue of chemotaxis by neuronal and intestinal *xbp-1s* expression in both models (Figures 5B and 5C), suggesting that the activity of lysosomes in the intestine is important to the protection against proteotoxicity mediated by neuronal or intestinal *xbp-1s*. Finally, to determine whether *xbp-1s* in the intestine is required for upregulation of lysosomal gene targets in this tissue, we used qRT-PCR in *rab-3p::xbp-1s; rde-1(n219); nhx-2p::rde-1* animals to ask whether intestine-specific knockdown of *xbp-1* altered expression of lysosomal genes. Indeed, intestine-specific *xbp-1* RNAi was sufficient to prevent upregulation of several lysosomal transcripts in these animals (Figure S5C).

HLH-30 Is Necessary for Lifespan Extension and Protection against Proteotoxicity by *xbp-1s*

The *C. elegans* homolog of the TFEB transcription factor, HLH-30, is required for lysosomal biogenesis and activation [27, 28]. We found that several of the lysosomal genes upregulated in *xbp-1s*-expressing animals had predicted HLH-30-binding CLEAR domains in their promoters (Figure S3A) and therefore asked whether HLH-30 was required for neuronal *xbp-1s* to extend lifespan. We found that *hlh-30* knockdown substantially reduced longevity in *rab-3p::xbp-1s* animals (Figure 5D; Table S2). Indeed, intestine-specific RNAi against *hlh-30* was sufficient to abolish lifespan extension, and adult-only intestinal knockdown of either *hlh-30* or *xbp-1*, by transfer of day 1 adults to RNAi, was again sufficient to reduce longevity (Figures S6A–S6C; Table S2).

We also asked whether RNAi against *hlh-30* could abolish *xbp-1s*-mediated protection against proteotoxicity. Again, rescue of chemotaxis by *xbp-1s* was substantially reduced by *hlh-30* knockdown in both $A\beta_{1-42}$ and $polyQ_{40}$ models, confirming that functional lysosomes are required for improved proteostasis (Figure 5E). Intestine-specific knockdown of *hlh-30* prevented induction of lysosomal genes in *rab-3p::xbp-1s* animals (Figure S5C). However, unlike starvation, a known HLH-30-activating stimulus, neuronal *xbp-1s* did not induce nuclear localization of HLH-30:GFP, suggesting that *xbp-1s* may not directly activate HLH-30 but may instead rely on its basal roles in lysosome biogenesis and activity (Figure S6D).

Lysosomes Are Required for *xbp-1s*-Induced Reductions in PolyQ Levels

Expression of *xbp-1s* in the intestine or nervous system of *C. elegans* reduces levels of toxic proteins expressed in multiple tissues. To determine whether lysosomes are required for this reduction, *lmp-1* was knocked down in worms expressing neuronal and intestinal $polyQ_{40}$ concurrently with neuronal or intestinal *xbp-1s*, and $polyQ_{40}$::YFP levels determined by native PAGE. Upon *xbp-1s* expression, $polyQ_{40}$ levels were reduced; however, upon *lmp-1* knockdown, this effect was diminished (Figure 6A), suggesting that lysosomes may be involved in XBP-1s-mediated $polyQ_{40}$ degradation.

replicated 3 times. Significance between neuronal $polyQ_{40}$ (B) and *xbp-1s*-expressing (C and D) strains was assessed by two-way ANOVA with Dunnett's multiple comparisons test, *$p < 0.05$, ****$p < 0.0001$.

(D) Lifespan analysis of *rab-3p::xbp-1s* animals grown on control (empty vector) or *hlh-30* RNAi. *rab-3p::xbp-1s*, control (black), median lifespan 25 days; *rab-3p::xbp-1s*, *hlh-30* (green), median lifespan 19 days, $p < 0.0001$. Graphs were plotted as Kaplan-Meier survival curves, and p values were calculated by Mantel-Cox log-rank test; N = 80–120 animals per lifespan.

(E) (i) Chemotaxis ability in animals expressing $A\beta_{1-42}$ in neurons in combination with neuronal and intestinal *xbp-1s*, grown on control (empty vector) or *hlh-30* RNAi. Bar graphs represent mean chemotaxis index ± SD. N = 70–130 animals per assay; each assay was independently replicated 3 times. Significance was assessed by two-way ANOVA with Dunnett's multiple comparisons test.

(ii) Chemotaxis ability in animals expressing $polyQ_{40}$ in neurons in combination with neuronal and intestinal *xbp-1s*, grown on control (empty vector) or *hlh-30* RNAi. Bar graphs represent mean chemotaxis index ± SD. N = 70–130 animals per assay; each assay was independently replicated 3 times. Significance was assessed by two-way ANOVA with Dunnett's multiple comparisons test, **$p < 0.01$. See also Figures S5 and S6 and Table S1.

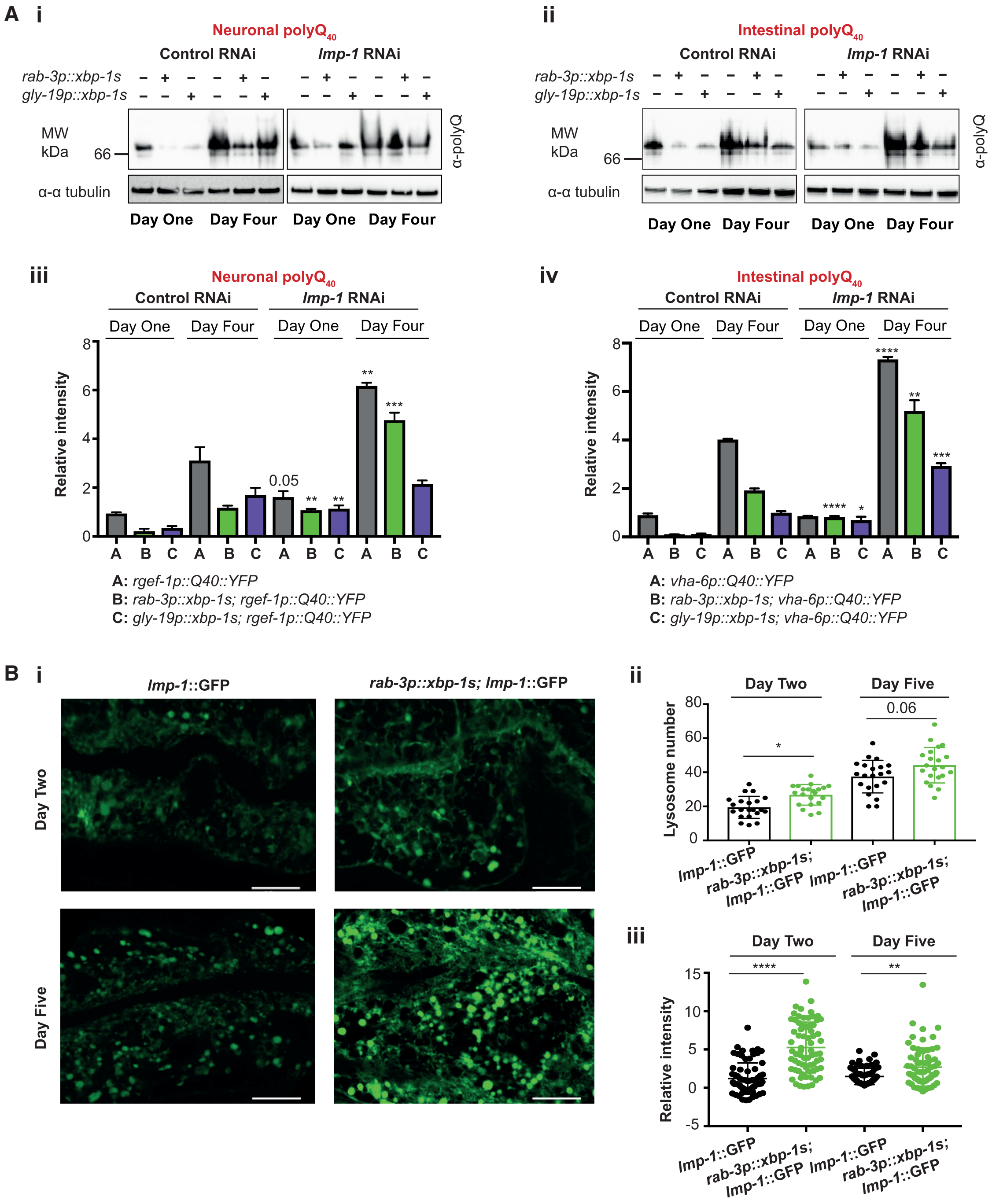

Figure 6. Lysosomes Are Required for *xbp-1s*-Mediated Reduction in PolyQ Levels

(A) Western blot analysis of (i) neuronal or (ii) intestinal polyQ$_{40}$::YFP at day 1 and day 4 of adulthood, expressed in combination with neuronal and intestinal *xbp-1s* and grown on control (empty vector) or *lmp-1* RNAi. Lysates containing total proteins were resolved under native conditions and blotted with an anti-polyQ antibody. Tubulin levels were probed with α-α-tubulin as a loading control. Data are representative of at least 3 independent experiments. Quantification of (iii) neuronal or (iv) intestinal polyQ$_{40}$::YFP was conducted using ImageJ. Graphs represent mean band intensity relative to day 1 polyQ ± SD. Statistical significance was assessed between control and *lmp-1* for each genotype and time point, using two-way ANOVA with Tukey's multiple comparisons, *$p < 0.05$, **$p < 0.01$, ***$p < 0.001$, ****$p < 0.0001$.

(B) (i) Representative confocal images of the intestine of animals expressing LMP-1::GFP, with and without *rab-3p::xbp-1s*. Animals were grown on OP50 and imaged at days 2 and 5 of adulthood. Imaging was performed at X63 magnification. Scale bar, 10 μm.

(legend continued on next page)

Neuronal *xbp-1s* Increases Lysosomal LMP-1 Labeling

As *xbp-1s* expression increases transcription of lysosomal genes, and lysosome activity is required for clearance of toxic species, we asked whether the number of lysosomes in intestinal cells was increased by neuronal *xbp-1s* expression. To do this, we used animals expressing GFP-tagged LMP-1 to label lysosomes [29]. When LMP-1::GFP was combined with *rab-3p::xbp-1s*, a small increase in lysosome number was observed (Figures 6Bi and 6Bii), while when we examined animals expressing LGG-1 tagged with GFP, we found that there was no increase in number of GFP::LGG-1-labeled intestinal autophagosomes in animals expressing neuronal *xbp-1s* (Figure S6E) [30]. Furthermore, when we measured the GFP fluorescence intensity of individual LMP-1::GFP-labeled lysosomes, we found a significant increase in LMP-1 labeling in *rab-3p::xbp-1s* nematodes (Figures 6Bi and 6Biii). This might suggest an increase in LMP-1 recruitment to lysosomes and led us to speculate that their activity may be altered by neuronal *xbp-1s*.

Neuronal *xbp-1s* Increases Lysosomal Acidity

Measures of autophagic flux in *C. elegans* suggest a decline in lysosomal activity with age [31]. The activity of lysosomes depends on their acidity, which is necessary for the activity of lysosomal hydrolases. We therefore asked whether neuronal *xbp-1s* could increase or preserve lysosomal acidity with age. To explore this, we utilized a technique to sensitively measure lysosomal acidity in the proximal intestine of the worm [32]. Using carboxy-2',7'-dichlorofluorescein diacetate (cDCFDA), which is hydrolyzed to fluorescent cDCF in acidic conditions, we found that neuronal *xbp-1s* significantly increases the acidity of intestinal lysosomes, particularly as animals age; at day 2, and especially at day 5 of adulthood, lysosomes of *rab-3p::xbp-1s* animals were significantly more acidic, and larger, than those in wild-type worms (Figure 7A). While tissue-specific promoters may lose some specificity from day 5 [33], we were reassured by the observation that increased acidity was already present at day 2. Greater activity of lysosomal protein hydrolases in *rab-3p::xbp-1s* animals was also observed—while activity declined between day 2 and 5 in wild-type animals, it increased significantly in *rab-3p::xbp-1s* nematodes (Figure 7B). To ensure that cDCFDA was truly detecting changes to lysosomal acidity, we confirmed that RNAi against *daf-2* increased acidity-dependent staining of lysosomes, as expected, and that knockdown of the downstream transcription factor *daf-16* reduced acidity (Figure S7A). In addition, staining was reduced by knockdown of two subunits of the acidifying vacuolar ATPase, *vha-2* and *vha-8* (Figure S7B). We then asked whether RNAi against the vacuolar ATPase subunit upregulated by *xbp-1s*, *vha-18* also suppressed lysosomal acidity. We found that, indeed, *vha-18* knockdown reduced lysosomal acidification in *rab-3p::xbp-1s* animals, suggesting that elevated levels of this subunit might be a mechanism by which *xbp-1s* orchestrates higher lysosomal acidity and activity (Figure 7C).

This difference in lysosome acidity was also apparent in animals expressing neuronal polyQ$_{40}$. Background intestinal fluorescence was substantially increased in these animals, possibly reflecting an increase in overall cellular acidity; this increase, as well as the acidity of lysosomes, was lost by day 5 of adulthood but was significantly increased in animals expressing neuronal *xbp-1s*, suggesting a mechanism for improved polyQ clearance (Figure S7C).

To confirm these findings, we stained intestinal lysosomes with LysoTracker, another pH-dependent dye. Lysosomal staining was again increased in animals expressing neuronal or intestinal *xbp-1s* at both day 2 and particularly at day 5 of adulthood; neuronal polyQ$_{40}$ was also associated with age-dependent collapse in lysosomal acidity and number, rescued by co-expression of neuronal or intestinal *xbp-1s* (Figures S7D and S7E). Similarly, muscle-specific polyQ$_{35}$ decreased number and staining of intestinal lysosomes by day 5, rescued by expression of neuronal but not muscle-driven *xbp-1s* (Figure S7F). Together, these results confirm that *xbp-1s* expression in neurons or the intestine increases the acidity of intestinal lysosomes, rescuing the decline seen with age and in animals expressing misfolded proteins.

Neuronal *xbp-1s* Promotes Maturation of Autophagosomes to Autolysosomes

Finally, we asked whether *xbp-1s* expression was able to promote the maturation of autophagosomes to autolysosomes. To address this, we used a tandem tag reporter, with GFP and mCherry conjugated to LGG-1 [31]. Autophagosomes are labeled with both GFP and mCherry, while the acidification associated with maturation of autophagosomes into autolysosomes quenches GFP, so that autolysosomes are only labeled with mCherry. Using this system, we observed a striking enrichment in autolysosomes at day 2 and especially at day 5 in neuronal *xbp-1s* animals, while autophagosome number decreased in older animals (Figure 7D). This suggests that autolysosomes are more prevalent, while autophagosome turnover may be increased, in *xbp-1s*-expressing animals, which would be consistent with increased function of the lysosomal compartment. Together, these results suggest that *xbp-1s* transcriptionally activates lysosomes in the intestine of *C. elegans*, leading to improved clearance of proteotoxic species and increased longevity (Figure 7E).

DISCUSSION

In this work, lysosomes are identified as a key organelle mediating the downstream effects of cell non-autonomous UPR^{ER} activation. Expression of *xbp-1s* in neurons or the intestine improves proteostasis across tissues, suppressing the toxicity associated with aggregation-prone proteins by reducing levels of toxic species. This is accompanied by increased transcription of lysosomal genes in the intestine and increases in lysosomal

(ii) Quantification of lysosome number. LMP-1::GFP-positive punctae were counted in 10–15 worms per genotype at day 2 and day 5 of adulthood using ImageJ, from 3 independent biological replicates. Statistical analysis was carried out using one-way ANOVA with Tukey's multiple comparisons test, $^*p < 0.05$.
(iii) Quantification of lysosomal LMP-1::GFP labeling. Fluorescence intensity of LMP-1::GFP per lysosome was quantified using ImageJ in 10–15 worms per genotype, at day 2 and day 5 of adulthood, in 3 biological replicates. Statistical significance was calculated using one-way ANOVA with Tukey's multiple comparisons test, $^{**}p < 0.01$, $^{****}p < 0.0001$. See also Figure S6.

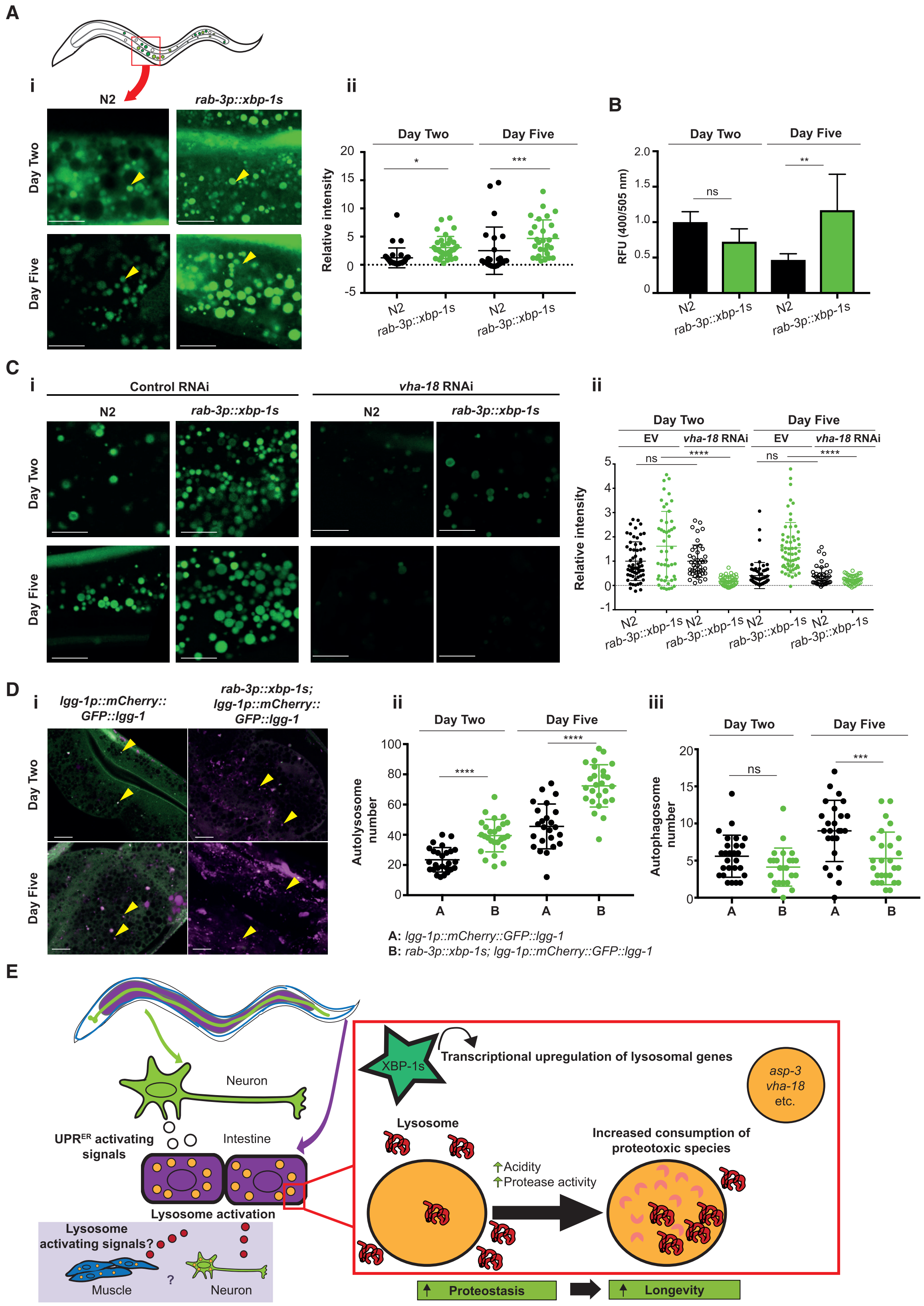

(legend on next page)

acidity and protease activity as animals age. Lysosome function is involved in the striking clearance of misfolded proteins seen when *xbp-1s* is expressed in neurons or the intestine and is required for XBP-1s-mediated improvements in proteostasis and lifespan.

This suggests that lysosomes are important in guarding against proteotoxicity. Indeed, existing evidence suggests that lysosomes play roles in the onset and progression of neurodegenerative diseases associated with protein aggregation [34]. Mutations in lysosomal components have been identified in patients with diseases including Parkinson's and Alzheimer's, and studies of disease-associated proteins in model systems have found that lysosomes are required to prevent onset of proteotoxicity and propagation of protein aggregates between cells [35–39]. Additionally, treatments that activate lysosomes, including overexpression of the lysosome-regulating transcription factor TFEB, increase clearance of disease-associated proteins in cellular and mouse models [39–43]. The UPR^{ER} has also been implicated in the onset and development of neurodegenerative disease, although the roles it plays in these processes are complex and sometimes disputed [44]. Our results suggest that the UPR^{ER} might influence neurodegenerative disease progression through effects on the activity of lysosomes, and consequently the clearance of toxic protein species.

In addition, our study shows that XBP-1s may preferentially reduce levels of soluble species in contexts in which it is protective against proteotoxicity. In neurodegenerative disease, the presence of protein aggregates has been long associated with pathology. However, more recently the role of large protein aggregates in disease progression has been questioned, as an increasing body of work suggests that high molecular weight aggregates may perform protective roles in cells by sequestering more toxic lower-molecular-weight species that are thought to be the main agents of cellular damage [16, 45, 46]. Our findings may therefore support the view that reducing soluble species, rather than insoluble aggregates, is of primary importance in reducing age-associated toxicity.

The communication of UPR^{ER} activation observed in *C. elegans* using the *hsp-4p::GFP* reporter strain occurs specifically between neurons and the intestine, in a unidirectional manner dependent upon neuron-specific secretion [6], and it is XBP-1s expression in these two tissues that leads to extended lifespan and improved proteostasis. Muscle-specific *xbp-1s* does not improve longevity [6] and has only limited beneficial effects on proteostasis. Our results suggest that tissue-specific differences in the genes targeted by XBP-1s may underlie these different effects; muscle-specific *xbp-1s* expression reduces, rather than increases, transcription of lysosomal genes, suggesting a reason for the shorter lifespan of these animals compared to those expressing *xbp-1s* in other tissues.

Conversely, we show here that intestinal *xbp-1s* and intestinal lysosomes play a key role in the beneficial effects of UPR^{ER} activation. Intriguingly, the protection against proteotoxicity afforded by intestinal *xbp-1s* and lysosome activation extends to misfolded proteins expressed across tissues. One possibility is that intestinal *xbp-1s* triggers a signaling event that activates lysosomes in other tissues. How?

Potentially, XBP-1s might directly promote transcription of a lysosome-activating signaling molecule in the intestine, which could then be released through the secretory pathway. Alternatively, activation of lysosomes in this tissue might give rise to a lysosomal signaling event. As well as catabolism, lysosomes play substantive roles in cellular signaling pathways [34]. mTOR, which regulates autophagy as well as other cellular processes, localizes to the lysosomal membrane, and mTOR signaling involves crosstalk with TFEB [47]. In addition, lysosomes can release molecules into the external environment through lysosome-mediated exocytosis, which regulates a

Figure 7. Neuronal *xbp-1s* Increases Lysosomal Acidity

(A) (i) Confocal imaging of lysosomal acidity in N2 and *rab-3p::xbp-1s* intestines. Animals were grown on OP50 and transferred to plates containing cDCFDA 16 h prior to imaging. Imaging was conducted in the anterior intestine at 63× magnification in day 2 and day 5 adults. Yellow arrowheads indicate representative lysosomes. Scale bar, 10 μm.

(ii) Quantification of lysosomal acidity in wild-type and *rab-3p::xbp-1s* intestines. Animals were grown on OP50 and transferred to plates containing cDCFDA 16 h prior to imaging; imaging was conducted as described above, and fluorescence quantified from 3 experiments using ImageJ. Plots represent 5–10 animals per replicate. Significance was assessed between N2 and *rab-3p::xbp-1s* at each time point by one-way ANOVA with Tukey's multiple comparisons, $^{*}p < 0.05$, $^{***}p < 0.001$.

(B) Protease activity in *rab-3p::xbp-1s* and N2 animals. Following incubation with a fluorescent substrate, relative fluorescence units (RFU) at 400/505 nm were measured. Bar graphs represent mean ± SD from 3 independent biological replicates. Significance was calculated using one-way ANOVA with Tukey's multiple comparisons test; ns, not significant, $^{**}p < 0.01$.

(C) (i) Confocal imaging of lysosomal acidity in N2 and *rab-3p::xbp-1s* animals grown on control (empty vector) or *vha-18* RNAi and transferred to plates containing cDCFDA 16 h prior to imaging. Imaging was conducted as described above. Scale bar, 10 μm.

(ii) Quantification of lysosomal acidity in N2 and *rab-3p::xbp-1s* animals grown on control (empty vector) or *vha-18* RNAi. Animals were imaged as above, and quantification of fluorescence from 3 experiments were carried out using ImageJ. Plots represent 8–10 animals per replicate. Significance was assessed between N2 and *rab-3p::xbp-1s* at each time point by one-way ANOVA with Tukey's multiple comparisons, $^{****}p < 0.0001$.

(D) (i) *lgg-1p::mCherry::GFP::lgg-1* and *rab-3p::xbp-1s; lgg-1p::mCherry::GFP::lgg-1* animals were grown on OP50 and imaged at days 2 and 5 of adulthood at X63 magnification. Arrowheads in *lgg-1p::mCherry::GFP::lgg-1* indicate representative autophagosomes; arrowheads in *rab-3p::xbp-1s; lgg-1p::mCherry::GFP::lgg-1* indicate representative autolysosomes. Scale bar, 10 μm.

(ii) Quantification of autolysosomes. mCherry::LGG-1-positive (magenta) punctae were counted in 10–15 worms per genotype at day 2 and day 5 of adulthood using ImageJ. Data are derived from 3 independent biological replicates. Statistical analysis was carried out using one-way ANOVA with Tukey's multiple comparisons test, $^{****}p < 0.0001$.

(iii) Quantification of autophagosomes. mCherry::LGG-1::GFP-positive (white) punctae were counted in 10–15 worms per genotype at day 2 and day 5 of adulthood using ImageJ. Data are derived from 3 independent biological replicates. Statistical analysis was carried out using one-way ANOVA with Tukey's multiple comparisons test, $^{***}p < 0.001$.

(E) Schematic of the regulation of lifespan and proteostasis by neuronal and intestinal *xbp-1s* through increased lysosome activity. See also Figure S7.

variety of physiological processes [48–50]. They also play roles in lipid signaling pathways that modulate aging—lysosomal lipases can release lipid-derived molecules able to mediate nuclear responses that promote longevity [51]. Future work to measure the number and pH status of lysosomes in tissues other than the intestine would confirm directly whether lysosome activation is achieved throughout the organism following intestinal UPR^{ER} activation. The identification of such a lysosome-activating signaling pathway would then be of great interest.

Lysosome activity is critical for extension of longevity by neuronal *xbp-1s*. Fusion with lysosomes is the end point of autophagy, and a wealth of data links autophagy to aging and neurodegeneration [52, 53]. However, we do not observe a requirement for autophagy genes in *xbp-1s*-mediated lifespan extension. Some evidence already suggests that, as here, lysosomes may play autophagy-independent roles in aging and proteostasis. In the *C. elegans* germline, for example, rejuvenation of the proteome and clearance of aggregates depends upon the lysosome, without a requirement for autophagy. Similarly, prevention of protein aggregate seeding and spreading in the worm depends on lysosomes, but not autophagosomes [39, 54].

Lysosomes have been shown to decline in function with age; measures of autophagic flux in *C. elegans* suggest age-dependent deterioration in lysosome activity, and, in yeast, age-associated decline in the acidity of the lysosome-like vacuole limits lifespan; preventing this decline extends longevity [31, 55]. Vacuolar acidity is increased by caloric restriction, leading to lifespan extension, and acidic vacuoles are restored in daughter cells upon budding, renewing lifespan [55]. In *C. elegans* oocytes, sperm trigger the acidification of lysosomes through the activation of the vacuolar H^+-ATPase, clearing protein aggregates and renewing the proteostasis state of the cell [54].

This accumulating evidence suggests that vacuolar or lysosomal acidification could act as a molecular "switch" to rejuvenate the proteome of cells and restore youthfulness. Our results indicate that *xbp-1s* may be able to activate this lysosomal switch through physiological inter-tissue signaling. Understanding the nature of these signals, both those that communicate UPR^{ER} activity between neurons and the intestine, and those that may communicate lysosomal activation between the intestine and other tissues, is now an important goal for research into aging and neurodegeneration.

STAR★METHODS

Detailed methods are provided in the online version of this paper and include the following:

- KEY RESOURCES TABLE
- LEAD CONTACT AND MATERIALS AVAILABILITY
- EXPERIMENTAL MODEL AND SUBJECT DETAILS
  - *C. elegans* maintenance and RNAi knockdown
- METHOD DETAILS
  - Chemotaxis assays
  - Paralysis assays
  - Osmoregulatory assays
  - Motility assays
  - F-actin staining
  - Fluorescence microscopy
  - Confocal microscopy
  - Protein extraction and Western Blotting
  - Quantification of protein band intensity
  - *C. elegans* cellular dissociation
  - *C. elegans* cellular isolation by FACS
  - RNA extraction and amplification
  - Library preparation, RNA sequencing and analysis
  - Quantitative RT-PCR
  - Lifespan analysis
  - Quantification of lysosomes
  - Quantification of autophagosomes
  - Lysosome acidity assays
  - LysoTracker staining
  - Protease activity assays
  - Tandem quantification of autophagosomes and autolysosomes
- QUANTIFICATION AND STATISTICAL ANALYSIS
- DATA AND CODE AVAILABILITY

SUPPLEMENTAL INFORMATION

Supplemental Information can be found online at https://doi.org/10.1016/j.cub.2019.06.031.

ACKNOWLEDGMENTS

We thank Dr. M. Sheng and J. Newman for their comments on the manuscript. We are grateful to the MRC LMB microscopy facility and media center for expertise and assistance, the CRUK Genomics Core for sequencing services, and the Dillin lab, UC Berkeley, for provision of strains. Some strains were also provided by the CGC, which is funded by NIH Office of Research Infrastructure Programs (P40 OD010440). This work was supported by the Medical Research Council.

AUTHOR CONTRIBUTIONS

S.I. and R.C.T. conceived, performed, and analyzed experiments and wrote the manuscript. N.P.O. developed methodology, performed and analyzed experiments, and curated data. C.K. provided expertise and analyzed data. M.O.C. provided expertise and resources and reviewed the manuscript.

DECLARATION OF INTERESTS

The authors declare no competing interests.

Received: July 27, 2018
Revised: May 2, 2019
Accepted: June 10, 2019
Published: July 11, 2019

REFERENCES

1. Apfeld, J., and Kenyon, C. (1999). Regulation of lifespan by sensory perception in Caenorhabditis elegans. Nature *402*, 804–809.
2. Libert, S., Zwiener, J., Chu, X., Vanvoorhies, W., Roman, G., and Pletcher, S.D. (2007). Regulation of Drosophila life span by olfaction and food-derived odors. Science *315*, 1133–1137.
3. Riera, C.E., Huising, M.O., Follett, P., Leblanc, M., Halloran, J., Van Andel, R., de Magalhaes Filho, C.D., Merkwirth, C., and Dillin, A. (2014). TRPV1 pain receptors regulate longevity and metabolism by neuropeptide signaling. Cell *157*, 1023–1036.
4. Taylor, R.C., Berendzen, K.M., and Dillin, A. (2014). Systemic stress signalling: understanding the cell non-autonomous control of proteostasis. Nat. Rev. Mol. Cell Biol. *15*, 211–217.

5. Ron, D., and Walter, P. (2007). Signal integration in the endoplasmic reticulum unfolded protein response. Nat. Rev. Mol. Cell Biol. *8*, 519–529.

6. Taylor, R.C., and Dillin, A. (2013). XBP-1 is a cell-nonautonomous regulator of stress resistance and longevity. Cell *153*, 1435–1447.

7. Ben-Zvi, A., Miller, E.A., and Morimoto, R.I. (2009). Collapse of proteostasis represents an early molecular event in Caenorhabditis elegans aging. Proc. Natl. Acad. Sci. USA *106*, 14914–14919.

8. Morley, J.F., Brignull, H.R., Weyers, J.J., and Morimoto, R.I. (2002). The threshold for polyglutamine-expansion protein aggregation and cellular toxicity is dynamic and influenced by aging in Caenorhabditis elegans. Proc. Natl. Acad. Sci. USA *99*, 10417–10422.

9. Taylor, R.C., and Dillin, A. (2011). Aging as an event of proteostasis collapse. Cold Spring Harb. Perspect. Biol. *3*, 3.

10. Garcia, S.M., Casanueva, M.O., Silva, M.C., Amaral, M.D., and Morimoto, R.I. (2007). Neuronal signaling modulates protein homeostasis in Caenorhabditis elegans post-synaptic muscle cells. Genes Dev. *21*, 3006–3016.

11. Prahlad, V., and Morimoto, R.I. (2011). Neuronal circuitry regulates the response of Caenorhabditis elegans to misfolded proteins. Proc. Natl. Acad. Sci. USA *108*, 14204–14209.

12. Morley, J.F., and Morimoto, R.I. (2004). Regulation of longevity in Caenorhabditis elegans by heat shock factor and molecular chaperones. Mol. Biol. Cell *15*, 657–664.

13. Wu, Y., Wu, Z., Butko, P., Christen, Y., Lambert, M.P., Klein, W.L., Link, C.D., and Luo, Y. (2006). Amyloid-beta-induced pathological behaviors are suppressed by Ginkgo biloba extract EGb 761 and ginkgolides in transgenic Caenorhabditis elegans. J. Neurosci. *26*, 13102–13113.

14. Brignull, H.R., Moore, F.E., Tang, S.J., and Morimoto, R.I. (2006). Polyglutamine proteins at the pathogenic threshold display neuron-specific aggregation in a pan-neuronal Caenorhabditis elegans model. J. Neurosci. *26*, 7597–7606.

15. Gidalevitz, T., Ben-Zvi, A., Ho, K.H., Brignull, H.R., and Morimoto, R.I. (2006). Progressive disruption of cellular protein folding in models of polyglutamine diseases. Science *311*, 1471–1474.

16. Cohen, E., Bieschke, J., Perciavalle, R.M., Kelly, J.W., and Dillin, A. (2006). Opposing activities protect against age-onset proteotoxicity. Science *313*, 1604–1610.

17. Link, C.D. (1995). Expression of human beta-amyloid peptide in transgenic Caenorhabditis elegans. Proc. Natl. Acad. Sci. USA *92*, 9368–9372.

18. McColl, G., Roberts, B.R., Gunn, A.P., Perez, K.A., Tew, D.J., Masters, C.L., Barnham, K.J., Cherny, R.A., and Bush, A.I. (2009). The Caenorhabditis elegans A beta 1-42 model of Alzheimer disease predominantly expresses A beta 3-42. J. Biol. Chem. *284*, 22697–22702.

19. Mohri-Shiomi, A., and Garsin, D.A. (2008). Insulin signaling and the heat shock response modulate protein homeostasis in the Caenorhabditis elegans intestine during infection. J. Biol. Chem. *283*, 194–201.

20. Kaletsky, R., Lakhina, V., Arey, R., Williams, A., Landis, J., Ashraf, J., and Murphy, C.T. (2016). The C. elegans adult neuronal IIS/FOXO transcriptome reveals adult phenotype regulators. Nature *529*, 92–96.

21. Imanikia, S., Galea, F., Nagy, E., Phillips, D.H., Stürzenbaum, S.R., and Arlt, V.M. (2016). The application of the comet assay to assess the genotoxicity of environmental pollutants in the nematode Caenorhabditis elegans. Environ. Toxicol. Pharmacol. *45*, 356–361.

22. Benedetti, C., Haynes, C.M., Yang, Y., Harding, H.P., and Ron, D. (2006). Ubiquitin-like protein 5 positively regulates chaperone gene expression in the mitochondrial unfolded protein response. Genetics *174*, 229–239.

23. Huber, L.A., and Teis, D. (2016). Lysosomal signaling in control of degradation pathways. Curr. Opin. Cell Biol. *39*, 8–14.

24. Tabara, H., Sarkissian, M., Kelly, W.G., Fleenor, J., Grishok, A., Timmons, L., Fire, A., and Mello, C.C. (1999). The rde-1 gene, RNA interference, and transposon silencing in C. elegans. Cell *99*, 123–132.

25. Espelt, M.V., Estevez, A.Y., Yin, X., and Strange, K. (2005). Oscillatory Ca2+ signaling in the isolated Caenorhabditis elegans intestine: role of the inositol-1,4,5-trisphosphate receptor and phospholipases C beta and gamma. J. Gen. Physiol. *126*, 379–392.

26. Marré, J., Traver, E.C., and Jose, A.M. (2016). Extracellular RNA is transported from one generation to the next in Caenorhabditis elegans. Proc. Natl. Acad. Sci. USA *113*, 12496–12501.

27. Lapierre, L.R., De Magalhaes Filho, C.D., McQuary, P.R., Chu, C.C., Visvikis, O., Chang, J.T., Gelino, S., Ong, B., Davis, A.E., Irazoqui, J.E., et al. (2013). The TFEB orthologue HLH-30 regulates autophagy and modulates longevity in Caenorhabditis elegans. Nat. Commun. *4*, 2267.

28. O'Rourke, E.J., and Ruvkun, G. (2013). MXL-3 and HLH-30 transcriptionally link lipolysis and autophagy to nutrient availability. Nat. Cell Biol. *15*, 668–676.

29. Treusch, S., Knuth, S., Slaugenhaupt, S.A., Goldin, E., Grant, B.D., and Fares, H. (2004). Caenorhabditis elegans functional orthologue of human protein h-mucolipin-1 is required for lysosome biogenesis. Proc. Natl. Acad. Sci. USA *101*, 4483–4488.

30. Kang, C., You, Y.J., and Avery, L. (2007). Dual roles of autophagy in the survival of Caenorhabditis elegans during starvation. Genes Dev. *21*, 2161–2171.

31. Chang, J.T., Kumsta, C., Hellman, A.B., Adams, L.M., and Hansen, M. (2017). Spatiotemporal regulation of autophagy during *Caenorhabditis elegans* aging. eLife. Published online July 4, 2017. https://doi.org/10.7554/eLife.18459.

32. Baxi, K., and de Carvalho, C.E. (2018). Assessing lysosomal alkalinization in the intestine of live Caenorhabditis elegans. J. Vis. Exp. *134*, e57414.

33. Gelino, S., Chang, J.T., Kumsta, C., She, X., Davis, A., Nguyen, C., Panowski, S., and Hansen, M. (2016). Intestinal autophagy improves healthspan and longevity in C. elegans during dietary restriction. PLoS Genet. *12*, e1006135.

34. Settembre, C., Fraldi, A., Medina, D.L., and Ballabio, A. (2013). Signals from the lysosome: a control centre for cellular clearance and energy metabolism. Nat. Rev. Mol. Cell Biol. *14*, 283–296.

35. Ramirez, A., Heimbach, A., Gründemann, J., Stiller, B., Hampshire, D., Cid, L.P., Goebel, I., Mubaidin, A.F., Wriekat, A.L., Roeper, J., et al. (2006). Hereditary parkinsonism with dementia is caused by mutations in ATP13A2, encoding a lysosomal type 5 P-type ATPase. Nat. Genet. *38*, 1184–1191.

36. Usenovic, M., and Krainc, D. (2012). Lysosomal dysfunction in neurodegeneration: the role of ATP13A2/PARK9. Autophagy *8*, 987–988.

37. Lee, J.H., Yu, W.H., Kumar, A., Lee, S., Mohan, P.S., Peterhoff, C.M., Wolfe, D.M., Martinez-Vicente, M., Massey, A.C., Sovak, G., et al. (2010). Lysosomal proteolysis and autophagy require presenilin 1 and are disrupted by Alzheimer-related PS1 mutations. Cell *141*, 1146–1158.

38. Coen, K., Flannagan, R.S., Baron, S., Carraro-Lacroix, L.R., Wang, D., Vermeire, W., Michiels, C., Munck, S., Baert, V., Sugita, S., et al. (2012). Lysosomal calcium homeostasis defects, not proton pump defects, cause endo-lysosomal dysfunction in PSEN-deficient cells. J. Cell Biol. *198*, 23–35.

39. Kim, D.K., Lim, H.S., Kawasaki, I., Shim, Y.H., Vaikath, N.N., El-Agnaf, O.M., Lee, H.J., and Lee, S.J. (2016). Anti-aging treatments slow propagation of synucleinopathy by restoring lysosomal function. Autophagy *12*, 1849–1863.

40. Medina, D.L., Fraldi, A., Bouche, V., Annunziata, F., Mansueto, G., Spampanato, C., Puri, C., Pignata, A., Martina, J.A., Sardiello, M., et al. (2011). Transcriptional activation of lysosomal exocytosis promotes cellular clearance. Dev. Cell *21*, 421–430.

41. Spampanato, C., Feeney, E., Li, L., Cardone, M., Lim, J.A., Annunziata, F., Zare, H., Polishchuk, R., Puertollano, R., Parenti, G., et al. (2013). Transcription factor EB (TFEB) is a new therapeutic target for Pompe disease. EMBO Mol. Med. *5*, 691–706.

42. Tsunemi, T., Ashe, T.D., Morrison, B.E., Soriano, K.R., Au, J., Roque, R.A., Lazarowski, E.R., Damian, V.A., Masliah, E., and La Spada, A.R. (2012). PGC-1α rescues Huntington's disease proteotoxicity by preventing oxidative stress and promoting TFEB function. Sci. Transl. Med. *4*, 142ra97.

43. Pastore, N., Blomenkamp, K., Annunziata, F., Piccolo, P., Mithbaokar, P., Maria Sepe, R., Vetrini, F., Palmer, D., Ng, P., Polishchuk, E., et al. (2013). Gene transfer of master autophagy regulator TFEB results in clearance of toxic protein and correction of hepatic disease in alpha-1-anti-trypsin deficiency. EMBO Mol. Med. *5*, 397–412.
44. Hetz, C., and Mollereau, B. (2014). Disturbance of endoplasmic reticulum proteostasis in neurodegenerative diseases. Nat. Rev. Neurosci. *15*, 233–249.
45. Cohen, E., Paulsson, J.F., Blinder, P., Burstyn-Cohen, T., Du, D., Estepa, G., Adame, A., Pham, H.M., Holzenberger, M., Kelly, J.W., et al. (2009). Reduced IGF-1 signaling delays age-associated proteotoxicity in mice. Cell *139*, 1157–1169.
46. Ross, C.A., and Poirier, M.A. (2005). Opinion: What is the role of protein aggregation in neurodegeneration? Nat. Rev. Mol. Cell Biol. *6*, 891–898.
47. Sancak, Y., Bar-Peled, L., Zoncu, R., Markhard, A.L., Nada, S., and Sabatini, D.M. (2010). Ragulator-Rag complex targets mTORC1 to the lysosomal surface and is necessary for its activation by amino acids. Cell *141*, 290–303.
48. Chieregatti, E., and Meldolesi, J. (2005). Regulated exocytosis: new organelles for non-secretory purposes. Nat. Rev. Mol. Cell Biol. *6*, 181–187.
49. Stinchcombe, J.C., and Griffiths, G.M. (2007). Secretory mechanisms in cell-mediated cytotoxicity. Annu. Rev. Cell Dev. Biol. *23*, 495–517.
50. Stinchcombe, J., Bossi, G., and Griffiths, G.M. (2004). Linking albinism and immunity: the secrets of secretory lysosomes. Science *305*, 55–59.
51. Folick, A., Oakley, H.D., Yu, Y., Armstrong, E.H., Kumari, M., Sanor, L., Moore, D.D., Ortlund, E.A., Zechner, R., and Wang, M.C. (2015). Aging. Lysosomal signaling molecules regulate longevity in Caenorhabditis elegans. Science *347*, 83–86.
52. Menzies, F.M., Fleming, A., Caricasole, A., Bento, C.F., Andrews, S.P., Ashkenazi, A., Füllgrabe, J., Jackson, A., Jimenez Sanchez, M., Karabiyik, C., et al. (2017). Autophagy and neurodegeneration: pathogenic mechanisms and therapeutic opportunities. Neuron *93*, 1015–1034.
53. Rubinsztein, D.C., Mariño, G., and Kroemer, G. (2011). Autophagy and aging. Cell *146*, 682–695.
54. Bohnert, K.A., and Kenyon, C. (2017). A lysosomal switch triggers proteostasis renewal in the immortal C. elegans germ lineage. Nature *551*, 629–633.
55. Hughes, A.L., and Gottschling, D.E. (2012). An early age increase in vacuolar pH limits mitochondrial function and lifespan in yeast. Nature *492*, 261–265.
56. Brenner, S. (1974). The genetics of *Caenorhabditis elegans*. Genetics *77*, 71–94.
57. Kamath, R.S., Martinez-Campos, M., Zipperlen, P., Fraser, A.G., and Ahringer, J. (2000). Effectiveness of specific RNA-mediated interference through ingested double-stranded RNA in Caenorhabditis elegans. Genome Biol. *2*, research0002.0001.
58. Hart, A.C. (2006). Behavior. In WormBook, The C. elegans, Research Community (WormBook). https://doi.org/10.1895/wormbook.1.87.1. http://www.wormbook.org.
59. Duerr, J.S. (2006). Immunohistochemistry. In WormBook, The C. elegans, Research Community (WormBook). https://doi.org/10.1895/wormbook.1.105.1. http://www.wormbook.org.
60. Shaham, S. (2006). Methods in cell biology. In WormBook, The C. elegans, Research Community (WormBook). https://doi.org/10.1895/wormbook.1.49.1. http://www.wormbook.org.
61. Gidalevitz, T., Krupinski, T., Garcia, S., and Morimoto, R.I. (2009). Destabilizing protein polymorphisms in the genetic background direct phenotypic expression of mutant SOD1 toxicity. PLoS Genet. *5*, e1000399.
62. Miller, L. (2010). Analyzing gels and western blots with ImageJ, *Volume 2018*.

STAR★METHODS

KEY RESOURCES TABLE

REAGENT or RESOURCE	SOURCE	IDENTIFIER
Antibodies		
α-Aβ	BioLegend	6E10; Cat#803001; RRID: AB_2564653
α-polyQ	Sigma Aldrich	3B5H10; Cat#MABN821; RRID: AB_532270
α-GFP/YFP	ChromoTek	3H9; Cat#3h9-100; RRID: AB_10773374
α-α-tubulin	Sigma Aldrich	B512; Cat#T5168; RRID: AB_477579
Bacterial and Virus Strains		
E. coli OP50	CGC	WB OP50; RRID: WB-STRAIN:OP50
E. coli HT115	CGC	WB HT115; RRID: WB-STRAIN:HT115
L4440 RNAi	Addgene	Cat#1654
bec-1 RNAi	Vidal ORF-RNAi library, Source Bioscience	Cat#3320_Cel_ORF_RNAi
vps-34 RNAi	Vidal ORF-RNAi library, Source Bioscience	Cat#3320_Cel_ORF_RNAi
xbp-1 RNAi	Vidal ORF-RNAi library, Source Bioscience	Cat#3320_Cel_ORF_RNAi
asp-3 RNAi	Ahringer library, Source Bioscience	Cat#3318_Cel_RNAi_complete
atg-13 RNAi	Ahringer library, Source Bioscience	Cat#3318_Cel_RNAi_complete
atg-18 RNAi	Ahringer library, Source Bioscience	Cat#3318_Cel_RNAi_complete
cup-5 RNAi	Ahringer library, Source Bioscience	Cat#3318_Cel_RNAi_complete
hlh-30 RNAi	Ahringer library, Source Bioscience	Cat#3318_Cel_RNAi_complete
lmp-1 RNAi	Ahringer library, Source Bioscience	Cat#3318_Cel_RNAi_complete
vha-15 RNAi	Ahringer library, Source Bioscience	Cat#3318_Cel_RNAi_complete
vha-18 RNAi	Ahringer library, Source Bioscience	Cat#3318_Cel_RNAi_complete
vha-2 RNAi	Ahringer library, Source Bioscience	Cat#3318_Cel_RNAi_complete
vha-8 RNAi	Ahringer library, Source Bioscience	Cat#3318_Cel_RNAi_complete
daf-2 RNAi	Dillin lab, UC Berkeley	N/A
daf-16 RNAi	Dillin lab, UC Berkeley	N/A
Chemicals, Peptides, and Recombinant Proteins		
Carbenicillin	Formedium	Cat#CAR0025
IPTG	Generon	Cat#orb340528
Sodium azide	Sigma Aldrich	Cat#S2002
Levamisole	Sigma Aldrich	Cat#L0380000
Tetramisole	Sigma Aldrich	Cat#T1512-5G
Benzaldehyde	Sigma Aldrich	Cat#B1334-250ML
AlexaFluor 546-Phalloidin	Life Technologies Ltd.	Cat#A22283
Papain	AppliChem GmbH	Cat#A3824
Fetal bovine serum	GIBCO, Life Technologies	Cat#10270106
Trizol LS	Ambion, Life Technologies	Cat#10296010
SYBRGreen Master Mix	Applied Biosystems	Cat#4472897
cDCFDA	ThermoFisher	Cat#C369
LysoTracker DeepRed	ThermoFisher	Cat#L12492
Critical Commercial Assays		
Pierce BCA protein assay kit	Thermo Scientific	Cat#23227
Direct-zol kit	Zymo Research	Cat#R2060
NuGEN Ovation RNaseq v2 kit	NuGEN	Cat#7102
Agilent High Sensitivity DNA kit	Agilent	Cat#5067-4626
Ovation Ultralow System v2 kit	NuGEN	Cat#0344

(*Continued on next page*)

Continued

REAGENT or RESOURCE	SOURCE	IDENTIFIER
KAPA Library Quantification Kit	Illumina Platforms	Cat#07960140001
Agilent RNA 6000 Pico Kit	Agilent	Cat#5067-1513
QuantiTect reverse transcription kit	QIAGEN	Cat#205310
Fluorometric assay kit	Abcam	Cat#ab65300
Deposited Data		
NCBI	BioProject	PRJNA482604
C. elegans strains		
Wild type, Bristol	CGC [55]	N2
uthIs270[*rab-3p::xbp-1s, myo-2p::tdTomato*]	CGC [6]	AGD927
dvIs50[*snb-1::Aβ1-42, mtl-2::GFP*]	CGC [13]	CL2355
dyn-1(ky51)	CGC [7]	CX51
let-60(ga89)	CGC [7]	SD551
rmIs132[*unc-54p::Q35::YFP*]	CGC [8]	AM140
dvIs2[*unc-54p::Aβ1-42, rol-6(su1006)]*	CGC [17]	CL2006
unc-15(e1402)	CGC [7]	CB1402
rde-1(ne219)	CGC [24]	WM27
rde-1(ne219); kbIs7 [*nhx-2p::rde-1* + *rol-6(su1006)*]	CGC [25]	VP303
rde-1(ne219); jamSi2 [*mex-5p::rde-1*]	CGC [26]	AMJ345
pwIs50 [*lmp-1p::lmp-1::GFP, Cbr-unc-119(+)])*	CGC [29]	RT258
adIs2122[*lgg-1p::GFP::lgg-1, rol-6(su1006)*]	CGC [30]	DA2123
sqIs11[*lgg-1p::mCherry::GFP::lgg-1+rol-6*]	CGC [31]	MAH215
[sqIs17 [*hlh-30p::hlh-30*::GFP + *rol-6*]	CGC [27]	MAH240
rmIs110[*rgef-1p::Q40::YFP*]	Dillin lab (outcrossed from [14])	AGD1397
uthIs393[*vha-6p::Q40::YFP*, *rol-6(su1006)*]	Dillin lab (integrated from [19])	AGD1395
uthIs388[*gly-19p::xbp-1s, myo-2p::tdTomato*]	This paper (integrated from [6])	AGD1379
uthIs390[*unc-54p::xbp-1s, myo-2p::tdTomato*]	This paper (integrated from [6])	AGD1391
zcIs18(*ges-1p::GFP*)	This paper (outcrossed from [22])	RCT51
uthIs270[*rab-3p::xbp-1s, myo-2p::tdTomato*]; dvIs2[*unc-54p::Aβ1-42, rol-6(su1006)]*	This paper	RCT1
uthIs388[*gly-19p::xbp-1s, myo-2p::tdTomato*]; dvIs2[*unc-54p::Aβ1-42, rol-6(su1006)]*	This paper	RCT2
uthIs390[*unc-54p::xbp-1s, myo-2p::tdTomato*]; dvIs2[*unc-54p::Aβ1-42, rol-6(su1006)]*	This paper	RCT3
uthIs270[*rab-3p::xbp-1s, myo-2p::tdTomato*]; dvIs50[*snb-1::Aβ1-42, mtl-2::GFP*]	This paper	RCT23
uthIs388[*gly-19p::xbp-1s, myo-2p::tdTomato*]; dvIs50[*snb-1::Aβ1-42, mtl-2::GFP*]	This paper	RCT25
uthIs270[*rab-3p::xbp-1s, myo-2p::tdTomato*]; rmIs132[*unc-54p::Q35::YFP*]	This paper	AGD1412
rmsEx15(*gly-19p::xbp-1s, myo-2p::tdTomato*); rmIs132[*unc-54p::Q35::YFP*]	This paper	RCT146
uthIs390[*unc-54p::xbp-1s, myo-2p*::tdTomato]; rmIs132[*unc-54p::Q35::YFP*]	This paper	RCT4
uthIs270[*rab-3p::xbp-1s, myo-2p::tdTomato*]; rmIs110[*rgef-1p::Q40::YFP*]	This paper	AGD1399
uthIs388[*gly-19p::xbp-1s*, *myo-2p::tdTomato*]; rmIs110[*rgef-1p::Q40::YFP*]	This paper	RCT31
uthIs390[*unc-54p::xbp-1s, myo-2p*::tdTomato]; rmIs110[*rgef-1p::Q40::YFP*]	This paper	RCT32
uthIs270[*rab-3p::xbp-1s, myo-2p::tdTomato*]; uthIs393[*vha-6p::Q40::YFP, rol-6(su1006)*]	This paper	RCT33

(*Continued on next page*)

Continued

REAGENT or RESOURCE	SOURCE	IDENTIFIER
uthIs388[*gly-19p::xbp-1s*, *myo-2p::tdTomato*]; uthIs393[*vha-6p::Q40::YFP*, *rol-6(su1006)*]	This paper	RCT26
uthIs270[*rab-3p::xbp-1s*, *myo-2p::tdTomato*]; *dyn-1(ky51)*	This paper	RCT59
uthIs270[*rab-3p::xbp-1s*, *myo-2p::tdTomato*]; *unc-15(e1402)*	This paper	RCT60
uthIs270[*rab-3p::xbp-1s*, *myo-2p::tdTomato*]; *let-60(ga89)*	This paper	RCT112
uthIs270[*rab-3p::xbp-1s, myo-2p::tdTomato*]; zcIs18(*ges-1p::GFP*)	This paper	RCT52
uthIs270[*rab-3p::xbp-1s*, *myo-2p::tdTomato*]; *rde-1(ne219);* kbIs7 [*nhx-2p::rde-1* + *rol-6(su1006)*]	This paper	RCT145
uthIs270[*rab-3p::xbp-1s*, *myo-2p::tdTomato*]; *rde-1(ne219);* jamSi2 [*mex-5p::rde-1*]	This paper	RCT105
dvIs50[*snb-1::Aβ1-42, mtl-2::GFP*]; *rde-1(ne219);* jamSi2 [*mex-5p::rde-1*]	This paper	RCT109
uthIs270[*rab-3p::xbp-1s*, *myo-2p::tdTomato*]; dvIs50[*snb-1::Aβ1-42, mtl-2::GFP*]; *rde-1(ne219);* jamSi2 [*mex-5p::rde-1*]	This paper	RCT110
uthIs388[*gly-19p::xbp-1s*, *myo-2p::tdTomato*]; dvIs50[*snb-1::Aβ1-42, mtl-2::GFP*]; *rde-1(ne219);* jamSi2 [*mex-5p::rde-1*]	This paper	RCT111
rmIs110[*rgef-1p::Q40::YFP*]); *rde-1(ne219);* jamSi2 [*mex-5p::rde-1*]	This paper	RCT106
uthIs270[*rab-3p::xbp-1s*, *myo-2p::tdTomato*]; rmIs110[*rgef-1p::Q40::YFP*]); *rde-1(ne219);* jamSi2 [*mex-5p::rde-1*]	This paper	RCT107
uthIs388[*gly-19p::xbp-1s*, *myo-2p::tdTomato*]; rmIs110[*rgef-1p::Q40::YFP*]); *rde-1(ne219);* jamSi2 [*mex-5p::rde-1*]	This paper	RCT108
uthIs270[*rab-3p::xbp-1s*, *myo-2p::tdTomato*]; sqIs17 [*hlh-30p::hlh-30*::GFP + *rol-6*]	This paper	RCT94
uthIs270 [*rab-3p::xbp-1s*, *myo-2p::tdTomato*]; pwIs50 [*lmp-1p::lmp-1::GFP*, *Cbr-unc-119(+)*]	This paper	RCT103
uthIs270[*rab-3p::xbp-1s*, *myo-2p::tdTomato*]; adIs2122[*lgg-1p::GFP::lgg-1, rol-6(su1006)*]	This paper	RCT104
uthIs270[*rab-3p::xbp-1s*, *myo-2p::tdTomato*]; sqIs11[*lgg-1p::mCherry::GFP::lgg-1+ rol-6*]	This paper	RCT98
Oligonucleotides		
See Table S2	N/A	N/A
Software and Algorithms		
SeqMonk	Babraham Institute	https://www.bioinformatics.babraham.ac.uk/projects/seqmonk/
g:Profiler	ELIXIR	https://biit.cs.ut.ee/gprofiler/index.cgi
Promoterome Database	Harvard University	http://worfdb.dfci.harvard.edu/promoteromedb/

LEAD CONTACT AND MATERIALS AVAILABILITY

Further information and requests for resources and reagents should be directed to and will be fulfilled by the Lead Contact, Rebecca Taylor (rtaylor@mrc-lmb.cam.ac.uk).

EXPERIMENTAL MODEL AND SUBJECT DETAILS

C. elegans maintenance and RNAi knockdown

C. elegans strains were maintained at 20°C on nematode growth medium (NGM) plates seeded with OP50 bacteria unless otherwise stated [56]. For feeding RNAi experiments either L4440 empty vector or the designated RNAi bacteria was used [57]. Plates for RNAi analysis were prepared by supplementation of agar with 100 μg/mL carbenicillin and 1mM IPTG after autoclaving. 24 hours prior to each assay plates were spotted with 100 μL of overnight bacterial culture. Where dual RNAi knockdown was performed, equal volumes of two cultures normalized to equivalent OD_{600} were mixed before plates were seeded.

METHOD DETAILS

Chemotaxis assays

Animals were synchronized by timed egg lay on 55 mm NGM plates. Chemotaxis assays were conducted at day 1 of adulthood as described (section 4.4 of [58]). Briefly, animals were raised on OP50 or designated RNAi bacteria until day 1 of adulthood, when they were collected and washed three times using M9 buffer, with worms settled between washes by gravity. Assay plates were prepared using a 1 μL spot of ethanol (solvent spot) and a 1 μL spot of chemo-attractant, 1:100 benzaldehyde (target spot) on opposite sides. 1 μL of sodium azide (50mM) was added to solvent and target spots once dried. Worms were dispensed at the center of the plate and animals kept in the dark for 60 min at room temperature before worms at each spot were counted. Chemotaxis indices were then calculated as (# worms at target spot - # worms at vehicle spot)/total number of worms on the plate. Indices were reported as −1 to 1, where 1 indicates that 100% of animals have arrived at the target spot. All assays were repeated at least 3 times, and significance was assessed by one-way ANOVA with Tukey's multiple comparisons test.

Paralysis assays

Eggs were isolated by bleaching and allowed to develop on a bacterial lawn. At the L4 larval stage, individual nematodes were picked and transferred to 55mm NGM plates seeded with OP50. Paralysis was evaluated daily from day 1 to day 12 of adulthood and scored by touching the animal's nose with a platinum wire. Worms able to move the head but not the rest of the body were scored as paralyzed [16]. Dead animals or those with other phenotypes (e.g., vivipary) were censored from the analysis. All assays were performed with at least 100 animals and repeated at least 3 times.

Osmoregulatory assays

Age-matched animals were grown at 20°C. At day 1 of adulthood, worms were placed into a drop of distilled water for 5 minutes, after which animals with a swollen, fluid-filled shape were scored as osmoregulation defective (Osm), as described [15]. All assays were replicated 3 times and significance was assessed by two-way ANOVA with Tukey's multiple comparison.

Motility assays

Animals were synchronized by timed egg lay and grown to adulthood on OP50 or RNAi bacteria. 10-15 day 1 or day 4 adult worms of each genotype were transferred to a 24-well dish containing 700 μL of M9 buffer and allowed to settle for 1 minute, after which each well was filmed for 30 s at a frame rate of 15 fps. Movies were analyzed manually to assess thrashing rate. Each assay was repeated 3 times and significance assessed by one-way ANOVA with Tukey's multiple comparisons test.

F-actin staining

Synchronized worms were freeze cracked to permeabilize the cuticle at day 5 of adulthood. Staining was performed using AlexaFluor 546-Phalloidin to visualize F-actin in body wall muscles, as described (section 2.6 of [59]; section 2.3 of [60]) in 3 independent biological replicates.

Fluorescence microscopy

Micrographs of worms were acquired using a Leica M205 FA microscope and LAS X software. 5-10 animals were anesthetized using levamisole (25 mM) or sodium azide (50 mM) prior to imaging. All fluorescence microscopy analysis was independently replicated at least 3 times.

Confocal microscopy

For live microscopy nematodes were mounted on a 2% agarose pad and anesthetized using levamisole (25 mM). For HLH-30::GFP animals, sodium azide (50 mM) was used to avoid the effects of levamisole on nuclear localization of HLH-30. Fixed animals were prepared as described for F-actin staining. Images were acquired using a Zeiss LSM 710 confocal microscope using the 20x air, or 40x and 63x oil immersion objectives. A single section was acquired for all imaging and the pinhole used was 1 AU for optimal section thickness using the smart setup function. Image analysis utilized ImageJ or Fiji. For tandem-tagged nematodes, confocal microscopy was performed as described [31], where the Z-position was selected so that nuclei were clearly in focus in

order to visualize autolysosomes and autophagosomes. All confocal microscopy analysis was independently replicated at least 3 times.

Protein extraction and Western Blotting

For native extracts, nematodes were synchronized by bleaching and allowed to grow on designated bacterial strains until day 1 or 4 of adulthood. Worms were then washed three times with M9 buffer and pelleted before addition of 75-80 μL of native lysis buffer (as described in [61]). Samples were flash frozen in liquid nitrogen prior to use. Nematodes were thawed on ice and mechanically disrupted using a Precellys (Bertin Instruments) programmed for 3x15 s pulses, with 30 s between each pulse. Samples were then centrifuged for 5 minutes (8000 g) and supernatant containing total proteins was transferred to fresh tubes to resolve using NativePAGE 4%–16% Bis-Tris (Invitrogen). Gels were transferred using the iBlot 7-Minute Blotting System (Thermo Fisher Scientific) and imaged using ChemiDoc (BioRad). ImageLab software (BioRad) was further used to analyze the bands. For denaturing conditions, total cell lysate was prepared in RIPA buffer (with 2% SDS). Samples were centrifugated for 15min at high speed and pellets were resuspended in urea (7M) and treated with DTT. Samples were then boiled at 95°C for 5 minutes prior to loading on NuPAGE 4%–12% Bis Tris gels (Invitrogen). Gels were transferred and imaged as described above. For both native and denaturing conditions total protein concentrations were assayed using a Pierce BCA protein assay kit. All western blotting experiments were repeated at least 3 times.

Quantification of protein band intensity

Protein bands or lanes were quantified using ImageJ. The designated band or lane was selected and the band intensity calculated. Intensity was then normalized to the appropriate control and significance assessed by two-way ANOVA with Tukey's multiple comparisons test [62].

C. elegans cellular dissociation

Using animals with an intestinal GFP marker (RCT51 and RCT52), intestinal cells were isolated following the method described in [21]. Briefly, animals were harvested at day 1 of adulthood and washed several times to remove bacteria from their gut and surroundings. Cuticles were then permeabilized using a Triton X-100-SDS-DTT solution. For enzymatic digestion, papain (10 mg/mL) was used and animals were also mechanically disrupted using an electronic hand homogenizer (IKA T10 basic, ULTRA-TURRAX). Following this step, cells were recovered in fetal bovine serum (FBS) solution and kept on ice for sorting.

C. elegans cellular isolation by FACS

Intestinal cell suspensions (PBS/2% FBS) were passed through 35 and 40 mm nylon cell strainers (FALCON). Filtered cells were then diluted in the same media as above and sorted by a Sony iCyt Synergy Dual Channel, high speed cell sorter (488 nm excitation). Gates were used to eliminate cells with tdTomato and autofluorescence. GFP positive fluorescent events were collected in 1.5 mL eppendorf tubes containing 10-20 μL PBS/2% FBS. Cells were kept on ice for the RNA extraction step. Collected positive fluorescent events varied between 20,000 and 60,000 events for intestinal samples.

RNA extraction and amplification

FACS-sorted intestinal cells were centrifuged and cell volumes normalized to 100 μL. Immediately 400 μL of Trizol LS was added to the tubes which were then snap frozen in liquid nitrogen. RNA was extracted, DNase digested and cleaned using a microprep Direct-zol kit (Zymo Research). Initial RNA quantities for downstream analysis were normalized to 1.5 ng. Agilent Bioanalyser RNA Pico chips were used to assess the quantity and quality of RNA. Amplified cDNA was generated using the NuGEN Ovation RNaseq v2 kit. DNA quality and quantity were then evaluated using an Agilent High Sensitivity DNA kit and Nanodrop (Nanodrop 2000c, Thermo Scientifics). Thereafter, cDNA (1 μg) was sheared using a Covaris M220 sonicator (Covaris) to an average size of 150-200bp.

Library preparation, RNA sequencing and analysis

Library preparation was performed using the NuGEN Ovation Ultralow System v2 kit. Input cDNA was normalized to 90 ng. After purification, libraries were assessed using a Bioanalyzer High Sensitivity DNA chip. Fragment size was around 300 bp. Samples (10,000x and 20,000x dilutions) were quantified using the KAPA Library Quantification Kit. Samples were then pooled and quantified as before and further diluted to 15 nM for sequencing on an Illumina HiSeq4000 (CRUK CI, Cambridge). Reads were trimmed using Trim Galore (v0.4.5, cutadapt 1.15) and mapped to *C. elegans* WBcel235 using Hisat2 (v2.1.0). rRNA reads were removed and analysis performed using SeqMonk (https://www.bioinformatics.babraham.ac.uk/projects/seqmonk/). Differentially expressed genes were identified using an intensity difference filter with a cut-off point of $p < 0.05$. Gene ontology was analyzed using g:Profiler (https://biit.cs.ut.ee/gprofiler/index.cgi), with a custom-created background gene list based on the genes expressed in intestinal cells of *rab-3p::xbp-1s* worms, with $\log_2$ RPKM values between 0-100. Intestine-specific, muscle-specific and neuron-specific gene lists were obtained using the Princeton database of tissue-specific expression predictions for *C. elegans* (http://worm-tissue.princeton.edu/search/download), where only the genes with a score of 1 for the relevant tissue and a score of 0 for all the other tissues were chosen.

Quantitative RT-PCR

Total RNA was extracted as described above and 2 ng of purified RNA was used for cDNA synthesis using a QuantiTect reverse transcription kit. SYBRGreen quantitative RT-PCR was performed using either the Corbett system and following the Rotor-Gene 6000 Series Software manual, or a Vii7 Real-Time PCR (ThermoFisher Scientific). Data from 3 biological repeats were analyzed using the comparative 2ΔΔCt method. Significance was assessed by one-way ANOVA with Dunnett's multiple comparisons test.

Lifespan analysis

Lifespan analyses were performed at 20°C and were repeated at least twice. Typically, a minimum of 100 animals was used per condition, and worms were scored for viability every second day, from day 1 of adulthood (treating the pre-fertile day preceding adulthood as t = 0). Lifespans were performed on *E. coli* OP50, unless otherwise indicated, and animals were treated with 100 μg/mL FUDR at t = 0 and again at day 5 of adulthood, to avoid issues associated with early death through vivipary ("bagging") in *rab-3p::xbp-1s* animals. Prism 7 software was used for statistical analysis, and significance calculated using the log-rank (Mantel–Cox) method.

Quantification of lysosomes

RT258 and RCT103 worms expressing LMP-1::GFP were used to quantify the number of fluorescently-labeled lysosomes in the intestine of 10-15 animals per genotype from 3 independent biological replicates. ImageJ was used to analyze the data by counting numbers of LMP-1::GFP positive punctae. Fluorescence intensity of LMP-1::GFP punctae was also quantified at each time point and *xbp-1s*-expressing animals normalized to LMP-1::GFP alone. Quantification was performed using the oval-shaped selection tool to select the region of interest (ROI). GFP intensity was then quantified using the Measure function and background fluorescence intensity subtracted prior to normalization. Statistical analysis was carried out using a one-way ANOVA with Tukey's multiple comparisons test.

Quantification of autophagosomes

DA2123 and RCT104 worms expressing GFP::LGG-1 were used to quantify the number of autophagic vesicles in the intestine of 10-15 animals per genotype from 3 independent biological replicates. At days 2 and 5 of adulthood the total number of GFP::LGG-1 positive punctae was counted in the anterior section of the intestine. Data was analyzed using ImageJ and statistical analysis carried out using a one-way ANOVA with Tukey's multiple comparisons test

Lysosome acidity assays

To measure lysosomal acidity, the fluorescent pH-sensitive dye cDCFDA (5(6)-carboxy-2′,7'-dichlorofluorescein diacetate), which converts to cDCF (5-(6)-carboxy-2′,7'-dichlorofluorescein) through hydrolysis, was fed to live worms [32]. Animals were age-matched and grown on OP50 until day 1 or 4 of adulthood, then transferred to assay plates prepared by the addition of 100 μL of freshly prepared cDCFDA (10 mM) to a fresh lawn of OP50 bacteria in a 2cmx2cm patch. Plates were then kept in the dark to absorb dye into the agar/bacterial patch. Single worms picked onto assay plates were allowed to feed on the bacteria/cDCFDA patch for at least 16 hours prior to imaging the anterior section of the intestine, in 5-10 animals per genotype from 3 replicates. Data was analyzed using ImageJ, and statistical analysis carried out using a one-way ANOVA with Tukey's multiple comparisons test, similarly to the intensity quantification of LMP-1::GFP.

LysoTracker staining

C. elegans were grown from hatch to day 2 and 5 of adulthood on OP50- or RNAi bacteria-seeded plates containing 25 μM LysoTracker DeepRed or an equivalent volume of DMSO. Worms were then imaged by confocal microscopy, using 8-10 animals per genotype and 3 independent biological replicates; quantification was carried out using ImageJ, and statistical analysis conducted by one-way ANOVA with Tukey's multiple comparisons test.

Protease activity assays

Enzymatic activity of lysosomal proteases was determined using an Abcam fluorometric assay kit. Age-matched worms were grown on OP50 and lysed at day 2 and 5 of adulthood. Total proteins were extracted as described previously and protein concentration quantified and normalized. After adjusting volumes to 50 μL/well, 50 μL of reaction buffer was added per well followed by 2 μL of 10mM substrate (Ac-RR-AFC). Samples were incubated at 37°C for 1-2 hours and protease activity assessed using a fluorescent microplate reader (TECAN) at Ex/Em = 400/505 nm. All assays were prepared in triplicate and repeated 3 times independently, and statistical analysis carried out using a one-way ANOVA with Tukey's multiple comparisons test.

Tandem quantification of autophagosomes and autolysosomes

MAH215 worms expressing mCherry::GFP::LGG-1 were used to quantify the number of fluorescently-labeled autophagosomes and autolysosomes in the intestine of 10-15 animals per genotype from 3 independent biological replicates. ImageJ was used to analyze the data by counting numbers of mCherry::GFP positive autophagosomes, and mCherry positive autolysosomes. Statistical analysis was carried out using a one-way ANOVA with Tukey's multiple comparisons test.

QUANTIFICATION AND STATISTICAL ANALYSIS

Tests used to determine statistical significance were one-way ANOVA with Dunnett's multiple comparisons test (qRT-PCR); one-way ANOVA with Tukey's multiple comparison test (chemotaxis, motility and protease activity assays; quantification of lysosomes, autophagosomes, autolysosomes, lysosomal acidity, and Lysotracker staining); two-way ANOVA with Tukey's multiple comparisons test (osmoregulation assays and quantification of protein bands); and Mantel-Cox log-rank test (lifespan assays). Statistical information for each experiment can be found in the corresponding figure legend.

DATA AND CODE AVAILABILITY

The accession number for the RNA-seq data reported in this paper is NCBI BioProject number PRJNA482604.

Current Biology, Volume *29*

Supplemental Information

Neuronal XBP-1 Activates Intestinal Lysosomes to Improve Proteostasis in *C. elegans*

Soudabeh Imanikia, Neşem P. Özbey, Christel Krueger, M. Olivia Casanueva, and Rebecca C. Taylor

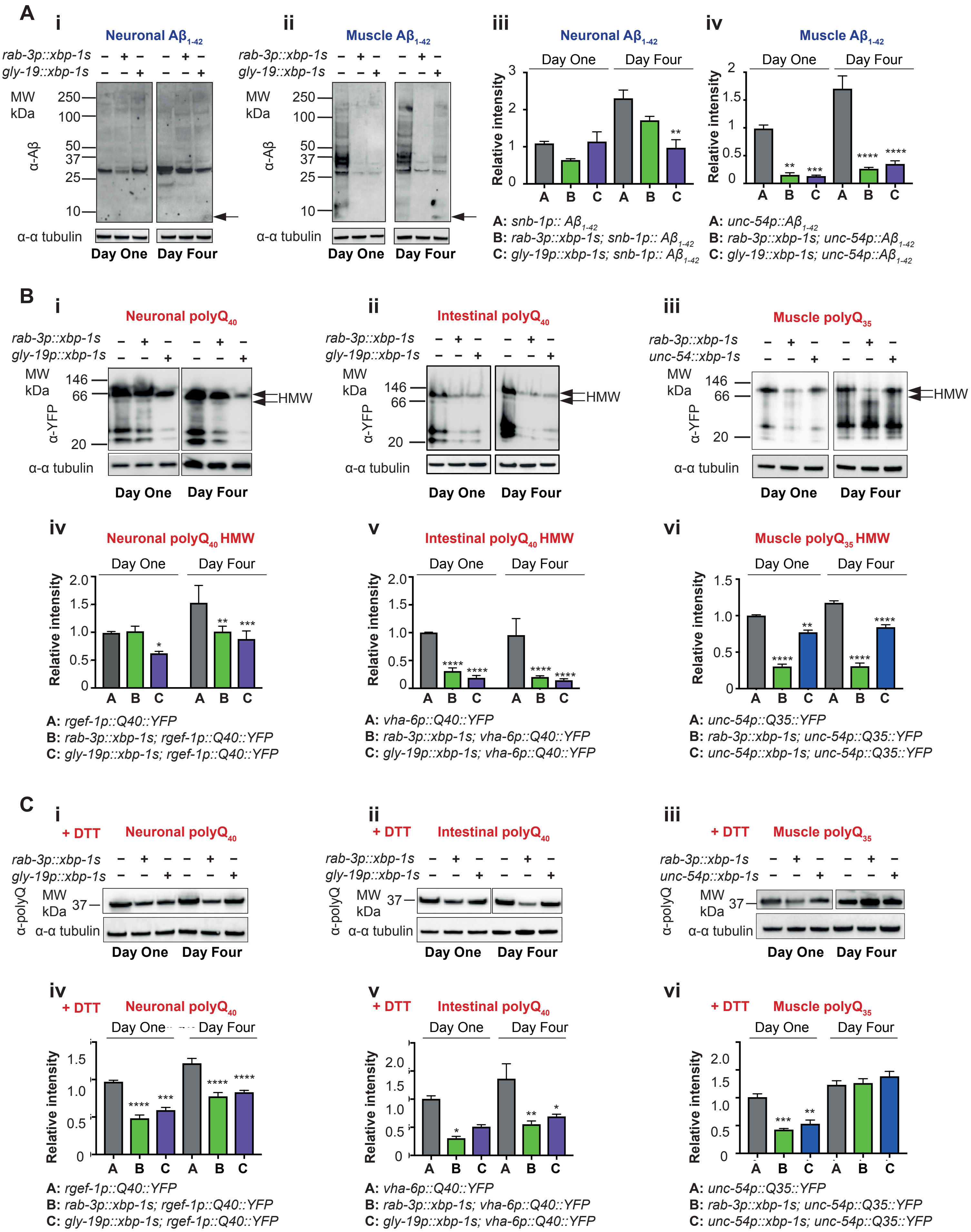
A
i
Neuronal Aβ1-42
rab-3p::xbp-1s – + – – + –
gly-19::xbp-1s – – + – – +
MW kDa
250
100
50
37
25
10
α-Aβ
α-α tubulin
Day One Day Four
ii
Muscle Aβ1-42
rab-3p::xbp-1s – + – – + –
gly-19::xbp-1s – – + – – +
MW kDa
250
100
50
37
25
10
α-Aβ
α-α tubulin
Day One Day Four
iii
Neuronal Aβ1-42
Day One Day Four
Relative intensity
3
2
1
0
**
A B C A B C
A: snb-1p:: Aβ1-42
B: rab-3p::xbp-1s; snb-1p:: Aβ1-42
C: gly-19p::xbp-1s; snb-1p:: Aβ1-42
iv
Muscle Aβ1-42
Day One Day Four
Relative intensity
2
1.5
1
0.5
0
** *** **** ****
A B C A B C
A: unc-54p::Aβ1-42
B: rab-3p::xbp-1s; unc-54p::Aβ1-42
C: gly-19::xbp-1s; unc-54p::Aβ1-42
B
i
Neuronal polyQ40
rab-3p::xbp-1s – + – – + –
gly-19p::xbp-1s – – + – – +
MW kDa
146
66
20
α-YFP
HMW
α-α tubulin
Day One Day Four
ii
Intestinal polyQ40
rab-3p::xbp-1s – + – – + –
gly-19p::xbp-1s – – + – – +
MW kDa
146
66
20
α-YFP
HMW
α-α tubulin
Day One Day Four
iii
Muscle polyQ35
rab-3p::xbp-1s – + – – + –
unc-54::xbp-1s – – + – – +
MW kDa
146
66
20
α-YFP
HMW
α-α tubulin
Day One Day Four
iv
Neuronal polyQ40 HMW
Day One Day Four
Relative intensity
2.0
1.5
1.0
0.5
0
* ** ***
A B C A B C
A: rgef-1p::Q40::YFP
B: rab-3p::xbp-1s; rgef-1p::Q40::YFP
C: gly-19p::xbp-1s; rgef-1p::Q40::YFP
v
Intestinal polyQ40 HMW
Day One Day Four
Relative intensity
2.0
1.5
1.0
0.5
0
**** **** **** ****
A B C A B C
A: vha-6p::Q40::YFP
B: rab-3p::xbp-1s; vha-6p::Q40::YFP
C: gly-19p::xbp-1s; vha-6p::Q40::YFP
vi
Muscle polyQ35 HMW
Day One Day Four
Relative intensity
1.5
1.0
0.5
0
**** ** **** ****
A B C A B C
A: unc-54p::Q35::YFP
B: rab-3p::xbp-1s; unc-54p::Q35::YFP
C: unc-54p::xbp-1s; unc-54p::Q35::YFP
C
i
+ DTT
Neuronal polyQ40
rab-3p::xbp-1s – + – – + –
gly-19p::xbp-1s – – + – – +
α-polyQ
MW kDa
37
α-α tubulin
Day One Day Four
ii
+ DTT
Intestinal polyQ40
rab-3p::xbp-1s – + – – + –
gly-19p::xbp-1s – – + – – +
α-polyQ
MW kDa
37
α-α tubulin
Day One Day Four
iii
+ DTT
Muscle polyQ35
rab-3p::xbp-1s – + – – + –
unc-54p::xbp-1s – – + – – +
α-polyQ
MW kDa
37
α-α tubulin
Day One Day Four
iv
+ DTT
Neuronal polyQ40
Day One Day Four
Relative intensity
1.5
1.0
0.5
0
**** *** **** ****
A B C A B C
A: rgef-1p::Q40::YFP
B: rab-3p::xbp-1s; rgef-1p::Q40::YFP
C: gly-19p::xbp-1s; rgef-1p::Q40::YFP
v
+ DTT
Intestinal polyQ40
Day One Day Four
Relative intensity
2.0
1.5
1.0
0.5
0
* ** *
A B C A B C
A: vha-6p::Q40::YFP
B: rab-3p::xbp-1s; vha-6p::Q40::YFP
C: gly-19p::xbp-1s; vha-6p::Q40::YFP
vi
+ DTT
Muscle polyQ35
Day One Day Four
Relative intensity
2.0
1.5
1.0
0.5
0
*** **
A B C A B C
A: unc-54p::Q35::YFP
B: rab-3p::xbp-1s; unc-54p::Q35::YFP
C: unc-54p::xbp-1s; unc-54p::Q35::YFP

Figure S1. Neuronal and intestinal *xbp-1s* reduce levels of toxic protein species. Related to Figure 2.

(A) Reducing Western blot analysis of (i) neuronal A$\beta_{1\text{-}42}$ and (ii) muscle A$\beta_{1\text{-}42}$ with and without neuronal and intestinal *xbp-1s,* at day 1 and day 4 of adulthood. Lysates were resolved under reducing conditions and blotted with an anti-Aβ antibody; the predicted MW of monomeric A$\beta_{1\text{-}42}$ is ~4.5 kDa, indicated with an arrow. Tubulin levels were probed with α-α tubulin as a loading control. Blots were quantified using ImageJ (iii-iv). Graphs represent mean lane intensity relative to day 1 A$\beta_{1\text{-}42}$ ± SD and statistical significance was calculated between A and B/C at each time point using two-way ANOVA with Tukey's multiple comparisons test, **p<0.01, ***p<0.001, ****p<0.0001. Data are representative of 3 independent experiments.

(B) Native Western blot analysis of (i) neuronal polyQ$_{40}$::YFP, (ii) intestinal polyQ$_{40}$::YFP and (iii) muscle polyQ$_{35}$::YFP with and without tissue-specific *xbp-1s,* at day 1 and day 4 of adulthood. Lysates were resolved under native conditions and blotted with an anti-YFP/GFP antibody. The predicted MW of monomeric polyQ$_{35\text{-}40}$:YFP is ~32 kDa – HMW indicates the higher molecular weight, polyQ-reactive band, while lower molecular weight, non-polyQ-reactive bands are likely to represent YFP cleaved from polyQ. Tubulin levels were probed with α-α tubulin as a loading control. Data are representative of at least 2 independent experiments. Blots were quantified using ImageJ (iv-vi). Bar graphs represent mean band intensity relative to day 1 polyQ ± standard deviation (SD) and statistical significance was calculated between A and B/C at each time point using two-way ANOVA with Tukey's multiple comparisons test, *p<0.05, **p<0.01, ***p<0.001, ****p<0.0001.

(C) Reducing Western blot analysis of (i) neuronal polyQ$_{40}$::YFP, (ii) intestinal polyQ$_{40}$::YFP and (iii) muscle polyQ$_{35}$::YFP expressed with and without tissue-specific *xbp-1s,* at day 1 and day 4 of adulthood. Lysates were resolved under reducing conditions and blotted with an anti-polyQ antibody; the predicted MW of monomeric polyQ$_{35\text{-}40}$:YFP is ~32 kDa. Tubulin levels were probed with α-α tubulin as a loading control. Blots were quantified using ImageJ (iv-vi). Bar graphs represent mean band intensity relative to day 1 polyQ ± SD and statistical significance was calculated between A and B/C at each time point using a two-way ANOVA with Tukey's multiple comparisons test, *$p<0.05$, **$p<0.01$, ***$p<0.001$, ****$p<0.0001$. Data are representative of at least 3 independent experiments.

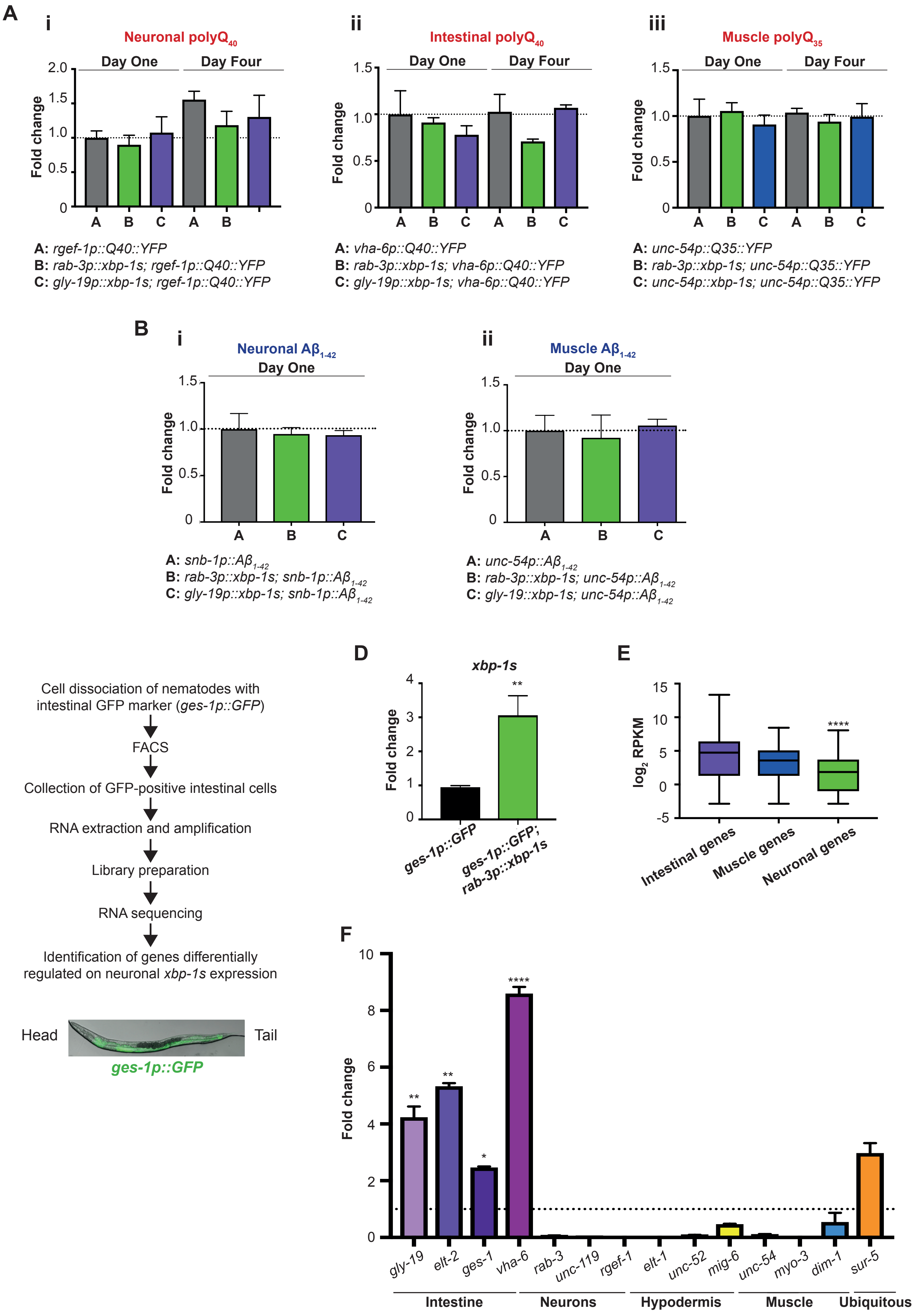
A
i
Neuronal polyQ40
Day One
Day Four
Fold change
A: rgef-1p::Q40::YFP
B: rab-3p::xbp-1s; rgef-1p::Q40::YFP
C: gly-19p::xbp-1s; rgef-1p::Q40::YFP
ii
Intestinal polyQ40
Day One
Day Four
Fold change
A: vha-6p::Q40::YFP
B: rab-3p::xbp-1s; vha-6p::Q40::YFP
C: gly-19p::xbp-1s; vha-6p::Q40::YFP
iii
Muscle polyQ35
Day One
Day Four
Fold change
A: unc-54p::Q35::YFP
B: rab-3p::xbp-1s; unc-54p::Q35::YFP
C: unc-54p::xbp-1s; unc-54p::Q35::YFP
B
i
Neuronal Aβ1-42
Day One
Fold change
A: snb-1p::Aβ1-42
B: rab-3p::xbp-1s; snb-1p::Aβ1-42
C: gly-19p::xbp-1s; snb-1p::Aβ1-42
ii
Muscle Aβ1-42
Day One
Fold change
A: unc-54p::Aβ1-42
B: rab-3p::xbp-1s; unc-54p::Aβ1-42
C: gly-19::xbp-1s; unc-54p::Aβ1-42
C
Cell dissociation of nematodes with intestinal GFP marker (ges-1p::GFP)
FACS
Collection of GFP-positive intestinal cells
RNA extraction and amplification
Library preparation
RNA sequencing
Identification of genes differentially regulated on neuronal xbp-1s expression
Head
Tail
ges-1p::GFP
D
xbp-1s
Fold change
ges-1p::GFP
ges-1p::GFP; rab-3p::xbp-1s
E
log2 RPKM
Intestinal genes
Muscle genes
Neuronal genes
F
Fold change
gly-19
elt-2
ges-1
vha-6
rab-3
unc-119
rgef-1
elt-1
unc-52
mig-6
unc-54
myo-3
dim-1
sur-5
Intestine
Neurons
Hypodermis
Muscle
Ubiquitous

Figure S2. Measurement of polyQ and Aβ_{1-42} transcript levels and validation of intestine-specific RNA-seq analysis. Related to Figures 2 and 3.

(A) qPCR of YFP transcript levels in animals expressing (i) neuronal polyQ$_{40}$::YFP, (ii) intestinal polyQ$_{40}$::YFP and (iii) muscle polyQ$_{35}$::YFP expressed with and without tissue-specific *xbp-1s.* YFP expression was measured at day 1 and day 4 of adulthood and normalized to day 1 polyQ alone. Significance was measured by one-way ANOVA with Tukey's multiple comparisons test.

(B) qPCR of Aβ_{1-42} transcript levels in animals expressing (i) neuronal Aβ_{1-42} and (ii) muscle Aβ_{1-42} with and without neuronal and intestinal *xbp-1s.* Aβ_{1-42} expression was measured at day 1 of adulthood and *xbp-1s*-expressing animals normalized to Aβ_{1-42} alone. Significance was measured by one-way ANOVA with Tukey's multiple comparisons test.

(C) Workflow of intestine-specific RNA-seq analysis. Cells from nematodes expressing the *ges-1p::GFP* intestinal marker are dissociated and collected using fluorescence-activated cell sorting (FACS). RNA is then extracted and amplified using the Ovation RNA-Seq System V2 before library preparation using the Ovation® Ultralow Library System. This RNA is sequenced and analyzed by intensity difference filtration to identify genes that are differentially regulated between *ges-1p::GFP* worms with and without neuronal *xbp-1s*. A confocal overlay micrograph of *ges-1p::GFP* worms is shown at X10 magnification.

(D) qPCR of *xbp-1s* transcript levels in intestinal cells of wild type and *rab-3p::xbp-1s*- expressing animals. Intestinal cells were isolated at day 1 of

adulthood from animals expressing *ges-1p::GFP* with and without *rab-3p::xbp-1s*, *xbp-1s* transcript levels measured, and *xbp-1*-expressing animals normalized to *ges-1p::GFP* alone. Significance was measured by one-way ANOVA with Tukey's multiple comparisons test, **p<0.01.

(E) Box and whisker plots of intestinal, muscle and neuronal gene expression levels in the RNA-seq dataset. Tissue-specific genes were identified using the Princeton "Tissue-specific expression predictions for *C. elegans*" database (http://worm-tissue.princeton.edu/search/download) and the distribution of expression levels of these genes in RNA-seq samples plotted. Significance between intestine-specific and muscle- or neuron-specific gene categories was measured by student's t-test, using expression of each tissue-specific gene as one sample, ****p<0.0001.

(F) Validation of intestinal enrichment by qRT-PCR analysis of tissue-specific transcripts. Levels of 4 intestinal transcripts - *gly-19, elt-2, ges-1, vha-6*; 3 neuronal transcripts - *rab-3, unc-119, rgef-1*; 3 hypodermal transcripts – *elt-1, unc-52, mig-6*; 3 muscle transcripts - *unc-54, myo-3, dim-1*; and 1 ubiquitous transcript - *sur-5*; were measured by qRT-PCR in RNA extracted from *ges-1p::GFP*-expressing isolated intestinal cells, and in RNA extracted from non-intestinal GFP-negative cells. Graphs represent mean transcript levels in sorted intestinal cells normalized to levels of each transcript in GFP-negative cells, from 3 independent biological replicates. Error bars represent standard error of the mean (SEM). Significance of enrichment was assessed by one-way ANOVA with Tukey's multiple comparisons test, *p<0.05, **p<0.001, ****p<0.0001.

A

Upregulated lysosomal gene	XBP-1s promoter binding sites (5'--3')	HLH-30 promoter binding sites (5'--3')
asp-3	ACGT core (-86 to -80)	-
cpr-1	ETS domain (-316 to -311; -88 to -83); ACGT core (-1975 to -1969; -730 to -724); UPRE B element (-1979 to -1969)	CLEAR domain (-152 to -147)
cpr-2	CCACG box (-827 to -822); ACGT core (-702 to -696)	-
cpr-4	ETS domain (-1859 to -1854); CCACG box (-1013 to -1008); ACGT core (-1042 to -1036; -459 to -453)	-
cpr-5	-	-
imp-2	CAAT box (-341 to -336)	CLEAR domain (-311 to -306)
F57F5.1	ACGT core (-373 to -367; -370 to -364)	CLEAR domain (-53 to -48)
aagr-3	CAAT box (+28 to +33); ACGT core (-427 to -421)	-
asah-1	CCACG box (-274 to -269; -82 to -77); ACGT core (-82 to -76)	CLEAR domain (-80 to -75)
gba-2	CAAT box (-802 to -797); ETS domain (-507 to -502)	-
hex-1	CAAT box (-353 to -348; -212 to -207); CCACG box (-1494 to -1489)	-
lipl-1	CCACG box (-985 to -980)	CLEAR domain (-444 to -439)
lipl-5	ACGT core (-1091 to -1085)	CLEAR domain (-51 to -46)
spp-10	-	CLEAR domain (-847 to -842)
nuc-1	ETS domain (-1617 to -1612); ACGT core (-295 to -289)	-
cdr-1	-	-
lys-4	-	CLEAR domain (-838 to -833)
lys-8	-	-
ctns-1	ETS domain (-1559 to -1554; -1827 to -1822)	-
vha-18	-	-
blos-8	CAAT box (-1430 to -1425)	-

Binding sites
CAAT box (5' -CCAATC- 3')
ETS domain (5' -CGGAAG- 3')
CCACG box (5' -GCCACG- 3')
ACGT core (5' -G(C/T)(C/G)ACGT- 3')
UPRE A element (5' -CGACGTGG- 3')
UPRE B element (5' -GTGACGTG- 3')
CLEAR domain (5' -CACGTG- 3')

B

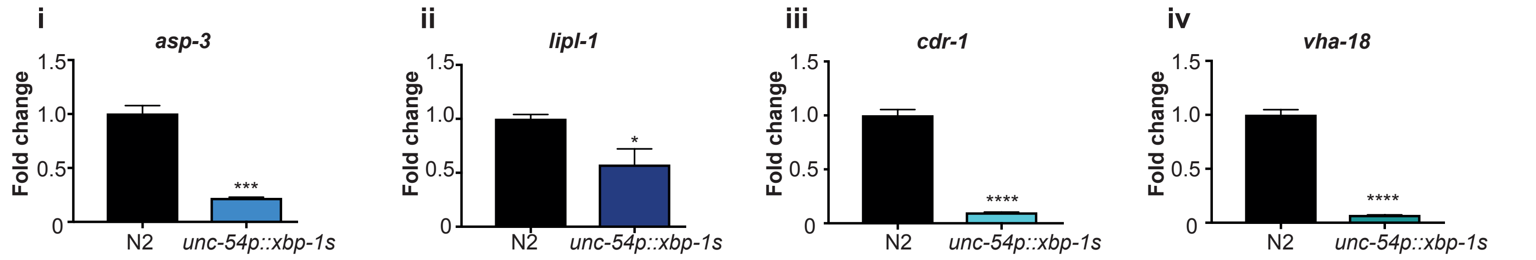

Figure S3. Lysosomal genes may be direct and tissue-specific targets of *xbp-1s*. Related to Figure 3.

(A) *xbp-1s* and *hlh-30* binding sites in the promoters of lysosomal genes upregulated in the intestines of neuronal *xbp-1s*-expressing animals. Sites listed in the legend were searched for within promoter sequences as defined by the Harvard Promoterome Database.

(B) qRT-PCR of lysosomal gene transcript levels in all-tissue lysates from animals expressing *xbp-1s* in muscle cells relative to N2 nematodes: (i) *asp-3*, (ii) *lipl-1*, (iii) *cdr-1*, (iv) *vha-18*. Transcript levels were measured at day 1 of adulthood and *xbp-1*-expressing animals normalized to N2. Significance was measured by one-way ANOVA with Tukey's multiple comparisons test, $*p<0.05$, $***p<0.001$, $****p<0.0001$.

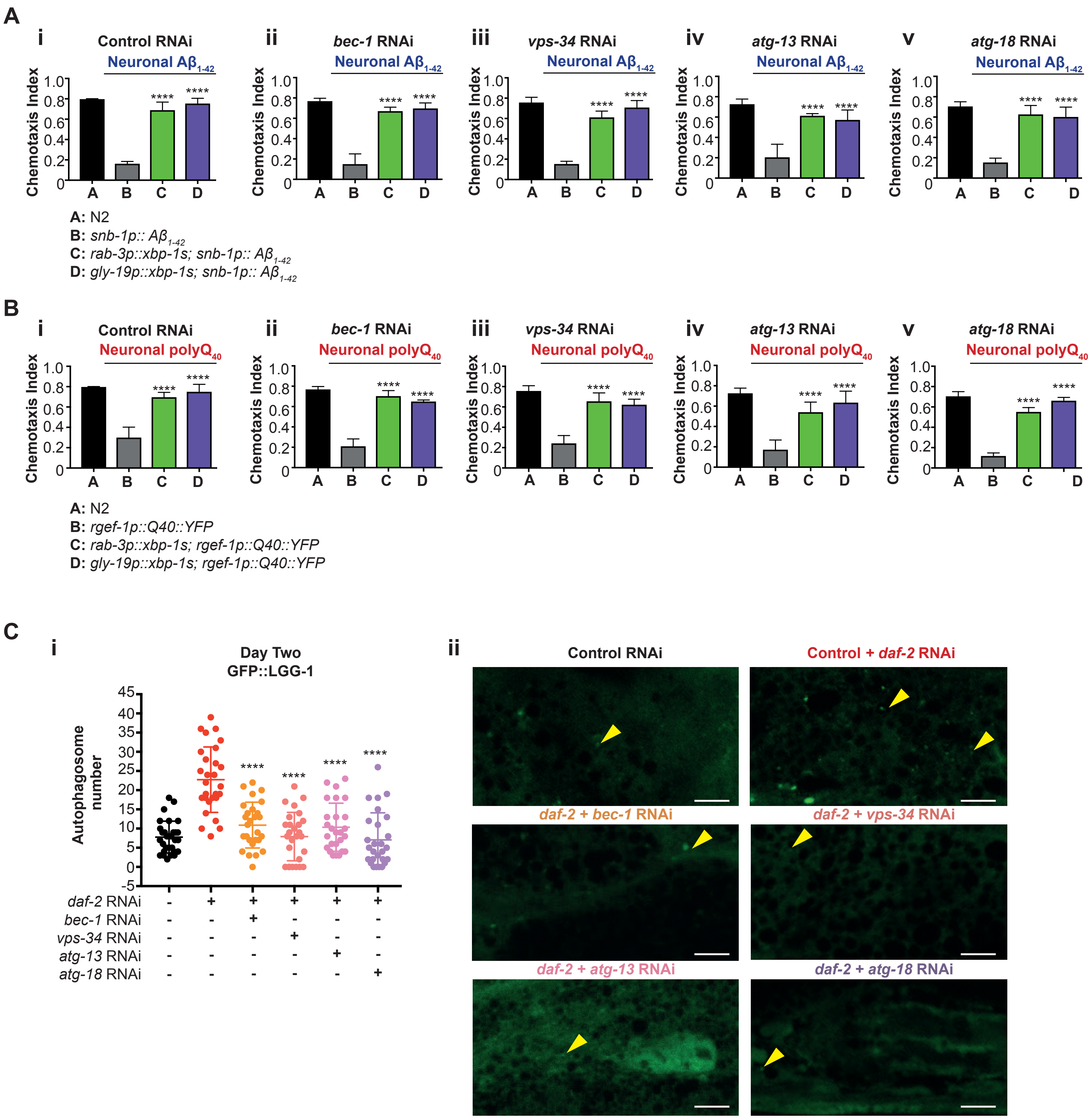

A
i Control RNAi
Neuronal Aβ1-42
ii bec-1 RNAi
iii vps-34 RNAi
iv atg-13 RNAi
v atg-18 RNAi
Chemotaxis Index
A B C D
****
A: N2
B: snb-1p:: Aβ1-42
C: rab-3p::xbp-1s; snb-1p:: Aβ1-42
D: gly-19p::xbp-1s; snb-1p:: Aβ1-42
B
Neuronal polyQ40
A: N2
B: rgef-1p::Q40::YFP
C: rab-3p::xbp-1s; rgef-1p::Q40::YFP
D: gly-19p::xbp-1s; rgef-1p::Q40::YFP
C
i Day Two
GFP::LGG-1
Autophagosome number
daf-2 RNAi
bec-1 RNAi
vps-34 RNAi
atg-13 RNAi
atg-18 RNAi
ii Control RNAi
Control + daf-2 RNAi
daf-2 + bec-1 RNAi
daf-2 + vps-34 RNAi
daf-2 + atg-13 RNAi
daf-2 + atg-18 RNAi

Figure S4. Protection from proteotoxicity in *xbp-1s*-expressing animals does not depend upon genes involved in autophagy. Related to Figure 4.

(A) Chemotaxis ability in animals expressing $A\beta_{1\text{-}42}$ in neurons in combination with neuronal and intestinal *xbp-1s*, grown on (i) control (empty vector), (ii) *bec-1*, (iii) *vps-34*, (iv) *atg-13*, or (v) *atg-18* RNAi. Graphs represent mean chemotaxis index ± SD. N = 80-150 animals per assay, and each assay was independently replicated 3 times. Significance between neuronal $A\beta_{1\text{-}42}$ (B) and *xbp-1s*-expressing (C/D) strains was assessed by two-way ANOVA with Dunnett's multiple comparisons test, ****$p<0.0001$.

(B) Chemotaxis ability in animals expressing $polyQ_{40}$ in neurons in combination with neuronal and intestinal *xbp-1s*, grown on (i) control (empty vector), (ii) *bec-1*, (iii) *vps-34*, (iv) *atg-13*, or (v) *atg-18* RNAi. Graphs represent mean chemotaxis index ± SD. N = 80-170 animals per assay, and each assay was independently replicated 3 times. Significance between neuronal $polyQ_{40}$ (B) and *xbp-1s*-expressing (C/D) strains was assessed by two-way ANOVA with Dunnett's multiple comparisons test, ****$p<0.0001$.

(C) (i) Quantification of autophagic vesicles. Animals were grown on control, control + *daf-2*, *daf-2* + *bec-1*, *daf-2* + *vps-34*, *daf-2* + *atg-13*, or *daf-2* + *atg-18* RNAi. GFP::LGG-1-positive punctae were imaged at X63 magnification and counted in 25-30 worms per genotype at day 2 of adulthood using ImageJ. Data are derived from 3 independent biological replicates. Statistical analysis was performed relative to control + *daf-2* RNAi, using one-way ANOVA with Tukey's multiple comparisons test, ****$p<0.0001$.

(ii) Representative confocal images of the intestine of animals expressing GFP::LGG-1, grown on control, control + *daf-2*, *daf-2* + *bec-1*, *daf-2* + *vps-34*,

daf-2 + *atg-13*, or *daf-2* + *atg-18* RNAi. Animals were imaged at day 2 of adulthood, at X63 magnification. Scale bar = 5 μm.

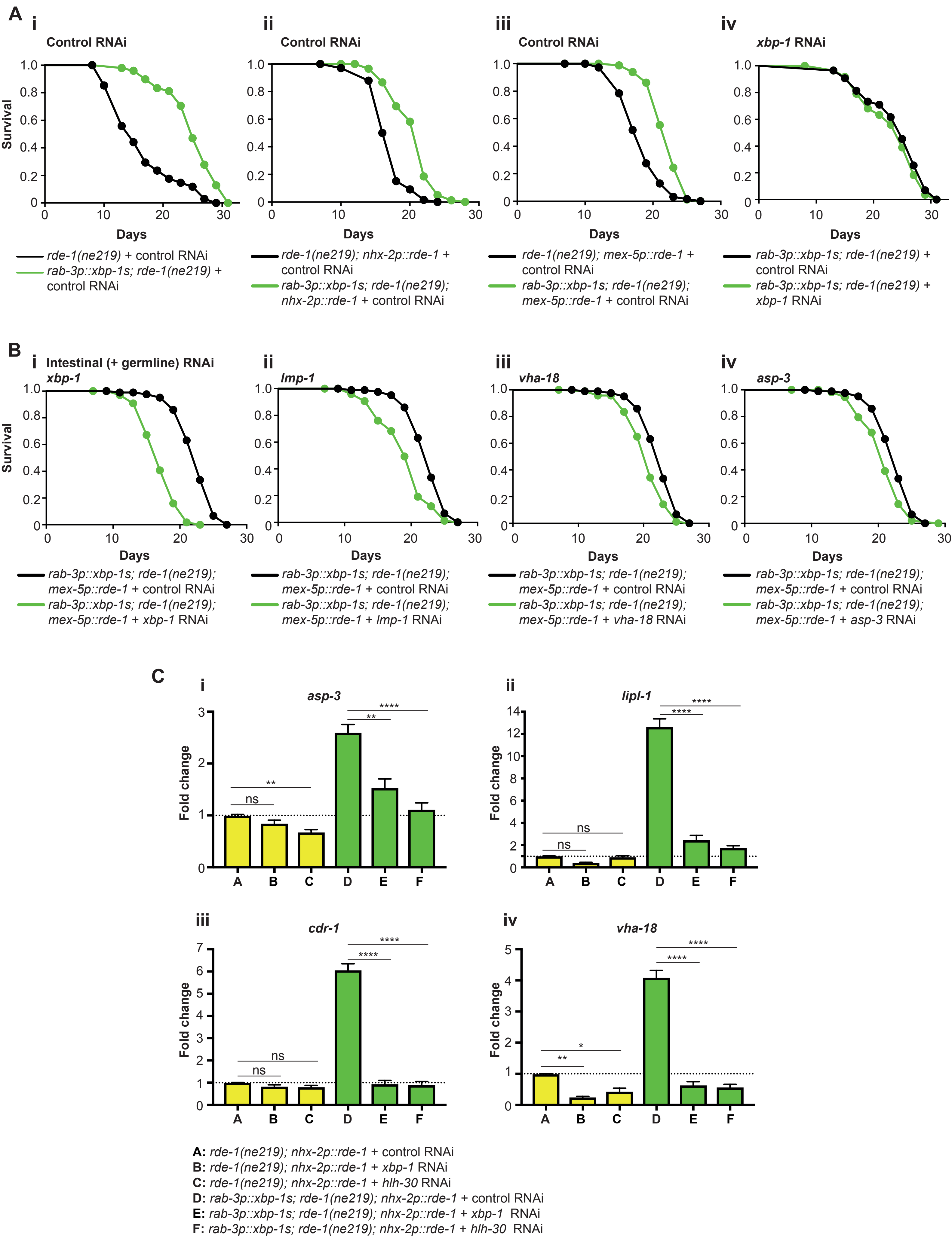

A
i Control RNAi
ii Control RNAi
iii Control RNAi
iv xbp-1 RNAi
Survival
Days
rde-1(ne219) + control RNAi
rab-3p::xbp-1s; rde-1(ne219) + control RNAi
rde-1(ne219); nhx-2p::rde-1 + control RNAi
rab-3p::xbp-1s; rde-1(ne219); nhx-2p::rde-1 + control RNAi
rde-1(ne219); mex-5p::rde-1 + control RNAi
rab-3p::xbp-1s; rde-1(ne219); mex-5p::rde-1 + control RNAi
rab-3p::xbp-1s; rde-1(ne219) + control RNAi
rab-3p::xbp-1s; rde-1(ne219) + xbp-1 RNAi
B
i Intestinal (+ germline) RNAi
xbp-1
ii lmp-1
iii vha-18
iv asp-3
Survival
Days
rab-3p::xbp-1s; rde-1(ne219); mex-5p::rde-1 + control RNAi
rab-3p::xbp-1s; rde-1(ne219); mex-5p::rde-1 + xbp-1 RNAi
rab-3p::xbp-1s; rde-1(ne219); mex-5p::rde-1 + lmp-1 RNAi
rab-3p::xbp-1s; rde-1(ne219); mex-5p::rde-1 + vha-18 RNAi
rab-3p::xbp-1s; rde-1(ne219); mex-5p::rde-1 + asp-3 RNAi
C
i asp-3
ii lipl-1
iii cdr-1
iv vha-18
Fold change
A B C D E F
ns
**
****
*
A: rde-1(ne219); nhx-2p::rde-1 + control RNAi
B: rde-1(ne219); nhx-2p::rde-1 + xbp-1 RNAi
C: rde-1(ne219); nhx-2p::rde-1 + hlh-30 RNAi
D: rab-3p::xbp-1s; rde-1(ne219); nhx-2p::rde-1 + control RNAi
E: rab-3p::xbp-1s; rde-1(ne219); nhx-2p::rde-1 + xbp-1 RNAi
F: rab-3p::xbp-1s; rde-1(ne219); nhx-2p::rde-1 + hlh-30 RNAi

Figure S5. Validation of intestine-specific RNAi. Related to Figure 5 and Table S1.

(A) (i) Lifespan analysis of *rde(n219)* and *rab-3p::xbp-1s; rde(n219)* animals grown on control (empty vector) RNAi. *rde(n219)*, control (black), median lifespan 15 days; *rab-3p::xbp-1s; rde(n219)*, control (green), median lifespan 25 days, p<0.0001. Graph plotted as a Kaplan-Meier survival curve, P value calculated by Mantel-Cox log-rank test; N = 80-120 animals per lifespan.

(ii) Lifespan analysis of *rde(n219); nhx-2p::rde-1* and *rab-3p::xbp-1s; rde(n219); nhx-2p::rde-1* animals grown on control (empty vector) RNAi. *rde(n219); nhx-2p::rde-1*, control (black), median lifespan 18 days; *rab-3p::xbp-1s; rde(n219); nhx-2p::rde-1*, control (green), median lifespan 22 days, p<0.0001. Graph plotted as a Kaplan-Meier survival curve, P value calculated by Mantel-Cox log-rank test; N = 80-120 animals per lifespan.

(iii) Lifespan analysis of *rde(n219); mex-5p::rde-1* and *rab-3p::xbp-1s; rde(n219); mex-5p::rde-1* animals grown on control (empty vector) RNAi. *rde(n219); mex-5p::rde-1*, control (black), median lifespan 19 days; *rab-3p::xbp-1s; rde(n219); mex-5p::rde-1*, control (green), median lifespan 23 days, p<0.0001. Graph plotted as a Kaplan-Meier survival curve, P value calculated by Mantel-Cox log-rank test; N = 80-120 animals per lifespan.

(iv) Lifespan analysis of *rab-3p::xbp-1s; rde(n219)* animals grown on control (empty vector) and *xbp-1* RNAi. *rab-3p::xbp-1s; rde(n219)*, control (black), median lifespan 25 days; *rab-3p::xbp-1s; rde(n219)*, *xbp-1* (green), median lifespan 25 days, p=0.2281. Graph plotted as a Kaplan-Meier survival curve, P value calculated by Mantel-Cox log-rank test; N = 80-120 animals per lifespan.

(B) Lifespan analysis of *rab-3p::xbp-1s; rde(n219); mex-5p::rde-1* animals grown on control (empty vector) or (i) *xbp-1*, (ii) *lmp-1*, (iii) *vha-18*, or (iv) *asp-3* RNAi. Graphs were plotted as Kaplan-Meier survival curves, P values were calculated by Mantel-Cox log-rank test.

(i) *rab-3p::xbp-1s; rde(n219); mex-5p::rde-1*, control (black), median lifespan 23 days; *rab-3p::xbp-1s; rde(n219); mex-5p::rde-1*, *xbp-1* (green), median lifespan 17 days, p<0.0001.

(ii) *rab-3p::xbp-1s; rde(n219); mex-5p::rde-1*, control (black), median lifespan 23 days; *rab-3p::xbp-1s; rde(n219); mex-5p::rde-1*, *lmp-1* (green), median lifespan 19 days, p<0.0001.

(iii) *rab-3p::xbp-1s; rde(n219); mex-5p::rde-1*, control (black), median lifespan 23 days; *rab-3p::xbp-1s; rde(n219); mex-5p::rde-1*, *vha-18* (green), median lifespan 21 days, p<0.0001.

(iv) *rab-3p::xbp-1s; rde(n219); mex-5p::rde-1*, control (black), median lifespan 23 days; *rab-3p::xbp-1s; rde(n219); mex-5p::rde-1*, *asp-3* (green), median lifespan 21 days, p<0.01.

(C) qPCR of (i) *asp-3*, (ii) *lipl-1*, (iii) *cdr-1* and (iv) *vha-18* transcript levels at day 1 of adulthood in *rde-1(ne219); nhx-2p::rde-1* animals, with and without *rab-3p::xbp-1s* expression, grown on control, *xbp-1* or *hlh-30* RNAi. Expression was normalized to *rde-1(ne219); nhx-2p::rde-1* on control RNAi and significance measured by one-way ANOVA with Tukey's multiple comparisons test, ns = not significant, *p<0.05, **p<0.01, ****p<0.0001.

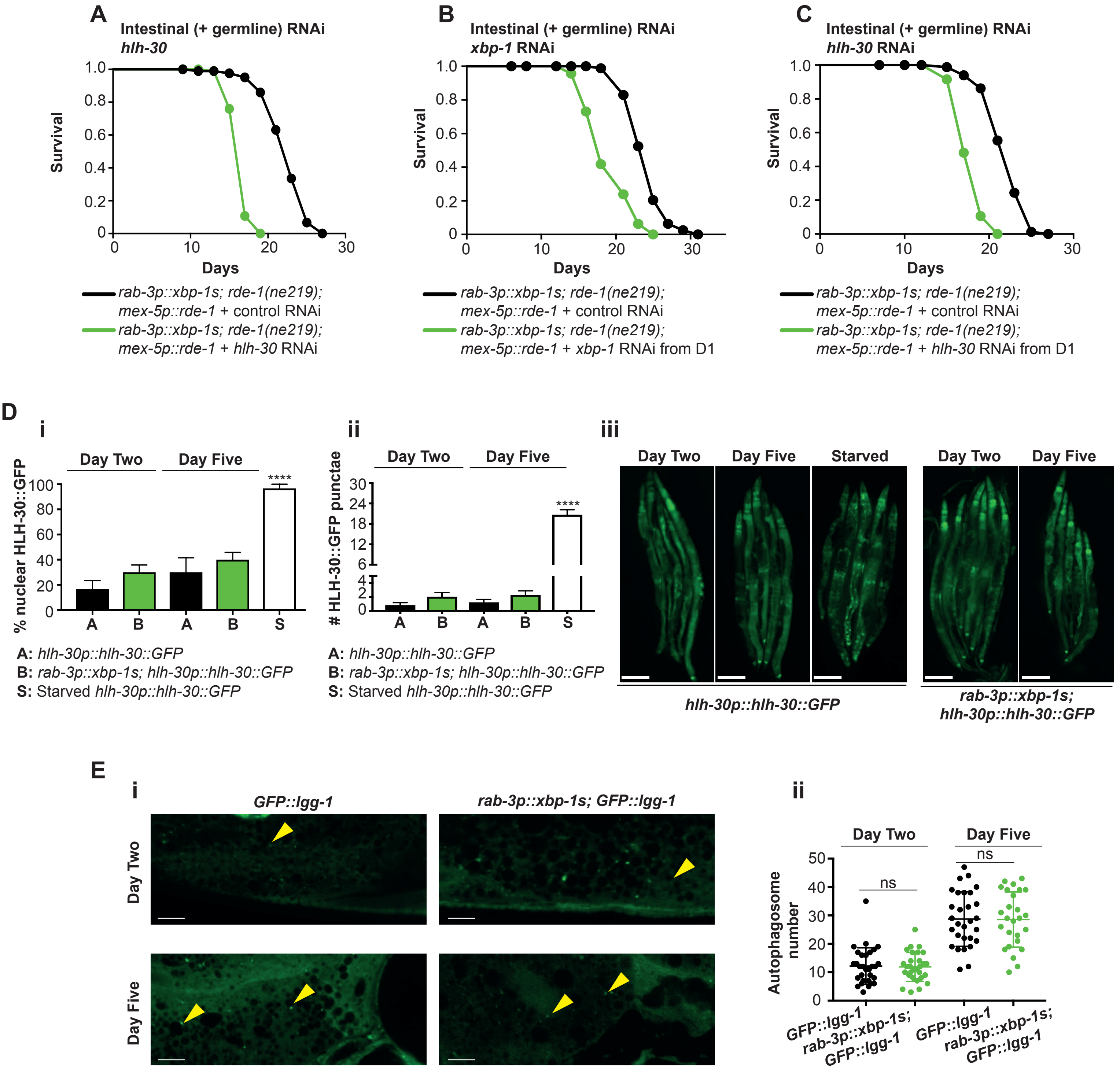
A
Intestinal (+ germline) RNAi
hlh-30
Survival
Days
rab-3p::xbp-1s; rde-1(ne219); mex-5p::rde-1 + control RNAi
rab-3p::xbp-1s; rde-1(ne219); mex-5p::rde-1 + hlh-30 RNAi
B
Intestinal (+ germline) RNAi
xbp-1 RNAi
rab-3p::xbp-1s; rde-1(ne219); mex-5p::rde-1 + control RNAi
rab-3p::xbp-1s; rde-1(ne219); mex-5p::rde-1 + xbp-1 RNAi from D1
C
Intestinal (+ germline) RNAi
hlh-30 RNAi
rab-3p::xbp-1s; rde-1(ne219); mex-5p::rde-1 + control RNAi
rab-3p::xbp-1s; rde-1(ne219); mex-5p::rde-1 + hlh-30 RNAi from D1
D
i
Day Two
Day Five
% nuclear HLH-30::GFP
****
A: hlh-30p::hlh-30::GFP
B: rab-3p::xbp-1s; hlh-30p::hlh-30::GFP
S: Starved hlh-30p::hlh-30::GFP
ii
HLH-30::GFP punctae
****
A: hlh-30p::hlh-30::GFP
B: rab-3p::xbp-1s; hlh-30p::hlh-30::GFP
S: Starved hlh-30p::hlh-30::GFP
iii
Day Two
Day Five
Starved
hlh-30p::hlh-30::GFP
rab-3p::xbp-1s; hlh-30p::hlh-30::GFP
E
i
GFP::lgg-1
rab-3p::xbp-1s; GFP::lgg-1
Day Two
Day Five
ii
Autophagosome number
ns
ns
GFP::lgg-1
rab-3p::xbp-1s; GFP::lgg-1

Figure S6. Analysis of HLH-30 and LGG-1 localization in intestinal cells. Related to Figures 5 and 6 and Table S1.

(A) Lifespan analysis of *rab-3p::xbp-1s; rde(n219); mex-5p::rde-1* animals grown on control (empty vector) or *hlh-30* RNAi. *rab-3p::xbp-1s; rde(n219); mex-5p::rde-1*, control (black), median lifespan 23 days; *rab-3p::xbp-1s; rde(n219); mex-5p::rde-1*, *hlh-30* (green), median lifespan 17 days, p<0.0001. Graphs were plotted as Kaplan-Meier survival curves, P values were calculated by Mantel-Cox log-rank test.

(B) Lifespan analysis of *rab-3p::xbp-1s; rde(n219); mex-5p::rde-1* animals grown on control (empty vector) RNAi throughout life, or on control (empty vector) RNAi until day 1 of adulthood and transferred to *xbp-1* RNAi from this age. *rab-3p::xbp-1s; rde(n219); mex-5p::rde-1*, control (black), median lifespan 25 days; *rab-3p::xbp-1s; rde(n219); mex-5p::rde-1*, *xbp-1* from adulthood (green), median lifespan 18 days, p<0.0001. Graph plotted as a Kaplan-Meier survival curve, P value calculated by Mantel-Cox log-rank test; N = 80-120 animals per lifespan.

(C) Lifespan analysis of *rab-3p::xbp-1s; rde(n219); mex-5p::rde-1* animals grown on control (empty vector) RNAi throughout life, or on control (empty vector) RNAi until day 1 of adulthood and transferred to *hlh-30* RNAi from this age. *rab-3p::xbp-1s; rde(n219); mex-5p::rde-1*, control (black), median lifespan 23 days; *rab-3p::xbp-1s; rde(n219); mex-5p::rde-1*, *hlh-30* from adulthood (green), median lifespan 17 days, p<0.0001. Graph plotted as a Kaplan-Meier survival curve, P value calculated by Mantel-Cox log-rank test; N = 80-120 animals per lifespan.

(D) (i) Quantification of HLH-30::GFP nuclear localization. Numbers of animals with nuclear HLH-30::GFP in the intestine were quantified at day 2 and day 5 of adulthood (except starved animals which were quantified at day 2 only); graphs represent mean percentage of animals with nuclear HLH-30::GFP ± SEM. N = 30 animals per assay, independently replicated 3 times. Significance was assessed by one-way ANOVA with Tukey's multiple comparisons test, ****p<0.0001.

(ii) Quantification of HLH-30::GFP-positive nuclei per animal. Numbers of HLH-30::GFP-positive nuclei in the intestine were quantified per animal at day 2 and day 5 of adulthood (except starved animals which were quantified at day 2 only); graphs represent mean percentage of animals with nuclear HLH-30::GFP ± SEM. N = 30 animals per assay, independently replicated 3 times. Significance was assessed by one way ANOVA with Tukey's multiple comparisons test, ****p<0.0001.

(iii) Confocal images of animals expressing HLH-30::GFP with and without *rab-3p::xbp-1s* at day 2 and day 5 of adulthood, alongside starved animals expressing HLH-30::GFP at day 2. Scale bar = 100 μm.

(E) (i) Representative confocal images of the intestine of animals expressing GFP::LGG-1, with and without *rab-3p::xbp-1s*. Animals were grown on OP50 and imaged at days 2 and 5 of adulthood. Imaging was performed at X63 magnification. Arrowheads indicate representative autophagosomes. Scale bar = 5 µm.

(ii) Quantification of autophagic vesicles. GFP::LGG-1-positive punctae were imaged at X63 magnification and counted in 10-15 worms per genotype at day 2 and day 5 of adulthood using ImageJ. Data are derived from 3

independent biological replicates. Statistical analysis was performed using one-way ANOVA with Tukey’s multiple comparisons test. ns = not significant.

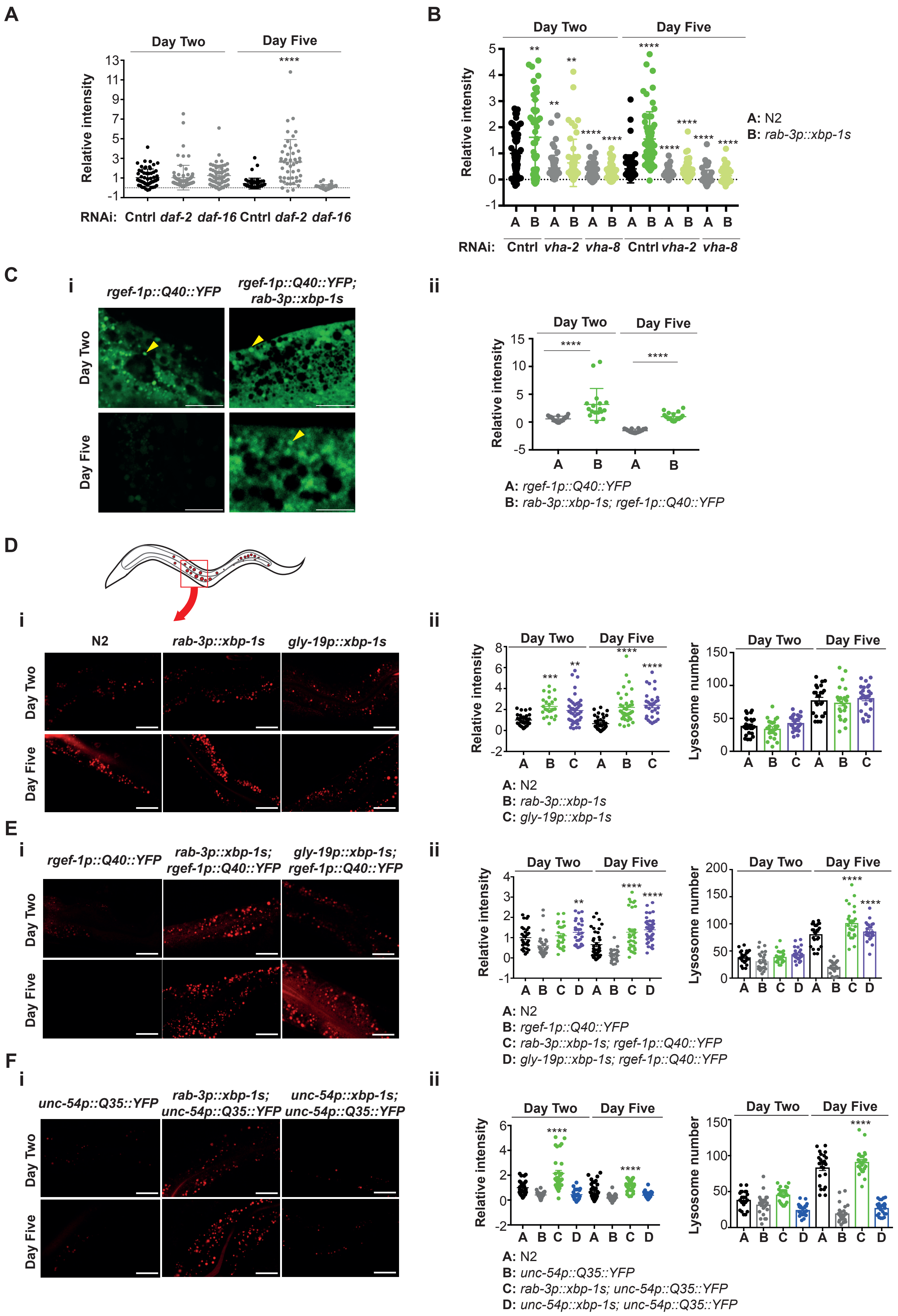
A
Day Two
Day Five
Relative intensity
RNAi: Cntrl daf-2 daf-16 Cntrl daf-2 daf-16
B
Day Two
Day Five
Relative intensity
A: N2
B: rab-3p::xbp-1s
RNAi: Cntrl vha-2 vha-8 Cntrl vha-2 vha-8
C
i
rgef-1p::Q40::YFP
rgef-1p::Q40::YFP; rab-3p::xbp-1s
Day Two
Day Five
ii
Day Two
Day Five
Relative intensity
A: rgef-1p::Q40::YFP
B: rab-3p::xbp-1s; rgef-1p::Q40::YFP
D
i
N2
rab-3p::xbp-1s
gly-19p::xbp-1s
Day Two
Day Five
ii
Day Two
Day Five
Relative intensity
Lysosome number
A: N2
B: rab-3p::xbp-1s
C: gly-19p::xbp-1s
E
i
rgef-1p::Q40::YFP
rab-3p::xbp-1s; rgef-1p::Q40::YFP
gly-19p::xbp-1s; rgef-1p::Q40::YFP
Day Two
Day Five
ii
Day Two
Day Five
Relative intensity
Lysosome number
A: N2
B: rgef-1p::Q40::YFP
C: rab-3p::xbp-1s; rgef-1p::Q40::YFP
D: gly-19p::xbp-1s; rgef-1p::Q40::YFP
F
i
unc-54p::Q35::YFP
rab-3p::xbp-1s; unc-54p::Q35::YFP
unc-54p::xbp-1s; unc-54p::Q35::YFP
Day Two
Day Five
ii
Day Two
Day Five
Relative intensity
Lysosome number
A: N2
B: unc-54p::Q35::YFP
C: rab-3p::xbp-1s; unc-54p::Q35::YFP
D: unc-54p::xbp-1s; unc-54p::Q35::YFP

Figure S7. Validation of lysosomal acidity staining and analysis of lysosomal acidity in animals expressing polyQ expansions. Related to Figure 7.

(A) Quantification of lysosomal acidity in the intestine of animals grown on control (empty vector), *daf-2* or *daf-16* RNAi. Animals were transferred to plates containing cDCFDA 16 hours prior to imaging. Using X63 magnification, imaging was conducted at day 2 and day 5 of adulthood. Quantification of fluorescence from 3 experiments was carried out using ImageJ. Plots represent 10 animals per replicate. Significance was assessed at each time point by an ordinary one-way ANOVA with Tukey's multiple comparisons, ****$p<0.0001$.

(B) Quantification of lysosomal acidity in the intestine of N2 and *rab-3p::xbp-1s*–expressing animals grown on control, *vha-2* or *vha-8* RNAi. Animals were transferred to plates containing cDCFDA 16 hours prior to imaging. Using X63 magnification, imaging was conducted at day 2 and day 5 of adulthood. Quantification of fluorescence from 3 experiments was carried out using ImageJ. Plots represent 10 animals per replicate. Significance was assessed in each genotype relative to control RNAi at both time points by ordinary one-way ANOVA with Tukey's multiple comparisons, **$p<0.01$, ****$p<0.0001$.

(C) (i) Confocal imaging of acidic lysosomes in the intestine of neuronal polyQ$_{40}$ animals, with and without neuronal *xbp-1s*. Animals were grown on OP50 and transferred to plates containing cDCFDA 16 hours prior to imaging. Using X63 magnification, imaging was conducted at day 2 and day 5 of adulthood. Scale bar = 10 μm.

(ii) Quantification of lysosomal acidity in the intestine of neuronal polyQ$_{40}$ animals, with and without neuronal *xbp-1s*. Animals were grown on OP50 and transferred to plates containing cDCFDA 16 hours prior to imaging and imaged as (D); quantification of fluorescence from 2 experiments was carried out using ImageJ. Plots represent 5-10 animals per replicate. Significance was assessed between N2 and *rab-3p::xbp-1s* at each time point by one-way ANOVA with Tukey's multiple comparisons, ****p<0.0001.

(D) (i) Confocal imaging of N2, *rab-3p::xbp-1s* and *gly-19p::xbp-1s* nematodes stained with LysoTracker Deep Red at days 2 and 5 of adulthood. Scale bar = 20 µm.

(ii) Quantification of intensity and number of Lysotracker-stained punctae in animals imaged as above. ImageJ was used to assess fluorescence intensity, which was normalized to N2. Each plot represents mean ± SEM from 3 independent biological replicates, N = 8-10 animals per replicate. Significance was calculated using one way ANOVA with Tukey's multiple comparisons test, **p<0.01, ***p<0.001, ****p<0.0001.

(E) (i) Confocal imaging of *rgef-1p::Q40::YFP* animals with or without *rab-3p::xbp-1s* and *gly-19p::xbp-1s,* stained with LysoTracker Deep Red at days 2 and 5 of adulthood. Scale bar = 20 µm.

(ii) Quantification of intensity and number of Lysotracker-stained punctae in *rgef-1p::Q40::YFP* animals imaged as above. ImageJ was used to assess fluorescence intensity, which was normalized to N2. Each plot represents mean ± SEM from 3 independent biological replicates, N = 8-10 animals per replicate. Significance was calculated using one way ANOVA with Tukey's multiple comparisons test, **p<0.01, ****p<0.0001.

(F) (i) Confocal imaging of *unc-54p::Q35::YFP* animals with or without *rab-3p::xbp-1s* and *unc-54p::xbp-1s,* stained with LysoTracker Deep Red at days 2 and 5 of adulthood. Scale bar = 20 μm.

(ii) Quantification of intensity and number of Lysotracker-stained punctae in *unc-54p::Q35::YFP* animals imaged as above. ImageJ was used to assess fluorescence intensity, which was normalized to N2. Each plot represents mean ± SEM from 3 independent biological replicates, N = 8-10 animals per replicate. Significance was calculated using one way ANOVA with Tukey's multiple comparisons test, ****p<0.0001.
